# Quantitative ‘Omics Analyses of Medium Chain Length Polyhydroxyalkanaote Metabolism in *Pseudomonas putida* LS46 Cultured with Waste Glycerol and Waste Fatty Acids

**DOI:** 10.1371/journal.pone.0142322

**Published:** 2015-11-06

**Authors:** Jilagamazhi Fu, Parveen Sharma, Vic Spicer, Oleg V. Krokhin, Xiangli Zhang, Brian Fristensky, Nazim Cicek, Richard Sparling, David. B. Levin

**Affiliations:** 1 Department of Biosystem Engineering, University of Manitoba, Winnipeg, Manitoba, Canada; 2 Department of Internal Medicine & Manitoba Centre for Proteomics and Systems Biology, University of Manitoba, Winnipeg, Manitoba, Canada; 3 Department of Plant Science, University of Manitoba, Winnipeg, Manitoba, Canada; 4 Department of Microbiology, University of Manitoba, Winnipeg, Manitoba, Canada; University Paris South, FRANCE

## Abstract

Transcriptomes and proteomes of *Pseudomonas putida* LS46 cultured with biodiesel-derived waste glycerol or waste free fatty acids, as sole carbon sources, were compared under conditions that were either permissive or non-permissive for synthesis of medium chain length polyhydroxyalkanoates (mcl-PHA). The objectives of this study were to elucidate mechanisms that influence activation of biopolymer synthesis, intra-cellular accumulation, and monomer composition, and determine if these were physiologically specific to the carbon sources used for growth of *P*. *putida* LS46. Active mcl-PHA synthesis by *P*. *putida* LS46 was associated with high expression levels of key mcl-PHA biosynthesis genes and/or gene products including monomer-supplying proteins, PHA synthases, and granule-associated proteins. ‘Omics data suggested that expression of these genes were regulated by different genetic mechanisms in *P*. *putida* LS46 cells in different physiological states, when cultured on the two waste carbon sources. Optimal polymer production by *P*. *putida* LS46 was primarily limited by less efficient glycerol metabolism during mcl-PHA synthesis on waste glycerol. Mapping the ‘Omics data to the mcl-PHA biosynthetic pathway revealed significant variations in gene expression, primarily involved in: 1) glycerol transportation; 2) enzymatic reactions that recycle reducing equivalents and produce key mcl-PHA biosynthesis pathway intermediates (e.g. NADH/NADPH, acetyl-CoA). Active synthesis of mcl-PHAs was observed during exponential phase in cultures with waste free fatty acids, and was associated with the fatty acid beta-oxidation pathway. A putative Thioesterase in the beta-oxidation pathway that may regulate the level of fatty acid beta-oxidation intermediates, and thus carbon flux to mcl-PHA biosynthesis, was highly up-regulated. Finally, the data suggested that differences in expression of selected fatty acid metabolism and mcl-PHA monomer-supplying enzymes may play a role in determining the monomer composition of mcl-PHA polymers. Understanding the relationships between genome content, gene and gene product expression, and how these factors influence polymer synthesis, will aid in optimization of mcl-PHA production by *P*. *putida* LS46 using biodiesel waste streams.

## Introduction

Medium chain length polyhydroxyalkanoates (mcl-PHAs) are mostly produced by bacteria in the genus *Pseudomonas* as reserve sources of carbon and energy under conditions of nutritional stress [1]. Mcl-PHA synthesis by *Pseudomonas putida* has been particularly well studied [2]. Mcl-PHA polymers may be used to manufacture biodegradable plastics and resins, but large-scale production of these polymers is currently hindered by high product costs, of which substrate cost is a major component [1]. A number of studies have explored the use of microorganisms to convert agro-industrial waste streams into value-added PHA polymers [3,4]. The by-products from industrial biodiesel production, such as biodiesel-derived glycerol and biodiesel-derived free fatty acids, contain certain amount of impurities making them less useful for other downstream industrial applications. For example, waste glycerol contains methanol, residual free fatty acids, sodium or potassium soaps derived from the catalysts used to synthesize biodiesel, and numbers of identified heavy metals [5,6], Waste glycerol is normally refined in order to use in food, cosmetics, and pharmaceutical industrial. Non-refined waste glycerol was used as animal feedstuff, but concerns still remain regarding the acceptable level of the impurities [7,8]. Biological conversion of biodiesel derived waste carbon sources into high-value added product, such as synthesis of mcl-PHA by *Pseudomonas putida* [9,10], is of great interests currently. Understanding the effects of these low cost “waste” carbon sources on the metabolism of *P*. *putida* in general, and mcl-PHA synthesis pathways in particular, will provide a rational basis for optimization of fermentation strategies for large-scale mcl-PHA production.

High throughput studies of biological systems at the ‘Omics level (transcriptomics, proteomics) provides an informative and rather complete “snapshot” of gene expression profiles under specific growth conditions. The advantages of this approach include, but are not limited to: i) identification of pathway genes that are actively involved in metabolic processes; ii) differentiation of active genes from redundantly encoded genes in the genome; iii) prediction of carbon and electron flow, which influence metabolic flux, and therefore determine end-product synthesis patterns; and iv) insight into mechanisms of gene regulation and regulatory variation at transcriptional or translational level [11].

In case of mcl-PHA production from *P*. *putida*, ‘Omics data have previously been used to propose a strategy for optimizing polymer biosynthesis from glucose [2], to evaluate gene expression in *P*. *putida* grown during growth on pure fatty acids [12,13]. The transcriptome of *P*. *putida* KT2440 has reported for pure reagent grade glycerol cultures, but not specifically for mcl-PHA synthesis and related pathways [14,15]. A systematic analysis of gene expression pertinent to mcl-PHA biosynthesis by *P*. *putida*, cultured with waste glycerol and waste free fatty acids, at both the transcriptome and proteome level, has not yet been reported.

In order to gain further insight into the mechanisms that influence mcl-PHA, we have studied two mcl-PHA producing conditions in *P*. *putida* LS46. Previously, in *P*. *putida* LS46 cultured with biodiesel-derived glycerol, the mcl-PHA synthesis phase is restricted to the stationary phase, under conditions of excess carbon and limiting nitrogen concentrations. In *P*. *putida* LS46 cultured with biodiesel-derived free fatty acids, however, mcl-PHA synthesis was possible under excess nitrogen condition, although the maximum production occurred after nitrogen depletion [9]. In this work, we have applied quantitative transcriptomics by RNA sequencing (RNAseq) and proteomics (1D-LC-MS) to understand: a) the activation of mcl-PHA biosynthesis in cultures containing biodiesel-derived glycerol versus biodiesel-derived free fatty acids; and b) the potential genetic targets that effect intra-cellular polymer accumulation and monomer composition.

## Material and Methods

### Bacterial strain and culture conditions

*Pseudomonas putida* LS46 was isolated from a local wastewater treatment plant in Winnipeg, Manitoba, Canada. The strain was deposited with International Depository Authority of Canada (IDAC) at the National Microbiology Laboratory, Health Canada Culture Collection (NML-HCCC), WDCM number 840 [16]. Crude biodiesel-derived (waste) glycerol (WG) and (waste) free fatty acids (WFA) were obtained from the Renewable Energy Group LLC (Danville, Illinois, USA). *P*. *putida* LS46 was cultured in Ramsay’s medium supplemented with either 30 g/L waste glycerol, or 1 v/v% (about 9 g/L) of waste fatty acids as studied in our previous work [9]. The components of Ramsay’s medium are (per liter): 6.7 g of Na_2_HPO_4_·7H_2_O, 1.5 g of KH_2_PO_4_, 1.0 g of (NH_4_)_2_SO_4_, 0.2 g of MgSO_4_·7H_2_0, 60 mg of ferrous ammonium citrate, 10 mg of CaCl_2_·2H_2_O, 1 ml of trace element solution. Each liter of trace element solution contains the following: 0.3 g of H_3_BO_3_, 0.2 g of CoCl_2_·6H_2_O, 0.1 g of ZnSO_4_·7H_2_O, 30 mg of MnCl_2_·4H_2_O, 30 mg of NaMoO_4_·2H_2_O, 20 mg of NiCl_2_·6H_2_O, 10 mg of CuSO_4_·5H_2_O. The major fatty acids of triacylglycerides in the biodiesel-derived waste fatty acids used as substrate in these experiments was previously reported [9], and were C16 (20.4%), C18:0 (11.5%), C18:1 (35.4%) and C18:2 (20.7%), based on the peak area percentage.

### Cell growth measurements

Dry cell weight was used as an indicator of the cell growth. Ten (10) mL cultures medium were collected in 50 mL falcon tubes during time-point sampling, centrifuged at 4000 x g for 10 min at room temperature, and washed with deionized water. The resulting wet cell pellets were dried in the oven at 60°C overnight before determining the dry cell weight.

### Nutrition consumption and mcl-PHA quantification

Residual glycerol in media was measured using a WATERS Breeze^TM^ 2 HPLC system (model number: MO915P795A) (Milford, MA, US). Pure glycerol was used an external standard for quantification purposes. Residual ammonia concentrations in the medium were measured using the Quikchem method 10-107-06-1-I, by flow injection analysis (Lachat Instrument, Colorado, USA). Qualitative and quantitative measurement of free fatty acids content of the WFA culture was carried out as our previous study [9]. Mcl-PHA production over time was measured using the same method as described previously [9]. Cells from overnight cultures of *P*. *putida* LS46 grown in WFA and use to inoculate fresh cultures for the experiments described below contained 10.74 wt % of mcl-PHA.

### Quantitative ‘Omics analysis

#### Sampling conditions for RNA and protein isolation

Samples of *P*. *putida* LS46 cells were collected during the exponential and stationary phases for waste glycerol cultures, and from the exponential phase only in waste free fatty acid cultures. Triplicate shake flask experiments were been carried out for physiological analysis (e.g. growth characterization, and polymer production). Subsequently, two replicates derived from the same triplicates shake-flasks experiments were taken for each of the above sampling conditions for RNAseq and proteomic analyses.

#### RNA isolation and RNAseq

Five (5) mL of cell culture were collected and centrifuged at 4,000 x g, to pellet the cells. Total RNA was extracted from the cell pellets using a PureLink^®^ RNA Mini Kit (Ambion^®^, Life Technologies, CA, USA). Isolated RNA samples were treated with Purelink^®^ DNase to remove DNA contamination. The concentration of RNA was determined using a Nanodrop 1000 spectrophotometer (Thermo fisher Scientific, MA, USA), and RNA integrity was analyzed by electrophoresis using an Experion system (Bio-Rad, CA, USA). RNA samples were shipped to and sequenced by the Genome Quebec Innovation Centre at McGill University (Montreal, Quebec) for creation of cDNA libraries and RNAseq using the Illumina HiSeq 2000 platform.

#### Protein isolation, digestion, and peptide purification

Culture media (40 mL) were collected, centrifuged at 4,000 x g, resuspended with Phosphate Buffer Solution (PBS) (0.144 g/L KH_2_PO_4_; 9 g/L NaCl; 0.795 g/L Na_2_HPO_4_∙7H_2_O, pH: 7.4) and centrifuged again at 4,000 x g. The PBS wash was repeated once more and the cell pellets were then stored at -80°C for later use. When all samples were collected, frozen pellets were re-dissolved in 4 mL distilled-deionized (DI) water, of which 1 mL was used for total protein isolation. One mL of Lysis Buffer (8% of sodium dodecyl sulphate, 200 mM dithiothreatol, 200 mM Tris-HCl, pH: 7.6) was added to each 1 mL sample of resuspended cell pellet and solution were mixed thoroughly by pipetting. The solutions were transferred to 10 mL Falcon tubes, which were then place in a boiling water bath for 5 minutes (min). After boiling, the samples were sonicated for 30 seconds (s), and then centrifuged at 16,000 x g at room temperature. 10 ul of supernatant was subjected to SDS-PAGE gel (**S1 Fig**). One mL of each supernatant was then transferred into an Eppendorf tube.

One mL of total protein was transferred to an Amicon Ultra-15 10K filter device (Millipore, Billerica, MA), which followed the purification, digestion, and subsequent peptide purification steps as mentioned in Gungormusler-Yilmaz *et al* 2014 [17] with little modification: the typsin to protein ration at 1:100 instead of 1:50. The purified peptides were lyophilized and re-dissolved in 0.1% formic acid in water for subsequent 1D-LC-MS analysis.

Protein and RNA identification: Transcriptomic analysis used an in-house alignment and quantitation engine using the R2 element of the Illumina paired-end read sets. Our algorithm detects exact 100-mer read alignments only, relying on the high quality of the HiSeq data to give sufficient counting statistics. One dimensional liquid chromatography followed by mass spectrometry (1D-LC-MS) proteomics was used to identify proteins in extracts, as previously described [17] except that a specific database derived from the *P*. *putida* LS46 proteome was used for alignment of peptides with the *P*. *putida* LS46 genome.

Protein and RNA Quantification: Transcripts per gene expressed were quantified by expressing the sum of all 100-mer RNA alignments per gene. Total ion counts (TIC), which are the sum of all CID (collision induced dissociation) fragment intensities of member peptides, were used for protein quantitation. Both values were transformed to a log2 scale for comparison and differential analysis. Two more filtering steps applied were: protein identifications were accepted only if at least two peptides were detected per gene and each peptide had expectation values of log(e) < - 1.5, and these were observed in both biological replicates; and RNAseq values were accepted only if there were at least two alignment counts per gene. Raw sequencing data and gene expression abundance value of the RNAseq analysis were deposited in NCBI Sequence Read Archive (SRA) through Gene Expression Omnibus (GEO) with accession number: GSE65029. The Gene expression abundance value of proteomics analysis was submitted as an online resource **(S1 Table)**.

#### ”Omic” data analysis

Detailed comparative analyses of the expression data gathered from waste glycerol versus the pure glycerol grown cells was conducted using an in-house ‘Omics data analysis system called “UNITY” [17]. The analysis operations in order were as follows: 1) The overall number of identified protein and RNA molecules were calculated: 2) The overall correlation between transcriptomic and proteomic log(2) expression values were observed; 3) Differential expression values were calculated: a) cross-state: Z = (Stationary phase of waste glycerol culture–Exponential phase of waste glycerol culture, or Exponential phase waste free fatty acid culture–Exponential phase waste glycerol culture); and b): among biological replicates, where R = intra biological replicate differences; 4) A quality control of biological variation signals between any two comparison groups (cross-state) vs system noise among biological replications (intra-replicative viability) were conducted; 5) Transformation of different populations into final Znet scores (Pnet for protein and Rnet for RNA) for differential expression analysis cross-state was conducted, as descripted in [18]. The cut-off scores of ± 1.65 were used to represent outermost 10% of the population defined as an asymmetry (up-regulated or down-regulated) of expression of RNA and protein relative to the expression profiles of the overall population; Thus, a gene having a final normalized Z score (e.g. Pnet for protein and Rnet for RNA) > 1.65 or < -1.65 was considered as significantly up- or down-regulated under the two specific conditions compared; 6) The substrate or condition specific RNA/Protein expression was then filtered by searching the Rnet/Pnet value representing the outermost 10% of each population (± 1.65) using COG categories for annotation [19] (**S2 Table**).

#### Data validation

Correlation of the RNA and Protein expression data under each tested experimental condition was done by plotting the Log2 expression values of transcriptomic and proteomic data (**S2 Fig**). The dynamic range of Log2 expression values was > 4 and > 11 for RNAseq and proteomic analyses, respectively. Furthermore, a simple function was developed to evaluate the statistical significance of a two-state two-replicate dataset, on a protein-by-protein basis, which computes an overall measurement of the system quality as the ratio of the mean of the standard deviations of the cross state and intra-replicate populations. This is known as the ‘system to noise’ (S/N) ratio. On an individual protein level, the S/N is the ratio of the vector magnitudes (protein expression levels) across the experimental states and intra-replicate normalized values, scaled by the overall system S/N values. A simple Monte-Carlo Model was used to derived functions relating ‘false discovery rates’ (FDR) to a defined S/N cutoff: all proteins with a S/N > 2.8 were found to have a FDR of 10% or less. This function allowed us to reliably access smaller differential expression values across experimental states, provided the variation of their corresponding intra-replicate measurements were sufficiently small.

#### Reverse transcription Quantitative PCR analysis

The transcriptional level of genes *pha*C1 and *pha*C2 were analyzed via RT-qPCR from the same culture as for ‘Omics analysis (above). Total RNA from three biological replicates of each sampling condition were isolated the same way as for RNAseq analysis. Standard curves using primer sets for *pha*C1 and *pha*C2 at final concentration of 0.2 μm and 0.3 μm, respectively, were amplified against various concentration of *P*. *putida* LS46 genomic DNA (200 ng, 20 ng, 2 ng, 0.2 ng. 0.02 ng) using SsoAdvanced™ universal SYBR® Green supermix (Bio-Rad, CA, USA). The amplification of the standard curves was initiated by denaturing at 98°C for 2 min, followed by 39 cycles of 98°C 15 s, 58°C 15 s for annealing/extention/plate read using CFX Connect Real-Time System (Bio-Rad, CA, USA). Next, 100ng total RNA were reverse transcripted into cDNA using the iScript reverse transcription supermix (Bio-Rad, CA, USA) under the condition: 25°C, 5 min; 42°C 30 min; 85°C 5 min. Subsequently, 10 ng of synthesized cDNA was directly amplified via qPCR as mentioned above along with the standard curves. For every gene under each condition, the average transcriptional level from biological triplicates samples were reported. Sequence of two primer sets:

*pha*C1 forward primer: 5’-CCGACCAATACCCTGTCCACCC-3’; *pha*C1 reverse primer: 5’-GCCGCCGTTATTGACCATGTCC-3’; *pha*C2 forward primer:5’-CCTGGCGCAGTGGTATTT-3’; *pha*C2 reverse: 5’-GCTGAGGTCGAAGATGTAGAAC-3’.

## Results

### Physiology of *P*. *putida* LS46 growing in WG and WFA

**Fig 1:**
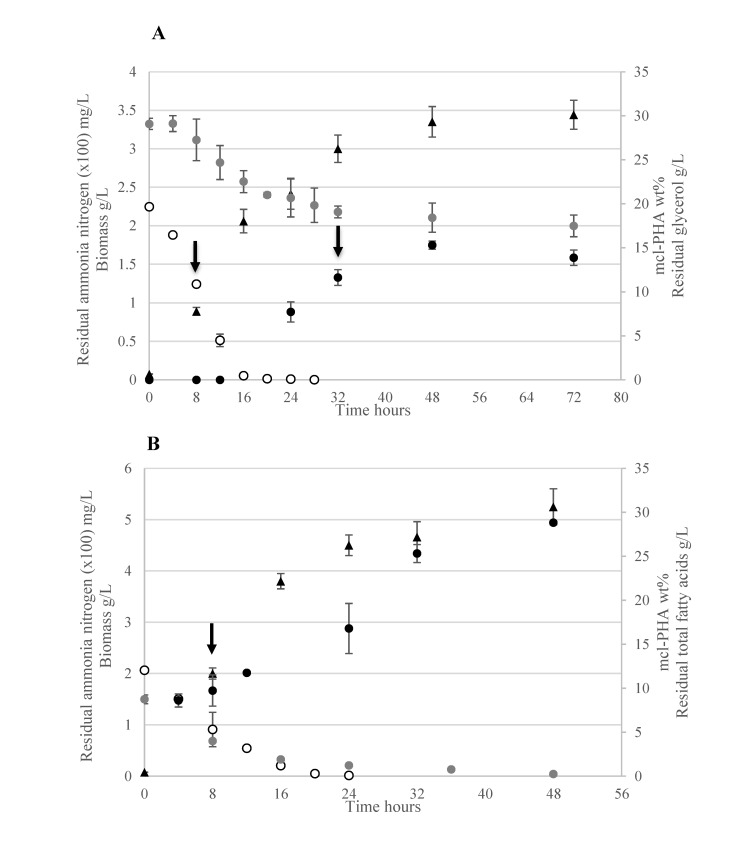
Growth of *P*. *putida* LS46 cultured with waste glycerol (WG) and waste fatty acids (WFA). *P*. *putida* LS46 was cultured in Ramsay’s Minimal Medium containing A) WG and B) WFA. Solid triangles, Biomass; Solid black circles, mcl-PHA synthesis; Solid gray circles, glycerol concentrations (A) and fatty acid concentration (B); Open circles, nitrogen concentrations. Error bars represent standard deviations about the means calculated from three independent, biological replicate experiments. Arrows indicate samples taken for subsequent RNA and Protein isolation.

Growth of *P*. *putida* LS46 in cultures containing biodiesel-derived glycerol (WG) and biodiesel-derived free fatty acids (WFA), and the points at which samples were taken (black arrows) for the ‘Omics analyses are indicated in **Fig 1**. The exponential growth phase of *P*. *putida* LS46 on WG was completed by 16 hours post inoculation (h pi) (**Fig 1A**), when nitrogen was mostly depleted, and active mcl-PHA synthesis began (**Fig 1A**). Consumption of glycerol slowed down during active polymer synthesis (after 16 h pi), and no further increase in mcl-PHA production was observed after 48 h pi, although glycerol was still in excess (**Fig 1A**). Thus, samples were taken for ‘Omics analyses at 8 h pi, representing the “non-permissive” condition for mcl-PHA synthesis, and at 32 h pi, representing the “permissive” condition for active mcl-PHA synthesis.

For mcl-PHA accumulation in WFA culture, it is worth to mention first that, the waste fatty acids inoculum contained about 13 wt% mcl-PHA. Thus, we observed a decrease in intra-cellular mcl-PHA to about 8.6 wt% by 4 h pi. The mcl-PHA content of the cells started to increase gradually up to 24 h pi, and then increased at a much greater rate after nitrogen concentrations in the medium were exhausted (**Fig 1B**). This suggested that mcl-PHA biosynthesis was not restricted to nitrogen-limitation under WFA culture, although the maximum productivity was triggered by nitrogen depletion. Therefore, samples were taken from WFA cultures only during the exponential growth, at 8 h pi, representing a different “permissive” condition for mcl-PHA synthesis.

The monomer composition of mcl-PHA polymers synthesized by *P*. *putida* LS46 cultured with WG and WFA varied primarily in the content of C8 and C10 monomers. C10 was the predominant monomer in polymers synthesized from WG cultures, while C8 was observed as the most abundant monomer in polymers synthesized from WFA cultures. According to our previous study, such monomer distribution profile of the mcl-PHA derived from two waste carbon sources was generally stable overtime [9]. Production of unsaturated mcl-PHA monomers was observed in both cultures. The polymer derived from WG cultures contained more C12:1 monomer, while polymers from WFA cultures contained greater mol % of monomers with longer chain length 3-hydroxyalkanoate monomers, such as C14 and C14:1 (**S3 Table**).

### Whole cell transcriptomics and proteomics analyses

RNAseq analysis identified approximately 5200 genes under each of three conditions, representing about 95% coverage of all protein coding genes in the *P*. *putida* LS46 genome. The 1D LC/MS label free proteomic analysis yielded an average number of 1362 final proteins from three tested conditions **(S4 Table)**. Thus, the depth of the transcriptome was much greater than the depth of the proteome.

The log(2) expression values of transcriptomic and proteomic data largely correlated with each other (with R^2^ ratio from about 0.24 to 0.42), suggesting that while the full depth of the differential analysis was best achieved with the transcriptomic data, the proteomic data could be used to support and confirm the transcriptomic data, when the proteins detected were sufficiently abundant. Note that the correlation was relatively low in WG cultures during stationary culture (average R^2^ ratio of 0.25), suggesting overall translation of gene transcripts (mRNA) into protein was more unstable under this condition, which could be expected under the assumption that higher protein degradation during the stationary stage of the culture comparing to exponentially growing cell results in higher degree of differences in transcriptome and Proteome correlation [20].

An average “signal to noise ratio” ratio of 3.48 was obtained for RNAseq data, suggesting the responses of *P*. *putida* LS46 to two mcl-PHA permissive conditions are profound at the transcriptional level, while the signal to noise ratio for the proteomic data was, on average, 1.55, indicating that the proteomic measurement system was not sufficiently sensitive to access changes in protein expression levels under the experimental conditions (**S5 Table**).

### Levels of expression of genes involved in mcl-PHA biosynthesis

Expression of mcl-PHA biosynthesis cluster genes, and several of previously identified monomer-supplying genes, are essential for the active mcl-PHA synthesis by *P*. *putida* LS46. Many of these genes were observed to be up-regulated, as shown in **Table 1**. However, expression levels of the genes appear to be linked to the specific growth conditions that are permissive for mcl-PHA synthesis. The data in support of this observation are: 1) The two tested mcl-PHA permissive conditions induced the overexpression of key mcl-PHA synthases (PhaC1 and PhaC2) at the protein level. However, while the transcriptional level of these genes (*phaC1* and *phaC2)* was similar during the mcl-PHA permissive condition in the WG culture (i.e. during the stationary phase relative to exponential phase), transcription of the *pha*C1 gene during the exponential phase of the WFA culture mcl-PHA were produced was elevated relative to exponential phase during growth on glycerol, where no detectable mcl-PHA production was observed. This key observation was confirmed using RT-qPCR. The qPCR results (**Table 2**) support the RNASeq data, and show that the transcription level of *pha*C1 was clearly higher in the WFA culture. Analysis of the qPCR data by T-test revealed that abundance of *pha*C1 transcripts in the WFA culture were significantly different from the abundance of *pha*C1 transcripts in the WG cultures (P = 0.04), whereas all the others were not significant from each other. The lack of correlation between mRNA and protein expression profiles of two mcl-PHA synthases suggests the potential for post-transcriptional regulation that is specific to the WG culture. 2) Specific expression patterns of several mcl-PHA monomer-supplying proteins (PhaG, PhaJ1, and PhaJ4) across growth phase and substrate were observed. Expression of the *pha*G gene was specific to WG cultures rather WFA cultures. However, surprisingly, we noticed that the *pha*J4 gene (which is usually proposed to be key protein for mcl-PHA biosynthesis from fatty acids [21]) was significantly up-regulated in the WG culture during the permissive mcl-PHA synthesis condition; 4) Two putative mcl-PHA granule associated protein coding genes, *pha*I and *pha*F, have the highest Pnet and Rnet values, indicating that these genes were most up-regulated in the cluster, under the conditions tested that favoured PHA production (WG-stationary phase and WFA-exponential phase). The enzymes encoded by these genes were also the most abundant proteins among all mcl-PHA biosynthesizing enzymes detected, suggesting their essential role for active mcl-PHA synthesis. Interestingly, unlike PhaF, the expression of PhaI was not detectable at mid-log WG culture (e.g. non-mcl-PHA producing condition).

**Table 1:** Expression values of key mcl-PHA biosynthesis genes under three experimental conditions ^a^.

				WG		WFA vs WG	
				(Sta vs Exp)		(Exp)	
Locus-tag (PPUTLS46_)	Gene symbol	Expression abundance ^b^		Differential expression ^c^		Differential expression ^c^	
		RNA	Protein	Rnet	Pnet	Rnet	Pnet
005596	*pha*I	10.1	- ^d^	**5.90**	+ ^e^	**6.61**	+
005601	*pha*F	12.7	21.5	**2.49**	**2.47**	**3.91**	**2.42**
005606	*pha*D	9.7	-	-0.53	-	0.07	-
005611	*pha*C2	11.3	17.0	-0.17	**2.53**	0.72	**2.05**
005616	*pha*Z	10.6	-	0.1	-	-	-
005621	*pha*C1	12.1	19.0	0.83	**2.04**	**1.94**	**1.74**
013888	*pha*G*	13.1	-	**2.09**	+	**-2.27**	-
021056	*pha*J1*	10.5	17.4	-	-	0.46	0.03
006576	*pha*J4	11.4	-	0.79	+	0.94	+

^a^ Exponential versus stationary growth phase in waste glycerol versus exponential growth phase in waste free fatty acids

^b^ Log2 expression abundance of gene products (mRNA and Protein) of *P*. *putida* LS46 grown under mid-log phase of waste glycerol culture (non-mcl-PHA synthesis condition). Values were averaged from two biological replicates

^c^ Differential expression values (see details in the Materials and Method section) of gene products under the specific comparing conditions: Sta, Stationary phase; Exp, Exponential phase; Significantly up and/or down-regulated proteins are indicated in bold

^d^ a “-” symbol means not detected mRNA and/or protein expression under mid-log phase of WG culture and/or two tested mcl-PHA synthesis condition(s)

^e^ a “+” symbol means protein expression of the gene was detectable under the specific mcl-PHA synthesis conditions: stationary culture of WG and/or exponential culture of WFA, respectively (e.g. protein expression abundance of PhaI: 22.9 and 23.5; PhaG: 17.1 under stationary culture of WG; PhaJ4: 19.1 and 18.9)

* symbol indicates proteins (if detected) with S/N (signal to noise) ratio less than 2.8, and thus having FDR greater than 10%.

**Table 2:** Transcription levels of the two *P*. *putida* LS46 PHA Synthases under three experimental conditions ^a^, as determined by RT-qPCR.

Locus-tag	Gene symbol	RT-qPCR ^b^		
(PPUTLS46_)		(cDNA ng x 10)		
		WG (Exp)	WG (Sta)	WFA (Exp) ^c^
005611	*pha*C2	0.19 ± 0.02	0.13 ± 0.06	0.20 ± 0.09
005621	*pha*C1	0.36 ± 0.09	0.49 ± 0.18	0.89 ± 0.15

^a^ Exponential versus stationary growth phase in waste glycerol versus exponential growth phase in waste free fatty acids

^b^ cDNA values of each gene from10 ng total cDNA input for RT-qPCR under three studied experimental conditions

^c^ Analysis of the qPCR data by a Two-tail T-test revealed that abundance of *pha*C1 transcripts in the WFA culture were significantly different from the abundance of *pha*C1 transcripts in theWG cultures (P = 0.04, where the threshold was P = 0.05), whereas all the others were not significant from each other.

### State-specific gene expression profile pertinent to mcl-PHA biosynthesis

The potential driving force behind the observed mcl-PHA production profiles, induced either by growth phase or substrate, was further studied by evaluating the gene expression profiles of the pathways feeding carbon to the mcl-PHA biosynthesis pathway, since these may further control the polymer content and monomer composition.

#### Waste glycerol derived gene expression variations during mcl-PHA biosynthesis

**Fig 2:**
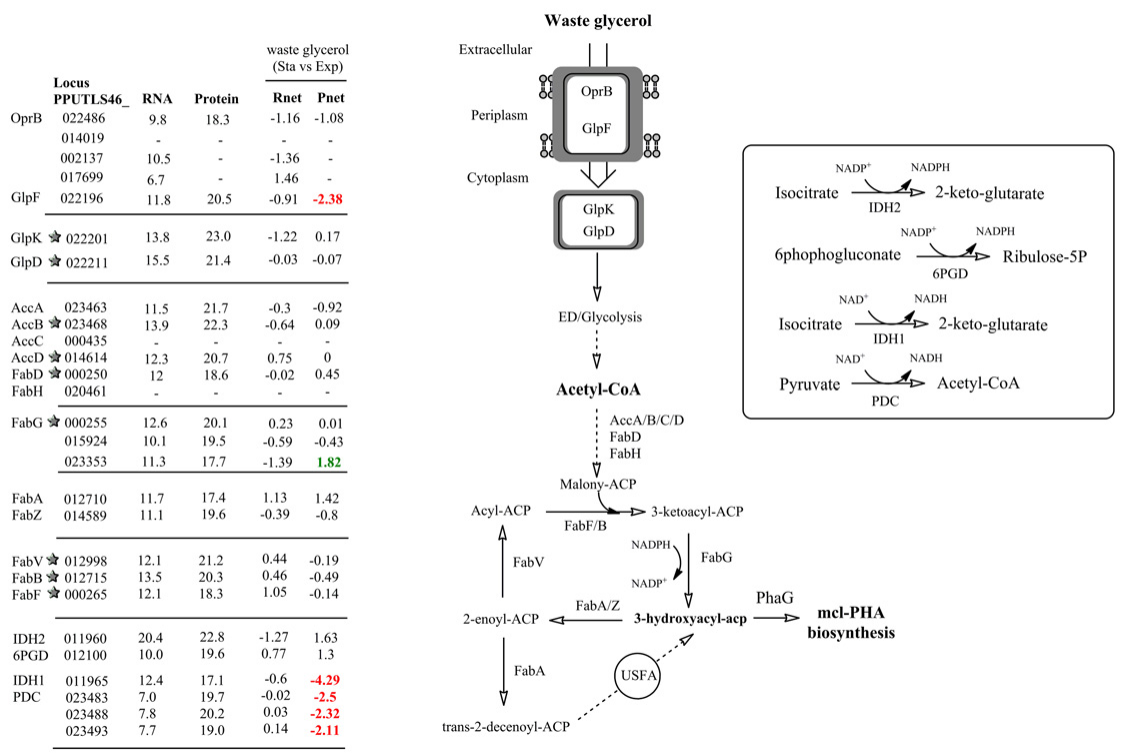
Expression values of genes and gene products involved in proposed mcl-PHA metabolism derived from waste glycerol (WG) culture of *P*. *putida* LS46. Numbers in each column (from left to right) represent: the gene locus tag; RNA abundance during Exponential phase of WG cultures; Protein abundance during Exponential phase of WG cultures; Rnet and Pnet values from WG cultures in Stationary phase versus Exponential phase; Significantly up-regulated mRNAs or proteins are indicated in green font; Significantly down-regulated mRNA or proteins are indicated in red font; -, not detected, and therefore no Rnet or Pnet value associated. A star symbol in front of gene locus tag indicates proteins (if detected) with S/N (signal to noise) ratio less than 2.8, and thus having FDR greater than 10%. USFA: unsaturated fatty acid. Gene symbols for each putative pathway protein were also given. IDH1: putative NAD^+^-dependent isocitrate dehydrogenase; IDH2: putative NADP^+^-dependent isocitrate dehydrogenase; PDC: Pyruvate dehydrogenase complex; 6PGD: 6-phosphogluconate dehydrogenase.

The substrate (glycerol) consumption rate of *P*. *putida* LS46 in WG cultures was low once the cells reached stationary phase, compared to the substrate (free fatty acids) consumption rate in WFA cultures, under active mcl-PHA biosynthesis conditions (**Fig 1A**). This was associated with significant reduction in protein expression of *glp*F gene, a transmembrane glycerol transportation facilitator, in the proteome of *P*. *putida* LS46 (**Fig 2**) suggesting the efficient transportation of extra-cellular glycerol into cytoplasm was restrained during stationary phase. While expression of other essential genes and homologs (i.e. *opr*B porin homologs) for glycerol transportation and catabolic initiation did not changed significantly.

Conversion of acetyl-CoA into 3-hydroxyacyl-ACP, a key precursor for mcl-PHA biosynthesis, was carried out via fatty acid *de novo* synthesis. The ‘Omics data identified gene products that putatively provide key pathway intermediates for polymer synthesis, and suggested an up-regulation on one of them during active mcl-PHA synthesis. (**Fig 2**). Protein levels of a putative ketoacyl-ACP reductase (FabG, encoded by PPUTLS46_023353), which is one of 8 homologs identified in the *P*. *putida* LS46 genome (**S6 Table**) that provide various 3-hydroxyacyl-ACP intermediates, was highly up-regulated in stationary phase of WG cultures when there was active mcl-PHA synthesis. RNAseq analysis, however, indicated down-regulation of this gene at the transcriptional level. Expression of two putative beta-hydroxyacyl-ACP dehydratases (FabA and FabZ) was detected, but with no significant changes in their expression level under the two comparing conditions of WG cultures. Two isoforms of beta-hydroxyacyl-ACP dehydratases identified in the genome of *P*.*putida* LS46 carry-out dehydration reactions to produce *trans*-2-acyl-ACP, and FabA also carries out an isomerization reaction leading to the unsaturated fatty acids biosynthesis [22], which could be a critical point for unsaturated mcl-PHA production from fatty acid *de novo* synthesis (**Fig 2**).

Overexpression of the gene for trehalose biosynthesis was observed during mcl-PHA synthesis in WG culture, and these genes are located in a gene cluster with putative glycogen metabolism genes (**Table 3**). Trehalose can be synthesized from maltose by Trehalose synthase (coded by *tre*S), or synthesized from maltodextrine by enzymes encoded by *tre*Z and *tre*Y [23,24]. Under current study Trehalose synthase (*tre*S, PPUTLS46_012155) has been up-regulated together with putative glycogen degrading enzymes coded by *mal*Q (PPUTLS46_012120) and *glg*P (PPUTLS46_005431), putatively offered the precursor for trehalose biosynthesis [25]. An additional Trehalose synthase coding gene (PPUTLS46_002907) was identified in *P*. *putida* LS46, however detected only at mRNA level (i.e. no protein was detected).

**Table 3:** Expression values of gene clusters putatively involved in trehalose biosynthesis and polyphosphate degradation in *P*. *putida* LS46 under mcl-PHA permissive vs non-permissive conditions in WG cultures.

					WG	
					(Sta vs Exp)	
Locus-tag	Gene symbol	Gene product	Expression abundance ^a^		Differential expression ^b^	
(PPUTLS46_)						
			RNA	Protein	Rnet	Pnet
012110	*glg*A	glycogen synthase	- ^c^	-	-	-
012115	*tre*Y	Malto-oligosyltrehalose trehalohydrolase	-	-	-	-
012120	*mal*Q	4-alpha-glucanotransferase	9.94	19.10	-0.07	**2.06**
012125	*tre*Z	maltooligosyl trehalose synthase	-	-	-	-
012130	*	protein of unknown function	8.98	20.04	-0.07	0.06
012135	*glg*X	glycogen debranching protein	-	-	-	-
012140		hypothetical protein	9.07	-	-0.44	-
012145		outer membrane autotransporter	7.34	-	0.99	-
012150	*glg*B*	glycogen branching enzyme	11.34	19.80	-0.42	1.21
012155	*tre*S	trehalose synthase	9.95	21.02	-0.59	**2.76**
012160		alpha amylase family protein	10.19	20.09	-0.9	1.28
002907	*tre*S	trehalose synthase (*Pseudomonas stutzeri* type)	7.66	-	0.11	-
005431	*glg*P	glycogen phosphorylase	11.14	22.36	-0.47	**2.45**

^a^ Log2 expression abundance of gene products (mRNA and Protein) of *P*. *putida* LS46 grown under mid-log phase of waste glycerol culture (non-mcl-PHA synthesis condition). Values were averaged from two biological replicates

^b^ Differential expression value of gene products under the specific comparing conditions: Sta, Stationary phase; Exp, Exponential phase; Significantly up-regulated proteins are indicated in bold

^c^ a “-” symbol means not detected mRNA and/or protein expression under mid-log phase of WG culture and stationary culture of WG culture

* symbol indicates proteins (if detected) with S/N (signal to noise) ratio less than 2.8, and thus having FDR greater than 10%.

Finally, ‘Omics analysis has revealed differential expression level (mostly at protein level) of genes putatively involved in recycling of cellular reducing equivalents, such as NADH and NADPH, under the mcl-PHA synthesizing stage (**Fig 2**).

#### Waste fatty acids derived gene expression variations during mcl-PHA biosynthesis

**Fig 3:**
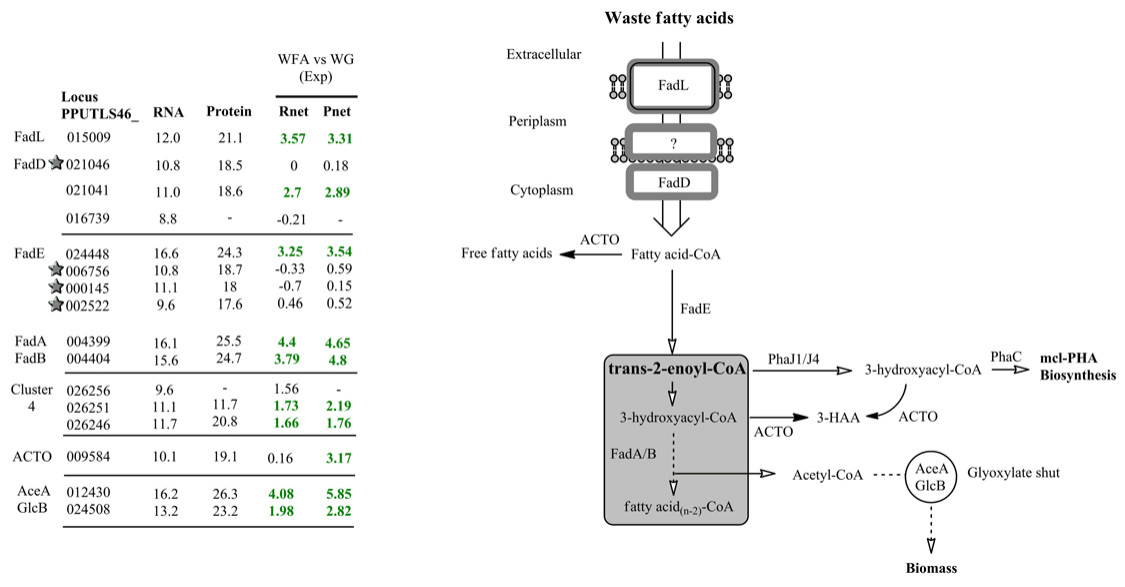
Expression values of genes and gene products involved in proposed mcl-PHA metabolism derived from waste fatty acids (WFA) culture of *P*. *putida* LS46. Numbers in each column (from left to right) represent: the gene locus tag; RNA abundance during Exponential phase of WFA cultures; Protein abundance during Exponential phase of WFA cultures; Rnet and Pnet values from the Exponential phase of WFA versus WG cultures; Significantly up-regulated mRNAs or proteins are indicated in green font; Significantly down-regulated mRNA or proteins are indicated in red font; -, not detected, and therefore no Rnet or Pnet value associated. A star symbol in front of gene locus tag indicates proteins (if detected) with S/N (signal to noise) ratio less than 2.8, and thus having FDR greater than 10%. ACTO: putative acyl-CoA thioesterase. Gene symbols for each putative pathway protein were also given. 3-HAA: 3-hydroxyalkanoic acid; AceA: isocitrate lyase; GlcB: malate synthase.

‘Omics analysis identified putative enzymes essential for fatty acids (the WFA used as substrate contained primarily long chain, C16 and C18, fatty acids) transport, activation and catabolism via fatty acid beta-oxidation, and with expression that were significantly up-regulated in WFA cultures, compared with the WG cultures, as expected (**Fig 3**).

*P*. *putida* encodes large numbers of fatty acid beta-oxidation enzymes that putatively carry out parallel biochemical reactions, and differential expression of these pathway genes was shown to potentially effect mcl-PHA synthesis as well as the monomer composition of the polymer [12,26]. Of the 18 identified *fad*E homologs identified in the *P*. *putida* LS46 genome, expression values of four FadE proteins were most abundant (**Fig 3 and S7 Table)**. The gene encoded by locus tag PUTLS46_024448 was the most significantly up-regulated gene in the WFA culture. The multifunctional fatty acid beta-oxidation complex, coded by the *fad*AB gene cluster (cluster 1), was highly up-regulated in the WFA culture. Furthermore, we found three more gene clusters in the genome of *P*. *putida* LS46 that are putatively involved in fatty acid oxidation (**S8 Table**), and cluster 4 genes were expressed at high levels, putatively under the regulation of the in-cluster TetR family regulator (PPUTILS46_026256) (**Fig 3**). The annotation of this gene cluster suggests putative roles in short chain fatty acid (≤ 4) degradation **(S8 Table**).

Finally, during the fatty acid oxidation, various CoA intermediates of the pathway are converted into their corresponding free acids by acyl-CoA thioesterase offering an optimal concentration of different pathway intermediates [27]. Expression of an acyl-CoA thioesterase-like protein (PPUTILS46_009584) was expressed at much higher levels in WFA cultures than in WG cultures (**Fig 3**). In addition, significant amount of acetyl-CoA from the fatty acid oxidation may flux to the “glyoxylate shunt”, as suggested by highly up-regulated isocitrate lyase and malate synthase of the pathway, which may serve as an important route for C2 assimilation to prevent carbon spillage in the form of CO_2_ [28].

## Discussion

Using biodiesel derived waste streams, such as waste glycerol and waste fatty acids, for mcl-PHA synthesis would potentially decrease the product cost, and therefore benefits the industrial biopolymer production. To study and predict the potential genetic targets that regulate mcl-PHA biosynthesis profile of *P*. *putida* LS46 grown under biodiesel derived waste carbon sources, Multi-level ‘Omics analyses were used to correlate the observed variations in mcl-PHA synthesis with differences in gene expression profiles with respect to: 1) mcl-PHA biosynthesis activation; 2) intracellular accumulation of mcl-PHA polymers; and 3) differences in monomer composition of the polymers.

### mcl-PHA biosynthesis activation machineries

Converting fatty acid metabolism pathway intermediates for mcl-PHA synthesis was dependent on two processes: monomer-supplying genes (*pha*G, *pha*J1, and *pha*J4) and the PHA synthesis cluster genes (*pha*C1/Z/C2/D, and *pha*I/F). Induction of mcl-PHA synthesis by nitrogen-limitation has been well reported in *P*. *putida* strains using structurally non-related carbon sources, such as glucose [29] and glycerol [9]. This, partially, due to induction of transcription of the key mcl-PHA monomer-supplying gene, *pha*G, by nitrogen depletion in *P*. *putida* LS46 WG cultures during stationary phase, which also observed in previous studies [12,30]. In contrast, during the exponential phase in WG culture, low transcription levels of the *pha*G gene, but PhaG protein, if produced, was produced at a level lower than the detection limit for our proteomic methods (**Table 1**). Note that the expression of PhaG protein was detectable in the stationary phase in WG culture with an expression abundance value at 17.1.

In addition to PhaG mediated induction of mcl-PHA biosynthesis, stationary phase WG cultures may also activate PhaJ4 driven polymer synthesis (**Table 1**). A possible mechanisms for this would be conversion of acyl-ACP from fatty acid biosynthesis into free fatty acids by thioesterase, which is used in PhaJ4 mediated polymer synthesis [31]. Homologs of *E*. *coli* type I and II thioesterase that hydrolyze both acyl-ACP and acyl-CoA [32] were identified in the *P*. *putida* LS46 genome (PPUTLS46_009584/006916/009634), however, with no significant changes in the levels of either transcription or protein expression in stationary versus exponential phases of WG cultures of *P*. *putida* LS46.

When grown on waste fatty acids, mcl-PHA biosynthesis was observed even in the presence of adequate nitrogen concentrations (**Fig 1B**), suggesting activation of mcl-PHA synthesis in cultures containing free fatty acids did not require strict nitrogen-limitation [33]. Rather, the mcl-PHA precursor was putatively synthesized under the expression of two proposed monomer-supplying proteins: PhaJ4 and PhaJ1 [21,34] observed in the WFA culture while nitrogen was still in excess (**Table 1 and Fig 3)**. Within the two proteins, expression of PhaJ4 was specifically observed under mcl-PHA biosynthesis under both WFA and WG culture suggesting the primary role in mcl-PHA biosynthesis derived from fatty acid beta-oxidation pathway [21].

In terms of the mcl-PHA biosynthesis gene cluster, up-regulation of the PHA synthase enzymes, PhaC1 and PhaC2, was observed for mcl-PHA biosynthesis under the two mcl-PHA synthesizing conditions: i.e. stationary phase of WG cultures and exponential phase of WFA culture (**Table 2**). However, expression of these two enzymes may be regulated by different mechanisms under the different growth specific conditions: 1) Although there were differences in the levels of PhaC1 and PhaC2 protein expression levels, no differences in the levels of *pha*C1 and *pha*C2 transcripts were observed between the stationary and exponential phases of WG cultures. These data suggest post-transcriptional regulation of PHA synthase enzyme expression in WG cultures (**Tables 2 and 3**).

It has been demonstrated that PhaC1 expression is repressed in *P*. *putida* KT2442 when cultured in media containing balanced carbon and nitrogen concentrations by regulatory mechanisms mediated by RsmA and Crc [35,36]. The Crc (Catabolite repression control) protein was shown to repress PhaC1 translation under balanced carbon and nitrogen conditions, during exponential growth in LB culture [36]. However, the Crc regulator was not detected under the conditions tested in the current work at either the mRNA or Protein level. RsmA (Regulator of secondary metabolites), another regulator of metabolism, belongs to CsrA (Carbon storage regulator) superfamily, and its role in regulation in a variety of bacterial species typically involves management of carbon storage and expression of virulence or biocontrol factors [37]. Recently, it has been suggested that the RsmA protein is involved in post-transcriptional regulation of the PhaC1 protein, putatively through a Gac/Rsm regulatory cascade [35]. Two CsrA homologs (PPUTLS46_020631 and PPUTLS46_015344) were detected in the transcriptome, but not in the proteome, of *P*. *putida* LS46, suggesting these regulators were likely expressed at low levels, and with no significant changes in expression levels across the current experimental conditions. RNA abundance of the two *csr*A homologs during the exponential growth phase in waste glycerol (WG) cultures were 7.69 and 6.04, respectively. The exact mechanism of this regulator on mcl-PHA biosynthesis is still unclear in *P*. *putida*. In contrast, higher levels of *pha*C1 transcripts and PhaC1 protein were observed in WFA cultures compared to WG cultures during exponential phase (**Tables 2 and 3)**. The in-cluster transcriptional activator, PhaD, likely activated *pha*C1 transcription when *P*. *putida* LS46 was cultured with waste free fatty acids [38].

A previous study of the PhaF protein has suggested that it has an essential role in mcl-PHA segregation during active cell division of *P*. *putida* KT2442 (grown on fatty acids), through binding polymer granules to the bacterial chromosome [39]. In our study, significant up-regulation of *pha*F transcripts and gene product (PhaF) was observed during mcl-PHA synthesis in WFA cultures during exponential phase and WG cultures during stationary phase. This suggested that detectable amount of mcl-PHA synthesized during stationary WG culture requires more phasin protein (e.g PhaF) for proper distribution of polymer into daughter cells, even though the cells may have a relatively low rate of cell division compared to cells in exponential growth phase in WFA cultures. The biological function of PhaI protein is still unknown. However, expression of the PhaI protein could only be detected during mcl-PHA synthesis (**Table 2**), suggesting expression of this protein was tightly regulated at the time when mcl-PHA polymers were not being actively synthesized under current experiment.

#### Molecular targets potentially affect the cellular mcl-PHA content

Attempts have been made to identify genetic targets that potentially affect mcl-PHA synthesis under the current experimental conditions (i.e. in the WG and WFA cultures). Reduced glycerol uptake rate was observed during active mcl-PHA synthesis in WG cultures **(Fig 1A**), which potentially counteracted with the efficient mcl-PHA production. Physiologically, limiting glycerol transport in the stationary phase of *P*. *putida* LS46 WG cultures would reduce the energy requirement for converting intra-cellular glycerol into glycerol-3-phosphate, and further metabolism [40], and repressed expression of the transmembrane glycerol uptake facilitator (GlpF) [41] may be the most efficient strategy for cells to suppress further glycerol transport (**Fig 2**). Reduced glycerol uptake, may have an effect on cell osmolality, as the high glycerol content of the culture medium could putatively create a hypertonic environment for *P*. *putida* LS46 cells. To counter this osmotic stress in WG cultures during active mcl-PHA synthesis, *P*. *putida* LS46 may specifically respond by up-regulating trehalose synthase (TreS) (**Table 3**), putatively increasing the intracellular concentration of trehalose [42,43]. Trehalose has been identified as protective agent under varies stress conditions, such as osmotic stress [24,44], thermoltolerance [45], desiccation [46] although the biosynthesis pathway, along with clustered putative glycogen synthesis pathway [47], potentially competes with mcl-PHA biosynthesis by diverting acetyl-CoA into gluconeogenesis.

During mcl-PHA synthesis via the fatty acid *de novo* synthesis pathway in WG cultures, overexpression of putative FabG (PPUTLS46_023353) potentially increased the cellular 3-hydroxyacyl-ACP pool, a key precursor for mcl-PHA biosynthesis (**Fig 2**). In addition, the reaction also couples with NADPH consumption, although some FabG homologs are also NADH-dependent [48]. Up-regulation of FabG protein may facilitate a higher proportion of reducing equivalents directed to fatty acids biosynthesis during the active mcl-PHA synthesis stage of *P*. *putida* LS46 grown on waste glycerol. Simultaneously, *P*. *putida* LS46 up-regulated enzymatic reactions putatively to replenish the cellular NADPH pool via NADP-dependent isocitrate dehydrogenase (PPUTLS46_011960) [49] and 6-phosphogluconate dehydrogenase (PPUTLS46_012100), whereas expression of numerous tricarboxylic acid (TCA) cycle enzymes that supply bulk of NADH, such as putative NAD^+^-dependent isocitrate dehydrogenase and pyruvate dehydrogenase, were significantly down-regulated during the mcl-PHA synthesis stage in WG cultures (**Fig 2**). A similar pattern of expression for these enzymes was observed in *P*. *fluorescence* under oxidative stress [50], suggesting their essential roles in balancing cellular reducing equivalents (i.e. level of NADH/NADPH). Elevated intracellular ratios of NADH/NAD^+^ and NADPH/NADP^+^ are known to be important for PHA biosynthesis in many Gram-negative bacteria [51–54]. Overexpression of pyruvate dehydrogenase subunit (*aco*A) was shown to have positive effect on maximizing mcl-PHA production of *P*.*putida* KT2440 grown on glucose [2], which putatively increased the acetyl-CoA and NADH pool used in fatty acid biosynthesis during mcl-PHA biosynthesis stage.

*P*. *putida* LS46 growth on waste fatty acids was maintained simultaneously with synthesis of mcl-PHA (**Fig 1B**), which corresponds with the high levels of expression observed for mcl-PHA biosynthesis and glyoxylate shunt enzymes (**Fig 3**). Elevated levels of the glyoxalate shunt have also been observed in *Escherichia coli* during growth on fatty acids [28]. The two pathways may thus prevent large amounts of carbon being oxidized through the TCA cycle by directing the carbon to biomass production and polymer synthesis in waste fatty acid cultures. As for mcl-PHA biosynthesis, production of the key precursor pathway trans-2-enoyl-CoA is putatively provided by acyl-CoA dehydrogenase (FadE, PPUTLS46_024448) (**Fig 3**). Expression level of this enzyme were found to be a limiting factor for PHA synthesis by recombinant *E*. *coli* [55].

The highly expressed putative acyl-CoA thioesterase (PPUTLS46_009584) has a 23% amino acid sequence identity with the well-studied *E*. *coli* type II acyl-CoA thioesterase (encoded by *tes*B) and a 40% amino acid identity to the *tes*B-like 3-hydroxyacyl-CoA specific thioesterase in a PHA producing, hydrocarbon-degrading, Gram-negative bacterium, *Alcanivorax borkumensis* SK2 [56]. However, the effect of *P*. *putida* thioesterase expression on mcl-PHA synthesis has not been studied in *P*. *putida* strains. The expression of acyl-CoA thioesterase may be strongly associated with the fatty acid metabolism in term of mediating the amount of the pathway intermediates for optimized fatty acid oxidation [27], and in some case, resulted in phenotypic benefit for cell grown on hydrocarbon [57, 58]. Such that this enzyme represents a “branch-point” allowing either the storage of the metabolic precursors in the form of PHA or the ability to hydrolyze fatty acid CoA esters.

#### Factors potentially determining the mcl-PHA monomer composition

Although PhaC1 and PhaC2 are the enzymes that carry-out PHA polymerization, the composition of the polymer may be also determined by several monomer-supplying enzymes that convert fatty acids to various 3-hydroxyacyl-CoA precursors which are then utilized by the PHA synthases. The monomer-supplying enzymes were differentially expressed under the two mcl-PHA production conditions used in this study. The PhaG enzyme has been associated with a preference for 3-hydroxydecanoyl-ACP in *P*. *putida* KT2440 [59,60]. High levels of PhaG expression were also observed in *P*. *putida* LS46 cultured with WG, which synthesized mcl-PHAs that contained a high mol % of 3-hydroxydecanoic acid (C10) subunits. Mcl-PHA polymers synthesized by *P*. *putida* LS46 cultured with WFA contained a high mol% of 3-hydroxyoctanoic acid (C8) subunits. PhaJ1 has been shown to synthesize R-3-hydroxyoctanoyl-CoA [21]. Considering the fact that PhaJ4 was expressed under both mcl-PHA permissive conditions, specific expression of PhaJ1 under mcl-PHA synthesis condition in *P*. *putida* LS46 cultured with WFA may contribute to the increased mol % of C8 monomers in the polymers synthesized.

In addition to the monomer-supplying proteins synthesized by *P*. *putida* LS46 in the presence of free fatty acids, this strain also encodes and expresses a large number of putative “redundant” fatty acid oxidation genes that carry out parallel reactions, and whose expression may vary under different growth condition [12,26]. FadE carries out the dehydration of acyl-CoA into trans-enoyl-CoA, an important intermediate for mcl-PHA synthesis **(Fig 3**). Except for its potential role in mcl-PHA production as mentioned above, effect of expression of FadE (as well as other fatty acid oxidation enzymes) on mcl-PHA monomer composition is largely unknown, due to large number of isozymes with limited data on enzymatic activity analysis or knockout experiments [61]. In the current study, PPUTLS46_024448 (one of a number of FadE homologs in *P*. *putida* LS46) and Cluster 1 fatty acid oxidation enzymes (**Fig 3**) were up-regulated in WFA cultures, and thus may play a primary role in synthesis of acyl-CoA intermediates. Orthologs of these enzymes were shown to be induced by long chain fatty acids (≥ C14) in *P*. *putida* KT2440 and *P*. *aeruginosa* PAO1 [62,63], which agrees with the fact that long chain fatty acids are the primary substrate in WFA cultures in the current study. Non-detected Cluster 2 and 3 proteins (some of these clusters genes were identified in RNAseq, however at much lower transcription level compare to Cluster 1 genes, **S8 Table**) in *P*. *putida* LS46 cultured with WFA likely serve as alternative fatty acid oxidation enzymes induced when expression of major fatty acid oxidation enzymes (Cluster 1) were not favoured or been knockout out [63]. Overexpression of Cluster 3 proteins led to a higher proportion of C10 monomers in mcl-PHA polymers synthesized by *P*. *putida* KT2440 grown on decanoic acid [12], suggesting expression of different fatty acid oxidation enzymes effect the polymer composition.

Finally, in the current study, we have suggested a possible correlation between expression of the FabA protein (**Fig 2**) and synthesis of mcl-PHAs with unsaturated subunits in *P*. *putida* LS46 cultured with WG during active PHA synthesis (i.e. stationary phase). The FabA enzyme has been shown to carry-out an essential step in unsaturated fatty acid biosynthesis from the fatty acid *de novo* synthesis in *P*. *aeruginosa* PAO1 [22], and forms cis-3-decenoyl-ACP [64], which is then converted to 3-hydroxy-cis-5-dodecanoyl-ACP (C12:1), which may be used in the synthesis of unsaturated mcl-PHA subunits. The monomer profile observed in mcl-PHAs synthesized by *P*. *putida* LS46 cultured with WG (**S3 Table**) is consistent with this process. This mechanism may be more generally applied to other mcl-PHA structurally non-related carbon sources, such as glucose [29].

## Conclusion

Quantitative ‘Omics analysis of *P*. *putida* LS46 was used to correlate variations in gene and gene product expression profiles with mcl-PHA biosynthesis in cells using “waste” biodiesel-derived glycerol and biodiesel-derived free fatty acids as the carbon sources. Synthesis of mcl-PHA polymers was influenced not only with the expression of PHA synthesis cluster gene products, but also by a complex regulatory machinery that induces expression of both monomer-supplying proteins and fatty acid metabolism enzymes, whose expression levels enhanced under different growth conditions. The results also suggest that optimized carbon flux to polymer synthesis by *P*. *putida* LS46 when grown on waste glycerol or waste fatty acids was potentially affected by the pathways (enzymatic relations) involved in substrate transport, providing or diverting key mcl-PHA biosynthesis pathway precursor molecules (such as, acetyl-CoA and 3-hydroxyacyl-acp/CoA), and cellular reducing equivalents. Furthermore, the ‘Omics data from WG or WFA cultures suggested variations in expression levels of specific mcl-PHA monomer-supplying proteins (i.e. PhaJ1 and PhaG) during active mcl-PHA synthesis conditions, which may play an essential role in determining distribution of dominant mcl-PHA monomers (i.e. C8 and C10) under these specific culture conditions.

## Supporting Information

S1 Fig SDS-PAGE of total protein extracted from *P*. *putida* LS46 grown under three experimental conditions. (PDF)

S2 Fig Correlation of log(2) expression values of transcriptomic and proteomic data from two biological replicates of *P*. *putida* LS46 grown under three experimental conditions. (PDF)

S1 Table Proteomic analysis of *P*. *putida* LS46 gene expression value under three experimental conditions. (PDF)

S2 Table Numbers of significantly up- and down-regulated genes and gene products in specific COG groups under the specific growth conditions. (PDF)

S3 Table Monomer composition of mcl-PHA synthesized by *P*. *putida* LS46 grown on biodiesel- derived waste glycerol (WG) and waste fatty acid (WFA) cultures. (PDF)

S4 Table Summary of RNA (Rnet) and protein (Pnet) scores from the RNAseq and 1D proteomic analyses under three experimental conditions. (PDF)

S5 Table Overall biological signal to systematic noise ratio of RNAseq and Proteomic analyses under three experimental conditions. (PDF)

S6 Table Variation in expression values of putative fatty acid *de novo* synthesis genes and gene products in of *P*. *putida* LS46 under two growth conditions. (PDF)

S7 Table Expression values of putative FadE homologs in *P*. *putida* LS46 grown in waste fatty acids (WFA) cultures during exponential phase, and variations in their expression levels under two conditions. (PDF)

S8 Table Expression values of putative fatty acid beta-oxidation genes and gene products in *P*. *putida* LS46 grown in waste fatty acids (WFA) cultures during exponential phase, and variations in their expression levels under two conditions. (PDF)
