# Supplementary material for: Quantitative ‘Omics Analyses of Medium Chain Length Polyhydroxyalkanaote Metabolism in Pseudomonas putida LS46 Cultured with Waste Glycerol and Waste Fatty Acids
Source: PLoS One. 2015 Nov 6;10(11):e0142322. doi: 10.1371/journal.pone.0142322 (PMC4636370; doi:10.1371/journal.pone.0142322)
Supplement: S1 Fig — (PDF) [file pone.0142322.s001.pdf]

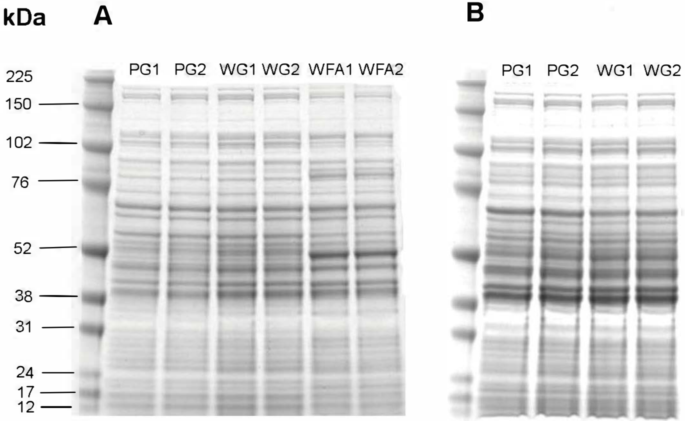

S1. Fig: SOS-PAGE of total protein of *Pputida* LS46 grown under exponential phase (A) of waste glycerol and waste fatty acids culture, and stationary phase (B) of waste glycerol culture.
WG: waste glycerol; WFA: waste fatty acids; Numbers indicate two biological replicate.
Protein samples from Pure glycerol (PG) culture under exponential and stationary phase were ran as reference.
