# Supplementary material for: Quantitative ‘Omics Analyses of Medium Chain Length Polyhydroxyalkanaote Metabolism in Pseudomonas putida LS46 Cultured with Waste Glycerol and Waste Fatty Acids
Source: PLoS One. 2015 Nov 6;10(11):e0142322. doi: 10.1371/journal.pone.0142322 (PMC4636370; doi:10.1371/journal.pone.0142322)
Supplement: S2 Fig — (PDF) [file pone.0142322.s002.pdf]

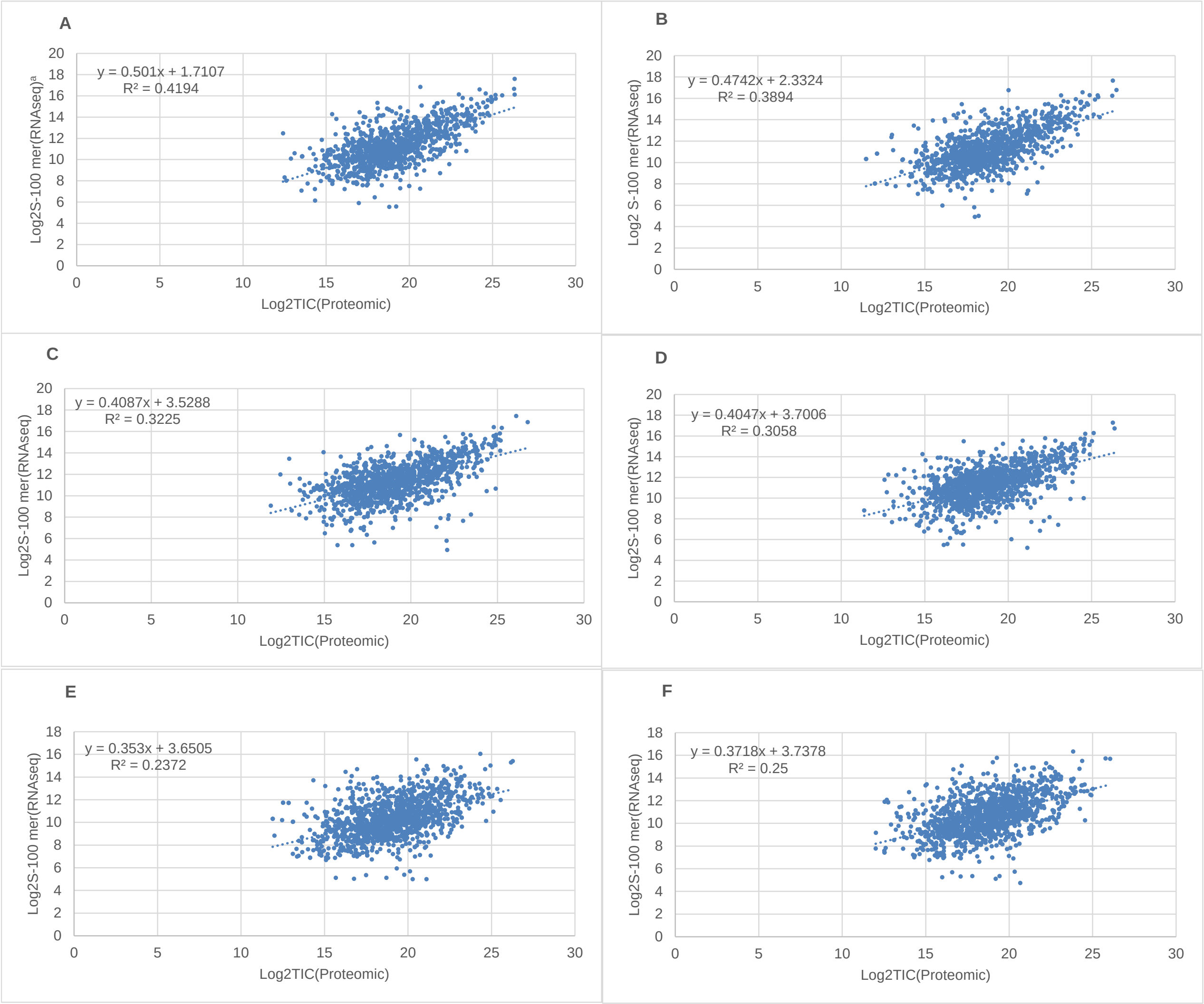

S2. Fig: Correlation of log(2) expression values of transcriptomic and proteomic of two biological replicates under the three experimental conditions.

[a]: Sum of total 100-mer alignment per gene; Panel A/B: Waste fatty acids exponential phase culture biological replicate 1 and 2; Panel C/D: Waste glycerol exponential phase culture biological replicate 1 and 2; Panel E/F: Waste glycerol stationary phase culture biological replicate 1 and 2.
