# Supplementary material for: Quantitative ‘Omics Analyses of Medium Chain Length Polyhydroxyalkanaote Metabolism in Pseudomonas putida LS46 Cultured with Waste Glycerol and Waste Fatty Acids
Source: PLoS One. 2015 Nov 6;10(11):e0142322. doi: 10.1371/journal.pone.0142322 (PMC4636370; doi:10.1371/journal.pone.0142322)
Supplement: S1 Table — (PDF) [file pone.0142322.s003.pdf]

S1 Table: Proteomics analysis of *P.putida* LS46 gene expression value under three studied experimental conditions.

a: Log2TIC value of proteins (if detected). Values from two biological replicates derived from three experimental conditions (see method part of the paper) were given.
b: A positive Pnet value indicated the up-regulation of the protein under the specific set of comparing conditions (see below 1/2), whereas negetive Pnet value indicates the down-regulation of the protein. Whereever no Pnet value associated for a gene, means the protein was not detected in one or both comparing conditions.
c: Singal to Noise ratios of individual protein under two comparing conditions: proteins with a S/N value 2.8 or greater having a false discovery rate (FDR) 10% or less.
1: Differential protein expression values between waste glycerol stationary culture vs exponential culture
2: Differential protein expression value between waste fatty acid exponential culture vs waste glycerol exponential culture

	Gene Annotation	Protein Expression Abundant [a]						Differential Expression [b]		Signal to Noise ratio [c]	
		WG_Exp1	WG_Exp2	WG_Sta1	WG_Sta2	WFA_Exp1	WFA_Exp2	P1net	P2net	S/N 1	S/N 2
PPUTLS46_024028	translation elongation factor 1A (EF-1A/EF-Tu)	26.74	26.36	26.27	25.77	26.29	26.48	-0.39	0.09	4.21	0.73
PPUTLS46_004174	OmpF family protein	26.08	26.26	26.16	26.04	26.32	26.26	-0.14	0.08	0.55	1.15
PPUTLS46_024033	translation elongation factor 2 (EF-2/EF-G)	25.24	25.11	24.94	24.36	25.17	25.35	-0.31	0.08	2.4	1.19
PPUTLS46_019606	succinyl-CoA synthetase subunit beta	25.18	24.87	24.46	24.33	24.8	24.72	-0.45	-0.01	3.96	0.39
PPUTLS46_020456	flagellin domain-containing protein	25.16	24.88	21.49	20.91	24.66	24.55	-3.51	-0.06	18.22	2.43
PPUTLS46_016679	F0F1 ATP synthase subunit beta	25.13	24.57	25.55	24.86	24.96	24.37	0.06	0.21	1.11	0.45
PPUTLS46_013648	chaperonin GroEL	25.08	24.5	24.5	24.29	24.4	24.06	-0.25	-0.06	2.83	1.47
PPUTLS46_022581	arginine deiminase	24.91	24.33	25.34	25.01	24.96	24.31	0.42	0.1	4.13	0.81
PPUTLS46_022586	ornithine carbamoyltransferase	24.9	24.56	24.84	24.92	24.43	24.78	0.16	0.13	0.62	0.71
PPUTLS46_019281	Quinoprotein ethanol dehydrogenase	24.89	24.51	24.67	24.54	15.64	12.72	0.02	-4.99	0.95	3.71
PPUTLS46_022151	anti-oxidant AhpCTSA family protein	24.88	24.55	24.06	23.69	24.68	25.48	-0.77	0.11	18.29	1.12
PPUTLS46_011220	ethanol-active dehydrogenase/acetaldehyde-active	24.86	23.98	24.27	23.75	23.37	24	-0.08	-0.08	1.35	1.17
PPUTLS46_024003	50S ribosomal protein L2	24.81	23.73	23.37	22.75	24.18	23.52	-0.71	-0.38	2.52	0.29
PPUTLS46_015469	30S ribosomal protein S1	24.78	24.61	24.33	23.83	24.58	24.87	-0.45	0.06	3.63	0.9
PPUTLS46_016669	F0F1 ATP synthase subunit alpha	24.77	25.01	25.04	24.68	25.18	25.19	-0.01	0.18	0.89	1.37
PPUTLS46_007101	molecular chaperone DnaK	24.7	24.5	23.78	23.98	23.79	24.06	-0.29	-0.07	1.74	1.32
PPUTLS46_014539	translation elongation factor Ts (EF-Ts)	24.64	23.8	23.7	23.71	24.25	23.5	-0.23	-0.36	1.68	0.23
PPUTLS46_024068	50S ribosomal protein L1	24.63	23.7	22.95	22.65	23.94	23.85	-1.02	-0.07	4.12	0.73
PPUTLS46_024053	DNA-directed RNA polymerase subunit beta	24.48	23.98	24	23.85	23.63	23.35	-0.16	-0.29	2.17	3.49
PPUTLS46_007356	ketol-acid reductoisomerase	24.41	23.85	23.51	23.08	23.96	23.69	-0.68	-0.07	6.15	0.33
PPUTLS46_019251	aldehyde dehydrogenase	24.37	23.72	22.94	22.69	21.84	21.75	-1.03	-1.64	7.03	10.06
PPUTLS46_024048	DNA-directed RNA polymerase subunit beta'	24.28	23.87	23.71	23.82	23.9	23.14	-0.01	0.02	1.13	0.99
PPUTLS46_024018	50S ribosomal protein L3	24.21	24	22.74	22.4	23.72	23.96	-1.39	-0.16	14.45	0.31
PPUTLS46_021586	porin	24.15	24.1	24.35	24.51	23.54	23.49	0.2	-0.11	0.62	1.63
PPUTLS46_019601	succinyl-CoA synthetase subunit alpha	24.1	22.61	23.42	22.57	23.44	22.62	-0.29	-0.63	0.6	0.23
PPUTLS46_005216	protein MaeB	24.09	23.85	21.91	21.98	22.29	22.11	-1.65	-1.3	6.15	15.49
PPUTLS46_014194	50S ribosomal protein L19	24.07	23.45	22.54	22.19	23.59	23.49	-1.21	0.07	9.58	0.76
PPUTLS46_009559	histone family protein DNA-binding protein	24.06	21.65	25.11	24.23	22.23	20.51	0.58	-0.51	1.42	0.68
PPUTLS46_021686	outer membrane protein H1	23.96	23.77	23.82	23.59	24.93	24.33	-0.02	0.69	1.35	3
PPUTLS46_023938	50S ribosomal protein L18	23.91	23.29	22.32	22.99	23.96	22.57	-0.29	-0.33	1.4	0.58
PPUTLS46_009539	trigger factor	23.88	24.07	23.17	22.94	23.7	23.74	-0.56	-0.16	2.66	0.51
PPUTLS46_000260	acyl carrier protein	23.86	23.53	20.66	17.63	23.28	23.51	-2.47	-0.08	2.17	0.55
PPUTLS46_019616	dihydrolipoamide succinyltransferase	23.85	24.01	23.05	23.29	23.81	23.15	-0.19	-0.03	1.38	1.12
PPUTLS46_021896	leucine ABC transporter subunit	23.8	23.34	24.92	24.48	21.98	21.91	1	-1.17	11.03	16.61
PPUTLS46_010554	cold shock protein CspA	23.79	23.85	22.49	18.97	23.97	24.16	-0.93	0.14	1.38	1.32
PPUTLS46_022201	glycerol kinase	23.78	22.27	23.74	23.17	22.19	21.45	0.19	-0.03	0.79	1.08
PPUTLS46_023898	30S ribosomal protein S4	23.77	23.28	22.97	22.28	23.54	23.63	-0.59	0.19	3.16	1.92
PPUTLS46_024058	50S ribosomal protein L7/L12	23.76	23.3	23.26	23.4	23.5	23.24	0	0.03	0.91	0.97
PPUTLS46_016889	pyruvate carboxylase subunit B	23.75	22.86	21.93	21.43	22.46	22.6	-1.26	-0.18	4.84	1.53
PPUTLS46_006281	azurin	23.73	24	24.47	23.44	23.02	23.73	0.03	-0.45	0.99	0.33
PPUTLS46_024038	30S ribosomal protein S7	23.69	23.13	23.2	22.77	23.47	23.1	-0.27	0.1	3.24	0.63
PPUTLS46_023923	50S ribosomal protein L15	23.68	23.93	22.01	22.76	23.26	23.5	-0.47	-0.24	1.53	0.44
PPUTLS46_019431	universal stress protein	23.67	22.86	23	22.8	21.9	21.17	-0.16	-0.86	1.65	2.83
PPUTLS46_019611	dihydrolipoamide dehydrogenase	23.66	23.41	23.71	23.5	23.51	23.09	0.1	0.03	0.35	1
PPUTLS46_024598	pyruvate dehydrogenase subunit E1	23.66	23.56	24.39	23.94	21.78	22.14	0.39	-0.89	2.84	3.42
PPUTLS46_022536	glyceraldehyde-3-phosphate dehydrogenase	23.65	22.73	20.54	19.49	16.01	16.87	-2.43	-5.29	4.73	8.03
PPUTLS46_013643	co-chaperonin GroES	23.61	23.4	22.06	22.21	23.42	22.89	-0.95	0.03	3.47	1.01
PPUTLS46_023533	aldehyde dehydrogenase family protein	23.56	23.21	23.07	22.67	23.44	22.59	-0.45	-0.06	9.52	0.81
PPUTLS46_023943	50S ribosomal protein L6	23.51	23.17	22.12	22.21	24.07	23.16	-0.82	0.14	3.53	1.21
PPUTLS46_023488	transketolase, central region	23.46	22.47	20.4	20.06			-2.3		7.36	12.51
PPUTLS46_023893	DNA-directed RNA polymerase subunit alpha	23.44	22.82	22.86	22.1	21.69	22.4	-0.27	-0.29	1.81	1.54
PPUTLS46_006246	50S ribosomal protein L9	23.37	23.04	22.39	21.58	22.82	22.78	-0.83	-0.04	3.25	1.94
PPUTLS46_009734	bifunctional aconitate hydratase	23.34	22.92	23.39	22.82	24.42	24.26	-0.04	1.36	0.63	37.65
PPUTLS46_022531	phosphogluconate dehydratase	23.34	22.73	21.69	21.02	17.97	17.75	-1.32	-4.39	5.48	27.93
PPUTLS46_018079	putrescine ABC transporter, periplasmic	23.32	23.13	24.16	23.49	22.78	22.75	0.36	-0.03	2.13	1.43
PPUTLS46_001662	ATP-dependent Clp protease, ATP-binding subunit	23.25	22.86	22.49	22.34	22.54	22.22	-0.49	-0.26	4.57	3.31
PPUTLS46_019691	electron transfer flavoprotein, beta subunit	23.23	23.34	22.42	22.43	24.7	25.11	-0.44	1.29	2.17	3.62
PPUTLS46_023933	30S ribosomal protein S5	23.17	23.16	22.88	21.86	23.47	22.27	-0.33	-0.03	1.63	0.91
PPUTLS46_023758	OmpW family protein	23.16	23.85	22.95	22.83	23.14	23.44	-0.05	0.49	1.12	0.4
PPUTLS46_014534	30S ribosomal protein S2	23.16	22.77	22.37	22.43	22.02	22.19	-0.27	-0.34	1.98	2.74
PPUTLS46_009649	Protein of unknown function (DUF3359)	23.15	23.22	23.17	23.1	23.2	23.57	-0.19	0.05	0.37	0.96
PPUTLS46_013658	pyruvate kinase	23.15	23.21	23.2	23.09	20.04	20.5	-0.16	-1.95	0.41	5.07
PPUTLS46_024063	50S ribosomal protein L10	23.08	21.66	22.09	22.47	22.03	20.17	0.03	-0.3	1	0.75
PPUTLS46_024603	dihydrolipoamide acetyltransferase	23.08	22.79	23.66	23.2	21.41	20.75	0.39	-1.15	3.97	4.39
PPUTLS46_019621	2-oxoglutarate dehydrogenase E1 component	23.04	22.17	23.23	23.08	22.75	22.67	0.29	0.12	1.79	1.27
PPUTLS46_007181	bacterial translation initiation factor 2	23.04	22.13	22.56	21.99	21.58	21.72	0.01	-0.29	0.95	1.81
PPUTLS46_024308	PrkA family serine protein kinase	23.01	22.2	22.93	22.51	23.17	22.84	0.12	0.33	0.64	1.95
PPUTLS46_024073	50S ribosomal protein L11	23.01	22.87	21.88	21.34	22.5	22.55	-1.09	-0.02	6.15	0.77
PPUTLS46_023483	pyruvate dehydrogenase	23.01	22.99	19.36	19.98			-2.48		4.22	22.25
PPUTLS46_019631	succinate dehydrogenase flavoprotein subunit	22.99	22.06	22.86	22.59	22.7	22.82	0.04	0.22	0.96	1.46
PPUTLS46_025193	outer membrane porin	22.98	23.62	21.8	22.6	22.36	22.98	0.02	-0.43	1.01	0.45

PPUTLS46_004464	glyceraldehyde-3-phosphate dehydrogenase	22.97	22.09	22.19	21.63	22.88	22.96	-0.26	0.35	1.82	1.86
PPUTLS46_023958	50S ribosomal protein L5	22.97	23.02	22.45	22	22.29	22.61	-0.51	-0.12	2.95	0.64
PPUTLS46_022221	amino acid ABC transporter substrate-binding	22.94	23.28	24.15	23.57	22.32	22.37	0.41	-0.03	1.78	1.13
PPUTLS46_011965	isocitrate dehydrogenase	22.89	22.35	16.54	17.68	21.57	21.29	-4.27	-0.69	4.69	5.86
PPUTLS46_005411	glutamine synthetase	22.88	22.53	24.81	24.38	22.97	23.16	1.76	0.4	22.26	2.64
PPUTLS46_017904	thioredoxin	22.88	22.73	22.37	22.05	22.79	22.62	-0.45	0.09	4.46	1.39
PPUTLS46_016659	F0F1 ATP synthase subunit B	22.88	22.55	23	22.91	21.45	22.5	0.05	-0.09	1.13	0.85
PPUTLS46_024903	extracellular solute-binding protein	22.87	22.27	23.13	22.99	22.83	22.42	0.3	0.09	2.38	1.23
PPUTLS46_006231	30S ribosomal protein S6	22.86	22.59	22.57	21.86	22.35	22.04	-0.24	-0.14	1.87	2.24
PPUTLS46_023993	50S ribosomal protein L22	22.85	19.06	22.28	21.9	21.81	18.89	0.18	-2.45	0.93	0.15
PPUTLS46_023908	30S ribosomal protein S13	22.84	20.41	22.08	20.26	22.25	20.66	-1.15	1.41	0.31	0.17
PPUTLS46_001452	50S ribosomal protein L21	22.8	22.92	20.28	20.6	22.52	22.24	-1.75	-0.02	4.01	1.14
PPUTLS46_009009	putative glucose dehydrogenase subunit alpha	22.79	22.38	22.51	22.43			0.02		0.95	174.44
PPUTLS46_023968	50S ribosomal protein L14	22.78	22.69	21.99	21.51	22.84	22.23	-0.77	0.04	4.75	1.03
PPUTLS46_024013	50S ribosomal protein L4	22.78	22.46	21.65	22.52	22.37	22	-0.14	-0.04	0.82	1.44
PPUTLS46_021556	Ferritin Dps family protein	22.77	23.26	22.27	22.6	22.51	23.27	-0.1	0.72	0.81	0.15
PPUTLS46_023903	30S ribosomal protein S11	22.75	22.88	21.36	21.86	22.87	22.75	-0.46	0.04	1.67	1.02
PPUTLS46_001262	50S ribosomal protein L25/general stress protein	22.74	22.62	22.54	22.19	21.88	22.45	-0.18	-0.16	2.34	0.62
PPUTLS46_005011	dihydroxy-acid dehydratase	22.73	22.61	21.19	21.73	21.58	21.73	-0.51	-0.43	1.76	2.79
PPUTLS46_013423	50S ribosomal protein L13	22.72	20.41	22.31	21.46	21.49	18.55	0.55	-0.94	0.58	0.57
PPUTLS46_025018	outer membrane porin	22.71	22.76	22.72	22.84	22.49	22.16	0.46	0	0.03	1.1
PPUTLS46_013678	transcriptional regulator MvaT, P16 subunit	22.69	21.81	22.68	22.94	21.72	20.6	0.2	-0.05	1.29	1.11
PPUTLS46_005826	phosphoglycerate kinase	22.66	22.3	21.83	21.61	21.85	21.21	-0.66	-0.34	8.97	2.08
PPUTLS46_003872	multifunctional nucleoside diphosphate	22.6	23.09	23.05	22.14	22.7	22.89	-0.03	0.31	1.09	0.53
PPUTLS46_020876	TolC family type I secretion outer membrane	22.58	22.47	23.21	22.98	22.91	23.37	0.39	0.42	2.97	1.9
PPUTLS46_007201	polynucleotide phosphorylase/polyadenylase	22.56	21.51	22.4	22.05	21.62	21.19	0.1	-0.08	0.83	0.78
PPUTLS46_023963	50S ribosomal protein L24	22.55	22.38	22.26	21.39	21.81	22.38	-0.27	-0.22	1.68	0.49
PPUTLS46_007121	carbamoyl phosphate synthase large subunit	22.54	21.86	21.9	21.57	22.15	21.82	-0.29	0.22	2.74	0.21
PPUTLS46_004129	phosphoenolpyruvate synthase	22.53	21.34	21.02	20.51	21.84	21.62	-0.67	0.24	2.36	0.61
PPUTLS46_000255	3-ketoacyl-(acyl-carrier-protein) reductase	22.5	21.57	21.97	21.23	20.73	21.2	0.03	-0.35	0.99	1.69
PPUTLS46_016404	tryptophan synthase subunit alpha	22.49	21.95	20.53	20.4	20.36	21.08	-1.53	-0.62	9.41	2.11
PPUTLS46_023948	30S ribosomal protein S8	22.48	21.59	22.22	21.37	22.19	21.6	-0.22	0.37	0.51	0.25
PPUTLS46_023468	acetyl-CoA carboxylase biotin carboxylase	22.47	22.2	22.7	22.3	21.59	22.02	0.11	-0.03	2	0.83
PPUTLS46_006196	adenylosuccinate synthetase	22.47	21.68	21.86	21.89	20.4	20.69	-0.02	-0.81	1.13	3.1
PPUTLS46_012735	heat shock protein 90	22.46	21.53	20.66	20.45	20.93	20.69	-1.08	-0.49	4.22	2.38
PPUTLS46_021721	ribonucleotide-diphosphate reductase subunit	22.44	22.16	16.59	17.57	21.44	21.64	-4.08	-0.21	4.96	2.02
PPUTLS46_020706	acetyl-CoA synthetase	22.44	22.31	17.51	16.57	19.77	19.18	-4.7	-2.12	10.41	7.32
PPUTLS46_002452	branched-chain amino acid aminotransferase	22.42	22.02	21.97	22.07	20.31	20.95	-0.01	-0.73	0.89	2.48
PPUTLS46_014179	30S ribosomal protein S16	22.41	21.68	21.63	20.63	22.25	21.04	-0.32	-0.35	1.62	0.5
PPUTLS46_014389	adenylate kinase	22.4	21.61	21.93	21.72	22.83	21.7	-0.03	0.19	1.22	0.77
PPUTLS46_022591	carbamate kinase	22.38	21.52	22.01	21.68	22.16	21.84	0	0.06	0.89	0.92
PPUTLS46_023998	30S ribosomal protein S19	22.38	22.44	18.62	16.46	21.78	21.58	-3.36	-0.2	3.32	1.8
PPUTLS46_022076	CapB	22.38	21.52	18.45	17.48	19.53	17.17	-3.29	-1.69	6.71	2.14
PPUTLS46_020636	aspartate kinase	22.35	21.74	21.09	20.71	21.73	21.12	-0.97	0.01	7.99	1.04
PPUTLS46_005261	glutamate synthase subunit alpha	22.34	21.06	20.94	20.38	21.59	21.54	-0.49	0.17	1.9	0.78
PPUTLS46_022466	Pgl	22.34	21.05	22.48	22.37	15.18	15.19	0.31	-5.19	1.49	9.59
PPUTLS46_023883	catalase	22.33	22.44	21.23	20.21	22.27	22.01	-1.08	0.06	2.8	0.92
PPUTLS46_005761	S-adenosyl-L-homocysteine hydrolase	22.33	21.69	22.59	22.35	21.15	21.42	0.3	-0.17	2.58	1.6
PPUTLS46_001542	serine hydroxymethyltransferase	22.32	21.45	21.33	20.85	22.25	21.7	-0.47	0.14	2.57	0.75
PPUTLS46_022661	cold-shock domain-containing protein	22.3	22.8	22.51	20.81	22.7	23.18	-0.18	0.11	1.17	0.88
PPUTLS46_019646	type II citrate synthase	22.3	21.46	21.98	21.26	21.38	22.42	-0.09	0.08	0.69	0.94
PPUTLS46_005816	transketolase	22.29	21.75	22.36	21.97	21.64	21.26	0.04	-0.07	1.07	1.51
PPUTLS46_016884	pyruvate carboxylase subunit A	22.28	22.21	21.01	20.1	22.12	21.44	-1.19	-0.02	3.46	1.13
PPUTLS46_013433	ubiquinol-cytochrome c reductase, iron-sulfur	22.28	21.67	22.2	21.81	21.7	21.4	-0.04	0	0.51	0.82
PPUTLS46_015949	translation elongation factor P (EF-P)	22.27	22.49	21.96	21.39	22.28	22.43	-0.38	0.2	1.98	0.6
PPUTLS46_005841	fructose-1,6-bisphosphate aldolase	22.27	21.54	22.53	21.93	21.67	21.22	0.04	-0.1	0.96	0.6
PPUTLS46_001067	Protein of unknown function (DUF1329)	22.26	21.82	23.22	22.78	22.05	22.03	0.84	0.19	9.66	2.65
PPUTLS46_017104	endoribonuclease	22.25	22.53	22.91	22.43	22.81	22.59	0.09	0.23	1.16	1.46
PPUTLS46_014549	ribosome recycling factor	22.19	22.51	21.27	21.35	22.24	21.96	-0.48	-0.03	1.96	0.88
PPUTLS46_007176	transcription elongation factor NusA	22.19	21.85	22.59	22.17	21	21.23	0.28	-0.35	4.26	2.49
PPUTLS46_025098	phosphoenolpyruvate carboxykinase	22.18	22.45	22.06	21.7	23.07	23.36	-0.15	0.59	1.44	2.12
PPUTLS46_019276	cytochrome c-type protein	22.18	22.12	20.83	20.62			-1.17		6.53	21.26
PPUTLS46_020701	cationic amino acid ABC transporter, periplasmic	22.17	21.82	22.91	22.66	20.04	20.38	0.7	-1.08	10.75	4.43
PPUTLS46_024023	30S ribosomal protein S10	22.15	23.23	21.72	21.99	22.61	23.04	-0.03	0.5	0.94	0.54
PPUTLS46_023988	30S ribosomal protein S3	22.15	20.72	22.27	21.94	21.9	21.08	0.18	0.52	1.24	0.4
PPUTLS46_001857	30S ribosomal protein S20	22.15	16.56	20.82	20.58	19.84	15.48	0.35	-3	0.9	0.32
PPUTLS46_019286	pentapeptide repeat-containing protein	22.15	21.89	19.66	18.94			-2.33		8.45	56.35
PPUTLS46_015954	OsmC family protein	22.14	21.91	21.91	21.1	22.98	23.21	-0.2	1	1.59	4.64
PPUTLS46_019626	succinate dehydrogenase iron-sulfur subunit	22.13	21.26	21.61	21.39	22.8	22.36	-0.02	0.71	1.16	2.76
PPUTLS46_014639	enolase	22.11	22.17	21.95	21.3	20.33	20.34	-0.24	-1.12	1.73	4.84
PPUTLS46_000385	isopropylmalate isomerase large subunit	22.1	21.31	20.18	19.61	21.24	21.38	-1.45	0.03	5.71	1
PPUTLS46_013948	outer membrane porin	22.09	21.14	20.28	20.65	19.2	18.22	-0.62	-1.76	2.01	3.66
PPUTLS46_003452	PpiC-type peptidyl-prolyl cis-trans isomerase	22.08	21.42	20.52	19.93	22.07	22.31	-1.21	0.43	5.69	2.26
PPUTLS46_006296	extracellular ligand-binding receptor	22.07	21.63	23.38	23.8	20.51	20.93	1.14	-0.46	2.87	2.29
PPUTLS46_013943	tricarboxylate transport protein TctC	22.06	20.18	19.77	19.18	18.79	17.99	-0.78	-1.21	1.87	2.15
PPUTLS46_016314	carbonate dehydratase	22.05	21.2	21.1	19.87	21.21	21.55	-0.36	0.05	1.52	0.96
PPUTLS46_000210	ribonuclease E	22.05	22.35	21.81	21.38	20.23	21.21	-0.27	-0.26	1.72	1.33
PPUTLS46_002042	redoxin domain-containing protein	22.04	18.94	21.95	20.88	22.04	20	0.51	1.4	0.73	0.38
PPUTLS46_023953	30S ribosomal protein S14	22.03	21.01	21.03	20.72	21.6	19.91	-0.31	-0.43	1.89	0.59
PPUTLS46_010254	alkyl hydroperoxide reductase, C subunit	22.01	21.27	20.62	20.27	22.4	22.03	-0.96	0.51	5.55	2.68
PPUTLS46_022426	inosine 5'-monophosphate dehydrogenase	22.01	21.8	22.16	22.12	21.63	21.78	0.08	0.18	0.8	0.31
PPUTLS46_024348	30S ribosomal protein S21	21.98	22.01	21.61	20.88	21.89	22.18	-0.42	0.15	2.12	0.7
PPUTLS46_022211	glycerol-3-phosphate dehydrogenase	21.98	20.86	21.18	20.4	17.22	18.37	-0.05	-2.24	1.15	3.28
PPUTLS46_005601	protein PhaF	21.97	21.11	24.62	24.2	24.91	24.04	2.49	2.42	9.27	5.1
PPUTLS46_005736	Uncharacterized conserved protein	21.96	22.01	21.96	21.31	20.86	21.6	-0.12	-0.12	1.42	0.78

PPUTLS46_023121	fumarate hydratase	21.95	22.08	21.84	21.06	21.84	22.01	-0.24	0.24	1.58	0.46
PPUTLS46_025298	succinate-semialdehyde dehydrogenase I	21.93	20.77	20.63	20.53	21.13	21.84	-0.39	0.1	1.81	1.09
PPUTLS46_004664	UspA domain-containing protein	21.93	21.22	21.52	21.04	20.65	22.24	-0.06	0.24	1.39	0.82
PPUTLS46_022456	2-isopropylmalate synthase	21.92	21.1	20.35	19.63	21.03	21.15	-1.07	0.03	3.69	1
PPUTLS46_021711	ribonucleotide-diphosphate reductase subunit	21.9	21.98	19.61	19.5	22.03	21.6	-1.99	0.05	7.06	1.06
PPUTLS46_007346	acetolactate synthase 3 catalytic subunit	21.86	20.82	20.07	19.36	20.97	21.72	-1.08	0.04	3.23	1.02
PPUTLS46_023463	acetyl-CoA carboxylase biotin carboxyl carrier	21.85	21.68	20.87	20.57	20.04	20	-0.9	-1.22	8.24	9.61
PPUTLS46_017434	50S ribosomal protein L33	21.81	21.56	20.94	20.39	21.6	20.97	-0.8	0.03	5.32	1
PPUTLS46_011130	UTP-glucose-1-phosphate uridylyltransferase	21.81	19.9	20.73	20.62	21.6	19.64	-0.06	1.46	0.91	0.09
PPUTLS46_022706	histone family protein DNA-binding protein	21.8	20.55	22.59	22.12	21.83	19.79	0.65	-0.85	2.14	0.37
PPUTLS46_016399	tryptophan synthase subunit beta	21.8	19.54	21.16	20.16	19.42	18.13	-0.66	-0.08	0.46	1.07
PPUTLS46_020641	alanyl-tRNA ligase	21.78	21.75	21.13	21.64	21.07	21.06	-0.2	-0.16	0.67	1.8
PPUTLS46_016304	hypothetical protein	21.78	20.44	21.14	20.66	19.8	19.4	-0.11	-0.52	0.75	1.84
PPUTLS46_022461	keto-hydroxyglutarate-aldolase/keto-deoxy-	21.78	18.96	21.41	20.3			0.74		0.56	1.79
PPUTLS46_022396	phosphoribosylformylglycinamidine synthase	21.77	20.35	20.83	20.23	21.31	20.73	0.02	0.44	0.99	0.45
PPUTLS46_014719	recombinase A	21.77	22.27	20.51	20.45	19.89	20.41	-0.95	-0.76	2.71	2.11
PPUTLS46_013553	cell division protein FtsZ	21.76	21.18	17.74	18.27	21.61	21.08	-2.72	0.17	4.96	0.54
PPUTLS46_001257	ribose-phosphate pyrophosphokinase	21.76	21.32	21.69	21.36	20.37	20.7	0.02	-0.39	1.18	2.3
PPUTLS46_019686	electron transfer flavoprotein subunit alpha	21.73	20.81	21.38	21.02	22.67	22.93	-0.04	1.31	0.78	3.85
PPUTLS46_023983	50S ribosomal protein L16	21.72	19.84	21.31	20.56	20.53	17.47	0.48	-0.56	0.54	0.73
PPUTLS46_016544	glucose-methanol-choline oxidoreductase	21.71	20.55	19.68	18.98			-1.21		3.22	6.48
PPUTLS46_004866	Domain of unknown function (DUF4399)	21.7	20.83	21.53	21.2	21.15	20.85	0.09	-0.18	0.81	0.41
PPUTLS46_023493	acetoin dehydrogenase E2 subunit	21.7	21.38	19.41	18.63			-2.09		6.97	110.64
PPUTLS46_013443	ubiquinol--cytochrome c reductase, cytochrome c1	21.62	21.32	21.78	21.56	20.49	21.11	0.09	0.01	1.67	0.96
PPUTLS46_022696	valyl-tRNA ligase	21.62	20.49	21.58	21.03	19.46	20.17	0.2	-0.38	0.67	1.53
PPUTLS46_018356	aldehyde dehydrogenase	21.61	20.71	20.96	20.8	18.79	17.13	-0.07	-1.69	1.31	2.62
PPUTLS46_000405	aspartate-semialdehyde dehydrogenase	21.6	20.76	20.11	19.7	21.05	20.77	-0.97	-0.15	4.44	0.43
PPUTLS46_000240	50S ribosomal protein L32	21.6	20.05	19.49	17.61	20.51	20.78	-0.8	0.13	1.58	0.88
PPUTLS46_008999	glyoxalase/bleomycin resistance	21.6	21.14	21.12	21.32			-0.06		0.78	58.93
PPUTLS46_012405	elongation factor G	21.59	21.64	22.26	21.67	22.13	21.73	0.17	0.33	1.41	1.9
PPUTLS46_022131	argininosuccinate synthase	21.59	21.03	21.15	20.55	21.22	21.49	-0.21	0.12	1.98	1.28
PPUTLS46_007096	heat shock protein GrpE	21.57	21.18	20.88	20.59	21.37	20.78	-0.58	-0.05	19.38	0.73
PPUTLS46_012465	NADH dehydrogenase subunit G	21.56	20.24	21.93	21.41	20.68	20.82	0.24	0.12	1.34	0.87
PPUTLS46_014494	2,3,4,5-tetrahydropyridine-2,6-carboxylate	21.55	20.89	20.88	20.62	20.92	20.78	-0.3	0	2.87	0.84
PPUTLS46_022166	bacterioferritin	21.53	20.16	23.1	22.67	22.47	21.63	1.41	0.6	3.26	1.71
PPUTLS46_021486	peptidoglycan-associated lipoprotein	21.52	23.03	21.9	22.26	22.51	22.98	-0.6	0.43	0.5	0.7
PPUTLS46_002837	cyclophilin type peptidyl-prolyl cis-trans	21.52	21.51	21.3	20.37	20.76	20.24	-0.28	-0.43	1.6	2.3
PPUTLS46_007934	glycerate kinase	21.5	20.79	20.39	20.32			-0.52		2.93	15.11
PPUTLS46_007434	enoyl-CoA hydratase/isomerase	21.48	20.35	21.18	21.03	20.36	19.93	0.04	0.01	0.97	0.96
PPUTLS46_014629	CTP synthetase	21.47	20.78	21.98	21.77	20.94	21	0.54	0.03	3.42	0.98
PPUTLS46_021361	Protein of unknown function (DUF4223)	21.47	17.34	19.51	18.2	20.64	21.11	-0.92	0.14	0.61	1.04
PPUTLS46_005421	GTP-binding protein TypA	21.46	20.89	20.39	20.32	21.12	21.05	-0.59	0.08	3.65	1.4
PPUTLS46_017504	cytochrome c5	21.45	21.84	21.39	21.04	22.17	22.42	-0.09	0.33	1.28	1.53
PPUTLS46_006181	HflK protein	21.45	21.53	21.82	21.58	21.26	20.83	0.06	-0.02	0.89	1.15
PPUTLS46_004871	D-3-phosphoglycerate dehydrogenase	21.44	21.25	21.6	21.27	21.83	20.68	0.03	0.13	1.01	0.86
PPUTLS46_007126	transcription elongation factor GreA	21.44	21.24	19.49	17.52	20.3	20.63	-1.57	-0.22	2.23	1.74
PPUTLS46_021536	aspartyl-tRNA ligase	21.43	20.1	21.52	20.99	20.75	20.24	0.06	-0.4	0.95	0.37
PPUTLS46_015189	Fe3+ ABC transporter periplasmic component-like	21.42	21.18	22.67	21.98	21.89	21.51	0.74	0.49	3.42	3.47
PPUTLS46_016674	F0F1 ATP synthase subunit gamma	21.41	21.63	21.61	20.95	21.95	21.6	-0.05	0.23	1.18	1.48
PPUTLS46_001132	lipoprotein	21.39	21	21.5	20.86	21.42	21.41	-0.04	0.4	0.69	5.1
PPUTLS46_019866	cbb3-type cytochrome c oxidase subunit II	21.38	21.64	21.88	21.37	21.64	22.11	0.04	0.04	1.02	1.01
PPUTLS46_009904	sulfite reductase	21.38	21.25	19.28	19.77	20.92	21.08	-1.09	-0.17	2.68	0.23
PPUTLS46_007281	C4-type zinc finger DksA/TraR family protein	21.38	20.95	21.17	20.11	20.54	20.76	-0.02	-0.03	1.09	1.23
PPUTLS46_000982	thioredoxin reductase	21.37	20.58	16.25	18.7	20.78	21.11	-1.54	0.06	1.71	1.08
PPUTLS46_009549	ATP-dependent protease ATP-binding subunit ClpX	21.36	21.64	20.08	20.29	21.46	21.29	-0.7	0.11	2.24	0.82
PPUTLS46_015009	membrane protein involved in aromatic	21.33	20.91	21.2	20.57	24.58	24.36	-0.02	3.32	1.23	52.8
PPUTLS46_024043	30S ribosomal protein S12	21.33	18.34	20.56	18.45	21.37	20.87	-1.55	0.09	0.25	1.03
PPUTLS46_012715	3-oxoacyl-(acyl carrier protein) synthase I	21.33	20.27	21.18	20.31	20.27	20.04	-0.47	0.02	0.16	0.97
PPUTLS46_022421	GMP synthase	21.32	20.96	21.84	21.61	19.83	20.78	0.49	0.02	6.74	0.98
PPUTLS46_025293	4-aminobutyrate aminotransferase	21.28	21.01	21.08	20.58	21.23	20.89	-0.19	0.1	2.52	1.45
PPUTLS46_008599	CopA family copper resistance protein	21.28	21.19	20.84	20.92	18.72	17.96	0.02	-2.03	1.03	5.58
PPUTLS46_013378	bifunctional sulfate adenylyltransferase subunit	21.27	21.59	20.44	19.91	20.92	20.7	-0.83	-0.03	2.9	1.14
PPUTLS46_014259	NADH:flavin oxidoreductase/NADH oxidase	21.25	21.95	21.63	21.86	20.93	21.33	0.53	-0.42	0.37	0.43
PPUTLS46_005196	arginyl-tRNA ligase	21.25	20.95	21.02	20.76	19.77	19.22	-0.14	-0.98	3.39	4.59
PPUTLS46_023878	bacterioferritin	21.24	20.89	22.39	22.21	20.99	21.13	1.08	0.1	9.78	1.34
PPUTLS46_014579	outer membrane chaperone Skp	21.24	20.27	20.95	21.04	19.53	20.92	0.06	-0.06	1.05	0.92
PPUTLS46_024153	histidine triad	21.23	21.57	19.3	19.51	20.55	20.5	-1.32	-0.16	3.21	1.41
PPUTLS46_011960	isocitrate dehydrogenase	21.22	20.2	23.03	22.58	21.18	21.08	1.65	0.31	5.07	1.65
PPUTLS46_019876	cytochrome c oxidase, cbb3-type subunit III	21.22	21.36	21.45	21.3	20.88	22.58	0.19	0.41	0.54	0.7
PPUTLS46_004524	Domain of unknown function (DUF4404)	21.2	21.71	20.31	18.63	20.53	20.83	-1	-0.08	1.86	0.83
PPUTLS46_010564	threonyl-tRNA ligase	21.19	19.7	21.45	20.56	20.14	18.83	0.26	-0.39	0.71	0.61
PPUTLS46_023498	alcohol dehydrogenase	21.19	21.33	19.79	19.76			-1.05		3.75	10.32
PPUTLS46_001922	hypothetical protein	21.18	21.38	23	23.12	22.23	22.46	1.23	0.82	3.4	2.77
PPUTLS46_014764	oxidoreductase FAD/NAD(P)-binding subunit	21.18	20.3	20.89	19.96	18.04	18.51	-0.19	-1.55	0.61	4.01
PPUTLS46_010034	Domain of unknown function (DUF4174)	21.17	20.05	21.87	21.43	20.46	20.25	0.59	-0.17	2.22	0.6
PPUTLS46_003417	ecotin	21.16	20.88	21.64	21.23	21.81	22.1	0.33	0.85	4.43	3.78
PPUTLS46_001527	ABC transporter ATP-binding protein	21.14	21.2	21.09	20.92	20.7	20.56	-0.02	-0.04	0.81	1.23
PPUTLS46_003497	type VI secretion system effector, Hcp1 family	21.13	21.37	21.8	21.22	21.64	22.63	0.1	0.27	1.16	1.28
PPUTLS46_012998	trans-2-enoyl-CoA reductase	21.13	21.4	21	20.6	21.43	20.68	-0.17	0.05	1.51	1.03
PPUTLS46_011985	adenylosuccinate lyase	21.12	19.88	19.77	19.8	20.21	18.27	-0.3	-0.25	1.57	0.79
PPUTLS46_016524	glycyl-tRNA ligase subunit beta	21.11	20.6	20.89	20.62	20.44	20.14	-0.03	-0.12	1.64	2.02
PPUTLS46_017909	transcription termination factor Rho	21.1	20.2	21.55	20.74	20.67	20.33	0.07	0.23	0.92	0.46
PPUTLS46_002972	saccharopine dehydrogenase	21.1	20.51	19.75	19.2	20.34	20.19	-1.07	-0.1	6.09	1.91
PPUTLS46_007326	Uncharacterized protein conserved in bacteria	21.09	20.89	20.59	19	21.48	21.38	-0.34	0.56	1.37	5.51
PPUTLS46_000015	DEAD-box ATP dependent DNA helicase	21.08	20.37	20.26	19.65	19.42	18.55	-0.45	-0.84	2.59	2.63

PPUTLS46_006186	HflC protein	21.06	21.32	21.26	21.43	21.32	20.8	0.44	0.05	0.27	1.04
PPUTLS46_019851	Uncharacterized protein conserved in bacteria	21.06	20	16.19	15.68	19.54	20.17	-4.04	-0.05	10.3	1.11
PPUTLS46_010569	bacterial translation initiation factor 3	21.04	20.84	20.99	20.52	20.74	20.55	-0.07	0.03	1.66	1.04
PPUTLS46_022681	multifunctional aminopeptidase A	21.03	20	22.81	21.92	21.32	19.37	1.21	0.75	3	0.44
PPUTLS46_023978	50S ribosomal protein L29	21.03	17.75	20.52	19.21	19.7	16.37	0.95	-1.78	0.52	0.36
PPUTLS46_021401	phosphoribosylaminoimidazole-succinocarboxamide	21.03	17.28	20.76	20.21	18.7	18.47	0.14	-0.64	0.95	0.7
PPUTLS46_007146	Cell division protease ftsH	21.01	20.4	20.58	20.36	19.81	19.69	-0.1	-0.48	1.85	4.23
PPUTLS46_017819	hypothetical protein	21	21.48	19.53	18.39	21.14	21.24	-1.52	0.26	2.87	0.61
PPUTLS46_004149	ribonuclease activity regulator protein RraA	20.98	19.88	20.67	19.85	21.63	20.21	-0.33	0.28	0.38	0.75
PPUTLS46_004114	NAD-glutamate dehydrogenase	20.98	20.87	22.05	21.84	19.06	19.15	0.81	-1.2	5.16	6.37
PPUTLS46_024008	50S ribosomal protein L23	20.97	19.86	20.12	20.26	20.09	19.33	-0.01	-0.19	1.08	0.66
PPUTLS46_021936	17 kDa surface antigen	20.96	21.03	21.61	21.25	21.47	21.32	0.23	0.39	1.7	2.23
PPUTLS46_001072	Protein of unknown function (DUF1302)	20.96	20.53	22.19	21.62	20.17	20.16	0.95	-0.18	5.75	3.19
PPUTLS46_021986	OmpA/MotB domain protein	20.94	20.61	22.98	23.36	20.51	22.45	1.78	0.11	4.1	0.94
PPUTLS46_026006	catalase/hydroperoxidase HPI(I)	20.93	20.5	23.49	23.22	20.93	20.92	2.51	0.38	44.88	4.56
PPUTLS46_025228	cystine transporter subunit	20.93	19.27	22.4	22.01	20.11	19.92	1.34	0.29	2.68	0.69
PPUTLS46_021151	cysteine synthase A	20.93	20.69	21.06	21	18.63	20.39	0.07	0.01	0.82	0.99
PPUTLS46_013428	30S ribosomal protein S9	20.92	20.95	21.32	21.18	21.14	19.89	0.04	-0.02	1.03	0.94
PPUTLS46_026201	glycolate oxidase subunit GlcD	20.92	19.44	21.91	21.33			0.76		2.04	3.46
PPUTLS46_001497	ATP-dependent protease	20.91	20.62	21.24	20.89	20.55	20.57	0.25	0.1	5.09	0.44
PPUTLS46_014614	acetyl-CoA carboxylase carboxyltransferase	20.91	20.59	20.94	20.5	19.94	20.08	0.02	-0.24	1.16	2.38
PPUTLS46_007111	dihydrodipicolinate reductase	20.89	20.83	20.07	20.24	20.03	20.06	-0.25	-0.27	1.63	2.3
PPUTLS46_013393	Uncharacterized protein conserved in bacteria	20.87	20.4	20.6	20.03	19.68	19.53	-0.12	-0.62	1.77	10.66
PPUTLS46_026206	glycolate oxidase FAD binding subunit	20.87	20.19	21.35	20.58			0.04		1.04	13.82
PPUTLS46_003552	type VI secretion protein, EvpB/VC_A0108 family	20.86	20.78	20.51	19.72	21.2	21.83	-0.38	0.4	1.99	1.68
PPUTLS46_024133	N-acetyl-gamma-glutamyl-phosphate reductase	20.86	19.99	20.46	20.77	20.75	20.74	0.06	0.29	0.94	1.76
PPUTLS46_015384	fumarate hydratase	20.86	19.3	21.68	20.06	19.74	18.78	0.53	-0.38	0.6	0.57
PPUTLS46_025403	argininosuccinate lyase	20.86	19.35	20.57	20.35	19.72	18.39	0.06	-0.33	0.96	0.68
PPUTLS46_005806	S-adenosylmethionine synthetase	20.85	19.68	20.71	19.82	20.62	20.49	-0.51	0.13	0.16	1.19
PPUTLS46_006551	bifunctional	20.85	21.35	20.58	20.37	20.41	20.75	-0.17	-0.31	1.37	0.49
PPUTLS46_025488	type I secretion C-terminal target domain	20.84	21.25	21.42	20.92	22.68	22.35	0.04	1.17	0.98	3.16
PPUTLS46_019926	coproporphyrinogen III oxidase	20.84	21.02			19.67	19.04		-0.82	11.78	2.75
PPUTLS46_001807	hypothetical protein	20.83	20.14	18.84	18.52	20.24	19.13	-1.57	-0.04	9.4	0.89
PPUTLS46_008644	copper-translocating P-type ATPase	20.82	19.88	20.64	20.11	20.06	20.13	-0.19	0.08	0.41	0.87
PPUTLS46_022911	rod shape-determining protein MreB	20.81	20.61	20.21	19.79	20.37	20.13	-0.56	-0.08	5.48	1.8
PPUTLS46_015444	DNA gyrase subunit A	20.81	20.87	20.91	20.95	19.92	20.28	0.35	0.01	0.18	1.04
PPUTLS46_015644	2-dehydro-3-deoxyphosphooctonate aldolase	20.8	19.53	19.69	19.49	19.18	21.38	-0.19	0.17	1.39	0.92
PPUTLS46_015709	alcohol dehydrogenase	20.77	20.6	20.71	20.22	20.39	20.31	-0.09	0	1.72	0.82
PPUTLS46_013568	preprotein translocase subunit SecA	20.77	19.71	19.58	19.77	18.77	19.41	-0.21	-0.34	1.41	1.54
PPUTLS46_015129	FKBP-type peptidylprolyl isomerase	20.76	20.97	21.16	20.94	20.53	21.03	0.11	0.44	0.78	0.28
PPUTLS46_014154	phosphoribosylglycinamide formyltransferase 2	20.76	19.14	19.74	19.91	19.64	20.08	0.03	0.08	0.99	0.94
PPUTLS46_021416	dihydrodipicolinate synthase	20.75	18.95	19.75	18.86	20.58	18.99	-0.24	1.2	0.72	0.13
PPUTLS46_016249	NLPA lipoprotein	20.74	21.15	21.12	21.34	20.63	20.66	0.4	-0.19	0.44	0.58
PPUTLS46_005266	glutamate synthase subunit beta	20.74	20.3	19.7	19.95	20.51	20.69	-0.28	0.16	1.67	1.6
PPUTLS46_006171	RNA-binding protein Hfq	20.74	20.65	20.64	20.07	19.81	18.5	-0.15	-0.56	1.7	1.74
PPUTLS46_024183	indole-3-glycerol-phosphate synthase	20.74	19.88	20.63	19.13	19.51	19.6	-0.38	-0.17	0.59	1.55
PPUTLS46_000325	aromatic amino acid aminotransferase	20.73	20.4	19.89	20.25	20.39	19.38	-0.01	0.01	1.08	1.03
PPUTLS46_014339	lysyl-tRNA ligase	20.73	20.18	20.32	19.96	19.87	19.7	-0.21	-0.24	3.18	3.34
PPUTLS46_015224	septum site-determining protein MinD	20.72	20.54	21.05	20.43	21.76	20.6	0.04	0.28	1.03	1.36
PPUTLS46_014224	threonine synthase	20.72	19.29	20.67	20	18.67	19.88	0.28	-0.01	0.63	1.04
PPUTLS46_024078	transcription antitermination protein NusG	20.71	21.03	18.93	20.25	20.43	20.68	0.02	-0.33	0.99	0.31
PPUTLS46_012450	bifunctional NADH:ubiquinone oxidoreductase	20.7	20.75	21.04	20.54	20.86	20.69	0.04	0.11	1.04	1.3
PPUTLS46_022821	RpoX	20.7	20.61	20.15	19.27	20.59	20.27	-0.53	0.04	2.2	1.06
PPUTLS46_021591	prolyl-tRNA ligase	20.69	21.04	20.63	20.82	20.59	21.54	-0.38	0.44	0.33	0.53
PPUTLS46_016179	cytochrome c4	20.69	19.92	21.42	21.27	19.39	20.22	0.77	-0.01	3.66	0.94
PPUTLS46_005361	phosphoglyceromutase	20.67	20.28	20	19.71	19.87	19.07	-0.57	-0.31	18.94	1.76
PPUTLS46_004209	ribosome modulation factor	20.66	19.28	20.49	19.25	20.66	20.01	-0.78	0.15	0.14	0.84
PPUTLS46_014659	S-(hydroxymethyl)glutathione dehydrogenase	20.66	19.66	19.69	19.1	18.95	19.67	-0.33	-0.13	1.86	1.23
PPUTLS46_000400	3-isopropylmalate dehydrogenase	20.65	20.76	18.72	19.09	20.71	20.42	-1.13	0.03	2.86	1
PPUTLS46_022926	aspartyl/glutamyl-tRNA amidotransferase subunit	20.64	20.77	20.33	20.15	20.46	20.67	-0.16	0.3	1.55	0.34
PPUTLS46_006691	leucyl-tRNA ligase	20.64	20.35	19.41	19.08	19.92	19.91	-1.17	-0.17	23.73	2.93
PPUTLS46_024358	RNA polymerase sigma factor RpoD	20.64	21.38	20.92	20.88	19.9	19.95	-0.26	-0.13	0.6	1.22
PPUTLS46_004399	multifunctional fatty acid oxidation complex	20.63	20.5	20.1	19.23	25.57	25.39	-0.5	4.68	2.18	30.61
PPUTLS46_023693	6,7-dimethyl-8-ribityllumazine synthase	20.63	20.22	21.99	21.7	20.64	19.99	1.34	0.1	35.23	0.79
PPUTLS46_020676	bifunctional	20.63	20	20.25	19.86	19.37	19.38	-0.11	-0.44	1.89	3.35
PPUTLS46_007086	ferric uptake regulation protein	20.63	18.15	21.62	21.03	19.22	17.04	0.78	-0.98	1.58	0.48
PPUTLS46_022746	bifunctional histidinal dehydrogenase/	20.61	19.47	19.67	19.97	19.53	19.12	0	-0.04	1.05	0.88
PPUTLS46_005631	ATP-dependent protease ATP-binding subunit HslU	20.59	20.14	19.87	19.27	20.15	19.98	-0.55	0.01	3.64	0.73
PPUTLS46_013343	AapJ	20.59	20.25	24.23	23.9	17.54	16.87	3.52	-2.4	133.87	7.91
PPUTLS46_006646	serine-type D-Ala-D-Ala carboxypeptidase	20.58	20.38	20.93	20.31	19.82	19.03	0.04	-0.38	1.05	1.92
PPUTLS46_006546	phosphoribosylamine--glycine ligase	20.58	19.9	19.85	20.16	19.05	19.88	0.01	-0.04	0.96	1.1
PPUTLS46_022196	MIP family channel protein	20.58	20.4	17.84	17.78	18.99	18.96	-2.35	-1.01	10.55	8.3
PPUTLS46_014694	RNA polymerase sigma factor RpoS	20.58	19.16	14.33	16.59	17	16.99	-2.52	-1.76	2.19	3.59
PPUTLS46_016689	UDP-N-acetylglucosamine pyrophosphorylase	20.56	20.19	20.56	20.32	19.55	19.86	0.06	-0.1	0.59	1.42
PPUTLS46_024508	malate synthase G	20.55	20.17	19.52	18.81	23.3	23.07	-0.86	2.84	3.98	47.08
PPUTLS46_011185	carbon storage regulator	20.53	20.28	21.5	21.04	21.26	21.34	0.72	0.93	6.27	6.35
PPUTLS46_015459	bifunctional cyclohexadienyl dehydrogenase/	20.53	20.25	20.13	20.02	20.14	18.59	-0.12	-0.05	1.83	1.08
PPUTLS46_024448	acyl-CoA dehydrogenase	20.52	15.78	21.11	19.01	24.21	24.44	0.83	3.57	0.73	2.81
PPUTLS46_016684	F0F1 ATP synthase subunit epsilon	20.52	20.99	21.05	21.87	21.88	20.02	0.42	0.03	0.64	1
PPUTLS46_007116	carbamoyl phosphate synthase small subunit	20.52	20.16	20.43	19.97	18.96	18.28	-0.05	-1.01	1.74	3.91
PPUTLS46_005121	thiazole synthase	20.52	19.36	18.83	18.6	18.81	18.54	-0.78	-0.43	2.7	1.86
PPUTLS46_014269	gamma-aminobutyraldehyde dehydrogenase	20.51	19.73	20.72	20.7	20.13	20.45	0.32	0.18	1.85	1.35
PPUTLS46_006191	ATP phosphoribosyltransferase regulatory subunit	20.51	19.64	19.75	19.6	18.71	19.14	-0.17	-0.43	1.6	1.91
PPUTLS46_003902	scaffold protein	20.51	19.5	17.45	17.51	18.67	18.33	-2.02	-0.7	5.18	2.66
PPUTLS46_001842	isoleucyl-tRNA ligase	20.5	20.32	20.24	20	19.68	19.65	-0.14	-0.29	2.19	3.23

PPUTLS46_009594	methyl-accepting chemotaxis sensory transducer	20.49	20.46	20.22	20.78	20.67	21.12	-0.46	0.22	0.33	1.42
PPUTLS46_016174	thiol:disulfide interchange protein DsbA	20.49	19.33	21.2	20.97	19.35	17.99	0.74	0.01	2.47	0.98
PPUTLS46_006736	glutamate-1-semialdehyde aminotransferase	20.47	19.84	19.41	18.68	20.04	19.76	-0.73	-0.12	3.17	0.22
PPUTLS46_016334	oligopeptidase A	20.47	19.45	19.9	20.15	19.59	17.21	0.05	-0.08	0.96	0.93
PPUTLS46_006686	rare lipoprotein B	20.46	20.73	21.09	21.45	20.29	19.87	0.07	-0.03	0.93	1.12
PPUTLS46_025188	peroxidase	20.46	20.41	20.69	20.94	20.09	20.69	0.18	0.3	0.7	0.5
PPUTLS46_015664	lipopolysaccharide biosynthesis protein	20.46	19.12	20.55	20.03	19.38	19.38	0.05	-0.1	0.96	0.8
PPUTLS46_004007	phosphonate ABC transporter periplasmic	20.45	19.51	20.87	20.6	19.31	19.04	0.45	-0.13	2.21	1.41
PPUTLS46_000455	amidophosphoribosyltransferase	20.44	19.38	19.49	18.88	19.15	18.76	-0.26	-0.16	1.62	1.4
PPUTLS46_022921	aspartyl/glutamyl-tRNA amidotransferase subunit	20.44	18.53	19.64	18.67	17.47	18.97	-0.48	-0.21	0.52	1.16
PPUTLS46_007914	aldo/keto reductase	20.43	19.75	18.32	19.13	18.77	19.52	-0.58	-0.18	1.67	1.33
PPUTLS46_012460	NADH dehydrogenase I subunit F	20.42	19.64	21.41	20.62	19.99	19.74	0.53	0.16	2.14	0.54
PPUTLS46_009719	aconitate hydratase	20.41	20.18	20.29	19.82	19.94	20.34	-0.12	0.21	2.08	0.53
PPUTLS46_023888	50S ribosomal protein L17	20.37	19.06	21.05	19.95	21.07	19.01	0.12	0.73	0.89	0.5
PPUTLS46_013173	2-hydroxyacid dehydrogenase	20.36	19.96	18.42	18.78	17.52	18.19	-1.02	-1.37	2.92	3.59
PPUTLS46_025746	isoquinoline 1-oxidoreductase, beta subunit	20.34	20.86	21.6	20.47	17.19	14.95	0.14	-2.7	1.13	2.81
PPUTLS46_026216	Uncharacterized protein, possibly involved in	20.3	17.92	21.1	18.98			1.02		0.47	1.33
PPUTLS46_004229	Uncharacterized protein conserved in bacteria	20.27	17.46	19.14	19.29	18.35	16.87	0.12	-0.75	0.94	0.57
PPUTLS46_013458	transport-associated protein	20.26	19.78	20.21	19.82	19.54	18.9	-0.01	-0.18	0.5	1.6
PPUTLS46_008589	copper resistance B	20.25	20.19	19.35	18.96	16.57	15.2	-0.84	-3	5.33	4.59
PPUTLS46_022756	UDP-N-acetylglucosamine	20.24	19.69	20.11	19.68	19.68	19.23	0	0.02	0.75	1.06
PPUTLS46_024403	pyrroloquinoline quinone biosynthesis protein	20.24	19.05	19.51	19.78			0.02		0.98	3.7
PPUTLS46_010589	phenylalanyl-tRNA ligase subunit beta	20.23	19.73	20.57	20.25	19.24	19.8	0.34	-0.04	5.32	0.86
PPUTLS46_006606	gamma-glutamyl phosphate reductase	20.22	19.17	18.68	18.27	19.71	19.11	-0.8	-0.43	3.03	0.16
PPUTLS46_011945	ATP-dependent Clp protease ATP-binding subunit	20.2	19.68	18.14	18.71	18.78	17.32	-0.84	-0.71	2.23	1.84
PPUTLS46_019696	electron-transferring-flavoprotein dehydrogenase	20.19	19.65	20.23	19.85	23.76	23.59	0.05	3.64	0.84	32.11
PPUTLS46_000420	peptidoglycan-binding LysM	20.19	20.31	20.72	20.41	20.82	20.12	0.1	0.21	1.24	1.35
PPUTLS46_025378	HemY domain-containing protein	20.19	19.28	20.96	20.32	19.7	19.05	0.48	-0.36	2.11	0.2
PPUTLS46_005926	carbamoyltransferase	20.19	19.65	20.44	19.7	19.18	19	0.19	-0.4	0.53	4.59
PPUTLS46_001097	Zn-dependent protease with chaperone function	20.18	20.73	20.77	20.37	20.48	20.52	0.09	0.19	0.9	0.74
PPUTLS46_017809	nitrogen regulatory protein P-II	20.18	19.33	23.3	22.85	19.99	20.08	2.93	0.27	10.56	1.67
PPUTLS46_006236	30S ribosomal protein S18	20.17	20.06	21.16	20.4	21.03	19.8	0.38	0.1	1.91	1.09
PPUTLS46_017429	50S ribosomal protein L28	20.17	19.34	19.57	20.35	18.18	18.8	0.2	-0.46	0.82	1.83
PPUTLS46_014289	extracellular solute-binding protein	20.15	19.31	22.01	21.18	19.79	20.36	1.33	0.25	3.7	1.39
PPUTLS46_021491	translocation protein TolB	20.15	20.24	20.06	19.96	19.38	19.65	-0.06	0.02	0.72	0.98
PPUTLS46_003907	cysteine desulfurase	20.14	20.32	19.07	18.56	18.22	17.71	-1.05	-1.47	3.92	4.44
PPUTLS46_001272	GTP-binding protein YchF	20.13	16.4	19.28	19.34	19.68	17.94	0.04	1.37	0.99	0.45
PPUTLS46_016589	DNA polymerase III subunit beta	20.11	19.12	20.5	19.35	20.66	19.81	0.48	0.23	0.4	1.31
PPUTLS46_013473	LppC family lipoprotein	20.11	20.04	20.19	20.01	19.54	19.94	-0.09	-0.26	0.43	0.29
PPUTLS46_015154	carboxyl-terminal protease	20.11	19.25	20.66	20.81	19.46	18.93	0.64	-0.18	2.26	0.51
PPUTLS46_023568	inorganic pyrophosphatase	20.1	21.08	20.97	19.71	21.4	21.3	-0.02	0.27	1.04	1.27
PPUTLS46_013548	cell division protein FtsA	20.08	20.06	20.17	20.33	19.96	19.65	0.32	0.03	0.35	1.01
PPUTLS46_009314	surface antigen (D15)	20.08	20.32	19.58	19.75	19.83	20.37	0.02	0.49	1.02	0.23
PPUTLS46_016944	N5-carboxyaminoimidazole ribonucleotide mutase	20.08	19.61	19.66	19.56	18.36	18.61	-0.07	-0.75	1.53	3.87
PPUTLS46_018661	quinone oxidoreductase	20.08	19.8	17.28	16.7	17.66	18.15	-2.67	-1.22	14.1	3.97
PPUTLS46_001077	long-chain-fatty-acid--CoA ligase	20.08	17.6	20.89	20.7	16.62	16.44	0.9	-0.7	1.67	1.53
PPUTLS46_004559	transaldolase B	20.07	19.09	19.33	19.67	20.03	18.84	0	0.59	0.95	0.3
PPUTLS46_014369	OmpA/MotB domain-containing protein	20.07	19.71	19.71	19.3	16.7	17.01	-0.3	-2.26	6.01	8.54
PPUTLS46_013768	Toluene efflux pump outer membrane protein ttgC	20.06	19.79	20.09	19.82	19.73	19.46	0	0	0.55	1.31
PPUTLS46_015634	hypothetical protein	20.06	19.31	15.31	15.35	19.27	19.41	-3.89	0.01	11.57	0.95
PPUTLS46_005516	extracellular solute-binding protein	20.05	20.38	20.76	20.17	19.53	19.44	0.08	-0.06	1.12	1.2
PPUTLS46_022741	histidinol-phosphate aminotransferase	20.05	19.12	17.35	18.14	19.38	17.25	-1.02	-0.18	2.11	0.85
PPUTLS46_024273	organic solvent tolerance protein	20.05	19.81	20.27	20.1	18.8	19.79	0.09	-0.16	1.41	0.75
PPUTLS46_006026	TolC family type I secretion outer membrane	20.04	19.82	20.86	20.59	21.44	21.58	0.67	1.52	6.88	7.99
PPUTLS46_024278	Chaperone surA	20.04	20.45	20.14	19.68	19.36	19.23	-0.05	-0.22	1.18	1.48
PPUTLS46_002842	glutaminyl-tRNA ligase	20.04	19.38	19.88	18.47	19.32	19.99	-0.17	0.07	0.78	0.93
PPUTLS46_020811	nitroreductase	20.04	19.9	20.09	20.32	18.22	19.54	0.25	-0.01	0.53	0.96
PPUTLS46_022471	glucose-6-phosphate 1-dehydrogenase	20.04	18.05	19.71	19.41			0.1		0.92	1.51
PPUTLS46_015449	3-phosphoserine/phosphohydroxythreonine	20.03	19.95	18.62	18.93	19.77	19.81	-0.65	0.17	2.27	0.4
PPUTLS46_013998	protease Do	20.02	19.62	19.78	19.62	18.18	19.4	0	0	1.25	1.04
PPUTLS46_017099	hypothetical protein	20	20.44	17.04	17.07	19.76	19.16	-2.51	-0.13	5.44	1.27
PPUTLS46_009554	ATP-dependent protease La	19.99	20.67	20	20.07	20.23	19.11	-0.23	-0.07	0.64	1.1
PPUTLS46_018089	spermidine/putrescine ABC transporter ATPase	19.98	19.26	20.34	20.14	19.47	20.18	0.42	0.12	2.67	1.14
PPUTLS46_016459	alcohol dehydrogenase	19.98	18.63	21.23	21.46	18.91	18.33	1.36	-0.22	2.81	0.65
PPUTLS46_000540	aminopeptidase N	19.97	20.98	20.51	20.95	20.27	20.85	0.69	0.6	0.38	0.44
PPUTLS46_014384	phosphoenolpyruvate carboxylase	19.97	18.87	20.46	20.43	19.42	18.76	0.6	-0.45	2.14	0.19
PPUTLS46_005646	aspartate carbamoyltransferase catalytic subunit	19.97	17.13	19.31	19.35	18.86	19.44	0.04	0.04	1.01	1
PPUTLS46_012750	hypothetical protein	19.96	20.26	20.99	18.88	20.11	20.5	-0.21	0.16	0.84	0.76
PPUTLS46_013558	UDP-3-O-[3-hydroxymyristoyl] N-acetylglucosamine	19.96	19.13	19.87	18.86	19.5	19.6	-0.33	0.09	0.39	1.16
PPUTLS46_000390	isopropylmalate isomerase small subunit	19.96	20.78	20.16	19.63	19.41	20.61	-0.07	-1.05	1.14	0.1
PPUTLS46_010169	alkylhydroperoxidase-like protein	19.95	17.02	20.4	19.59	18.58	17.24	0.17	-1.16	1.08	0.36
PPUTLS46_006926	alcohol dehydrogenase	19.94	18.82	19.65	19.39	18.93	19.17	0.09	-0.01	0.87	0.93
PPUTLS46_022986	NADH:flavin oxidoreductase	19.94	19.26			17.15	17.5		-1.49	14.88	4.93
PPUTLS46_001522	glutamate dehydrogenase	19.93	19.22			20.65	20.34		0.87	13.3	4.38
PPUTLS46_024823	glycine betaine/L-proline ABC transporter,	19.93	19.07	21.91	21.57	19.29	17.04	1.91	-0.14	7.48	0.88
PPUTLS46_001147	hypothetical protein	19.92	20.28	19.79	19.86	19.52	20.45	-0.18	0.7	0.62	0.22
PPUTLS46_018084	extracellular solute-binding protein	19.92	19.92	20.61	20.43	19.27	18.54	0.34	-0.37	2.23	1.87
PPUTLS46_003952	preprotein translocase subunit YajC	19.9	20.23	19.26	19.03	20.65	19.83	-0.5	0.15	2.2	1.19
PPUTLS46_013573	bifunctional ornithine	19.9	18.4	18.23	19.06	19.82	19.46	-0.05	0.08	1.07	1.07
PPUTLS46_005581	ubiquinone/menaquinone biosynthesis	19.9	19.86	20.2	19.71	19.47	19.02	0.05	-0.15	1.1	1.59
PPUTLS46_009544	ATP-dependent Clp protease proteolytic subunit	19.89	20.12	21.41	20.19	19.62	20.8	0.3	0.46	1.34	0.56
PPUTLS46_006221	ribonuclease R	19.89	19.52	19.97	19.52	19.61	19.8	0.04	0.08	0.88	1.24
PPUTLS46_024613	methionine sulfoxide reductase A	19.89	17.32	19.05	18.79	19.04	19.09	0.25	0.19	0.84	0.89
PPUTLS46_019651	lipid-binding START domain-containing protein	19.88	19.2	19.22	19.73	22.77	21.67	-0.11	2.11	0.79	4.09
PPUTLS46_014089	glucose dehydrogenase	19.88	18.08	21.38	20.61	20.48	20.74	1.08	1.02	2.13	2.01

PPUTLS46_015854	enoyl-CoA hydratase	19.87	18.63	18.07	18.05	21.7	20.53	-0.71	1.12	2.27	2.22
PPUTLS46_004534	VacJ family lipoprotein	19.87	19.46	21.35	20.54	19.36	19.91	0.89	0.1	3.25	0.84
PPUTLS46_001677	competence lipoprotein ComL	19.87	19.56	20.46	19.08	18.13	18.19	0.3	-1.04	0.67	8.62
PPUTLS46_007161	triosephosphate isomerase	19.87	18.92	17.92	17.73	17.35	19.26	-1.2	0.03	4.36	1
PPUTLS46_017114	DNA-directed RNA polymerase subunit omega	19.87	20.67	20.18	16.47	17.21	19.08	-0.11	-0.2	1.05	1.15
PPUTLS46_004404	3-ketoacyl-CoA thiolase	19.86	19.52	18.57	18.71	24.71	24.71	-0.67	4.84	2.86	50.92
PPUTLS46_016579	DNA gyrase subunit B	19.84	19.07	19.74	18.96	19.26	18.58	-0.25	-0.14	0.33	0.62
PPUTLS46_024313	Uncharacterized conserved protein	19.82	17.6	19.64	18.95	19.35	19.26	0.3	0.06	0.77	0.97
PPUTLS46_011905	seryl-tRNA ligase	19.81	18.87	20.36	20.26	18.87	18.79	0.61	0.02	2.52	0.96
PPUTLS46_014799	arsenate reductase	19.81	18.88	19.87	19.48	17.69	18.73	0.06	-0.23	1.09	1.3
PPUTLS46_014454	cold-shock DNA-binding domain-containing protein	19.81	19.38							82.88	47.5
PPUTLS46_017949	secretion protein HlyD family protein	19.8	19.65	20.8	20.58	19.87	19.54	0.78	0.14	5.61	1.56
PPUTLS46_005741	DEAD/DEAH box helicase	19.8	19.28	19.74	19.62	19.01	18.6	0.03	-0.22	1.03	2.19
PPUTLS46_022771	toluene tolerance family protein	19.79	19.87	20.12	19.42	20.13	19.87	0.01	0.21	1.05	1.57
PPUTLS46_005206	50S ribosomal protein L31	19.79	17.84	20.08	19.06	19.32	19.54	0.32	0.18	0.74	1.14
PPUTLS46_001017	FKBP-type peptidylprolyl isomerase	19.78	20.87	19.66	18.01	20.56	20.71	-0.59	0.24	1.46	0.78
PPUTLS46_003852	4-hydroxy-3-methylbut-2-en-1-yl diphosphate	19.78	18.53	18.28	18.28	19.53	17.51	-0.43	-0.74	1.78	0.44
PPUTLS46_014894	phosphoribosylaminoimidazole synthetase	19.78	19.15	19.32	18.96	18.02	17.84	-0.18	-0.99	2.34	6.99
PPUTLS46_023928	50S ribosomal protein L30	19.77	21.89	21.3	20.59	21.41	21.65	0.16	0.35	0.92	0.81
PPUTLS46_004389	UspA domain-containing protein	19.76	17.46	20.66	20.24	20.53	19.62	0.81	0.37	1.67	1.24
PPUTLS46_011940	translation initiation factor IF-1	19.76	20.78	18.9	19.78	19.51	20.45	-0.31	-0.96	0.73	0.14
PPUTLS46_003832	GTP-binding protein Der	19.76	18.3	20.14	18.78	17.22	18.43	0.69	-0.23	0.37	1.22
PPUTLS46_015454	chorismate mutase	19.75	17.7	19.42	18.74	17.35	18.71	0.4	0.01	0.66	1.01
PPUTLS46_018461	FAD-dependent pyridine nucleotide-disulfide	19.75	19.67	17.98	18.63	17.03	18.37	-0.61	-0.66	1.8	1.69
PPUTLS46_022086	methionyl-tRNA ligase	19.74	18.29	18.65	18.74	18.57	17.94	-0.03	-0.23	1.09	0.67
PPUTLS46_024868	AsmA family protein	19.73	19.16	19.82	19.53	18.4	18.76	0.12	-0.25	1.8	1.78
PPUTLS46_009274	acetolactate synthase	19.72	17.63	19.57	18.82	17.15	18.34	0.35	-0.06	0.7	1.06
PPUTLS46_011955	cold-shock DNA-binding domain-containing protein	19.71	17.05	18.96	18.26	19.81	19.16	0.51	0.04	0.66	0.99
PPUTLS46_025383	uroporphyrin-III C-methyltransferase	19.71	19.61	20.99	20.68	19.19	19.11	0.98	-0.06	6.56	1.52
PPUTLS46_025393	porphobilinogen deaminase	19.71	19.28	17.92	19.35	18.4	19.34	-0.12	-0.08	0.89	0.85
PPUTLS46_016614	membrane protein insertase	19.7	18.89	19.31	19.33	19.06	19.2	0.01	0.05	0.95	0.95
PPUTLS46_005351	carboxyl-terminal protease	19.69	19.61	20.14	19.61	19	18.78	0.11	-0.29	1.35	2.63
PPUTLS46_012535	NADPH-dependent FMN reductase	19.68	20.1	20.26	20.02	20.38	20.92	0.1	0.29	0.85	1.38
PPUTLS46_003837	Pyrrolo-quinoline quinone	19.68	19.81	19.63	19.49	20.02	20.53	-0.05	0.22	0.76	1.35
PPUTLS46_013373	sulfate adenylyltransferase subunit 2	19.67	19.57	18.28	18.54	18.06	18.66	-0.69	-0.4	2.45	1.82
PPUTLS46_014864	hypothetical protein	19.66	18.89	22.74	21.67	21.26	20.06	2.23	0.83	4.49	2.06
PPUTLS46_021246	nitroreductase	19.66	16.85	18.58	17.93	20.03	18.75	-0.46	0.37	0.67	0.81
PPUTLS46_024208	anthranilate synthase component I	19.66	19.38	19.54	19.1	19.62	20.27	-0.11	0.21	2.41	1.34
PPUTLS46_016519	glycyl-tRNA ligase subunit alpha	19.66	19.8	19.35	18.75	18.93	18.81	-0.38	-0.25	2.06	1.82
PPUTLS46_017769	hypothetical protein	19.63	19.28	19.09	18.89	19.88	19.71	-0.34	0.56	4.49	22.83
PPUTLS46_010509	monosaccharide-transporting ATPase	19.63	19.47	19.9	19.86	17.7	17.25	0.06	-1.44	1.12	6.91
PPUTLS46_022236	ABC transporter-like protein	19.62	18	20.91	19.5	18.07	18.57	0.22	-0.01	1.16	0.96
PPUTLS46_009714	methylcitrate synthase	19.61	19.51	20.24	19.81	18.89	19.33	0.32	-0.17	2.52	0.52
PPUTLS46_017844	diaminopimelate decarboxylase	19.6	18.11	18.18	16.97	17.94	18.28	-0.28	-0.02	1.32	1.06
PPUTLS46_023268	methylmalonate-semialdehyde dehydrogenase	19.59	19.93	17.22	15.28	18.63	18.01	-2.22	-0.65	2.67	2.21
PPUTLS46_014874	glucose-methanol-choline oxidoreductase	19.58	18.56	21.82	20.74	20.75	20.52	1.45	1.33	3.01	4.02
PPUTLS46_010594	integration host factor subunit alpha	19.58	19.14	19.76	19.2	17.68	19	0.08	0.03	0.72	1
PPUTLS46_012475	NADH dehydrogenase subunit I	19.57	19.57	18.46	18.71	19.07	19.19	-0.45	-0.09	1.93	0.61
PPUTLS46_005251	3-dehydroquinate synthase	19.57	18.41	17.22	17.55	18.26	18.49	-1.01	-0.01	2.47	1.07
PPUTLS46_015924	3-ketoacyl-(acyl-carrier-protein) reductase	19.56	19.48	19.1	18.77	19.96	19.91	-0.41	0.47	3.35	3.19
PPUTLS46_005166	pyrroline-5-carboxylate reductase	19.55	19.57	18.85	18.58	19.59	19.47	-0.58	0.05	3.5	1.08
PPUTLS46_013403	tryptophanyl-tRNA ligase	19.55	18.72	18.05	19.01	18.72	17.72	0.02	0.03	0.99	1.01
PPUTLS46_002027	aromatic amino acid aminotransferase	19.54	18.91	19.45	19.31	19.16	17.42	0.05	-0.14	1.1	0.85
PPUTLS46_020266	chemotaxis protein CheW	19.54	19.17	20.48	20	17.85	20.23	0.75	-0.5	7.08	0.69
PPUTLS46_017929	alcohol dehydrogenase	19.53	18.81	18.55	17.85	19.67	18.66	-0.58	0.31	2.71	0.56
PPUTLS46_015389	Uncharacterized protein conserved in bacteria	19.53	19.04	20.71	20.38	19.21	18.24	1.16	-0.07	18.19	0.82
PPUTLS46_004479	glycerophosphoryl diester phosphodiesterase	19.53	16.79	20.31	18.53	19.18	18.43	0.6	0.47	0.69	0.73
PPUTLS46_009004	Gluconate 2-dehydrogenase subunit 3	19.52	17.22	19.41	19.23			0.18		1.13	0.96
PPUTLS46_001792	AAA domain	19.51	17.87	18.33	19.72	18.63	19.43	0.14	0.16	0.93	1.12
PPUTLS46_006396	DnaJ family curved-DNA-binding protein	19.5	19.91	20.53	19.58	19.72	18.47	0.11	-0.04	1.12	1.07
PPUTLS46_004259	Uncharacterized conserved protein	19.5	19.54	20.95	19.97	17.67	17.55	0.5	-1.27	1.82	5.79
PPUTLS46_023141	PfpI family intracellular peptidase	19.49	17.74	21.08	20.61	19.2	19.31	1.4	0.24	2.63	1.22
PPUTLS46_015544	glucose-1-phosphate thymidylyltransferase	19.49	19.27	20.24	20.49	19.13	19.41	0.51	0.16	2.01	0.56
PPUTLS46_023728	thioredoxin	19.49	17.94	18.96	18.72	18.56	16.56	0.15	-0.51	0.85	0.63
PPUTLS46_024233	extracellular solute-binding protein	19.49	20.01	22.05	21.21	18.11	17.51	1.29	-1.02	2.87	2.6
PPUTLS46_010189	alcohol dehydrogenase	19.49	18.7							9.02	5.31
PPUTLS46_019901	cytochrome c oxidase cbb3 type, accessory	19.48	20.37	20.29	19.79	20.34	19.48	0.09	0.04	0.92	1.01
PPUTLS46_022751	ATP phosphoribosyltransferase catalytic subunit	19.48	19.22	19.23	18.83	19.81	19.78	-0.23	0.57	3.97	6.22
PPUTLS46_025103	Hsp33-like chaperonin	19.48	18.78	18.42	17.1	17.37	17.71	-0.55	-0.87	1.73	3.31
PPUTLS46_003947	preprotein translocase subunit SecD	19.46	20.1	19.66	19.71	18.95	19.47	-0.31	-0.42	0.5	0.44
PPUTLS46_009564	PpiC-type peptidyl-prolyl cis-trans isomerase	19.46	19.2	20.05	20.56	18.87	18.27	0.33	-0.2	1.45	1.73
PPUTLS46_017894	polyphosphate kinase	19.46	19.89	20.17	19.97	17.95	18.6	0.03	-0.3	0.99	1.44
PPUTLS46_023408	alpha-2-macroglobulin domain-containing protein	19.45	19.34	20.58	19.38	18.05	18.64	0.11	-0.22	1.12	1.48
PPUTLS46_000700	acyl-CoA dehydrogenase domain-containing protein	19.45	18.34	16.98	16.48			-1.66		4.63	2.71
PPUTLS46_012430	isocitrate lyase	19.44	19.9			26.33	26.22		5.87	5.49	11.7
PPUTLS46_021001	peptidyl-prolyl cis-trans isomerase A	19.43	19.27	20.12	19.86	20.43	19.6	0.49	0.5	4.27	1.99
PPUTLS46_007106	chaperone protein DnaJ	19.43	19.36	18	18.55	19.86	20.36	-0.41	0.48	1.61	1.96
PPUTLS46_007509	isochorismatase hydrolase	19.42	20.22	21.01	19.45	19.45	20.02	0.08	0.65	1.04	0.31
PPUTLS46_021021	3-oxoacyl-(acyl carrier protein) synthase III	19.41	18.96	19.7	18.27	20.52	19.79	-0.28	0.8	0.64	2.96
PPUTLS46_000345	glutamyl-tRNA ligase	19.41	19.38	19.35	19.86	17.94	20.17	0.45	-0.64	0.41	0.58
PPUTLS46_002847	cysteinyl-tRNA ligase	19.4	18.59	19.17	18.84	19.47	18.68	-0.03	0.22	0.76	0.63
PPUTLS46_017879	isoprenoid biosynthesis protein	19.4	18.02	20.28	19.19	19.45	19.2	0.15	0.28	1.13	1.37
PPUTLS46_004414	DNA topoisomerase I subunit omega	19.4	19.66	19.86	19.83	18.96	18.56	0.16	-0.18	0.73	1.47
PPUTLS46_002887	delta-aminolevulinic acid dehydratase	19.4	18.78	18.64	19.59	18.82	18.1	-0.27	0.01	0.71	0.95
PPUTLS46_004896	ribose-5-phosphate isomerase A	19.37	18.18	18.72	18.49	18.61	17.88	0.01	-0.37	0.96	0.4

PPUTLS46_014884	Protein of unknown function (DUF3108)	19.37	19.05	19.42	19.3	18.5	19.53	0.07	0.31	0.78	0.65
PPUTLS46_007336	Uncharacterized enzyme of heme biosynthesis	19.36	18.28	18.19	18.33	19.29	18.95	-0.21	0.11	1.41	1.17
PPUTLS46_024108	tyrosyl-tRNA ligase	19.36	18.31	19.59	19.19	18.74	17.9	0.2	-0.38	1.41	0.37
PPUTLS46_023918	preprotein translocase subunit SecY	19.36	17.32	16.26	16.65	18.07	17.21	-1.01	-0.57	1.9	0.49
PPUTLS46_015374	polyphosphate:AMP phosphotransferase	19.35	19.42	20.6	20.42	18.68	18.56	0.81	-0.21	3.61	1.82
PPUTLS46_008564	CzcA family heavy metal efflux protein	19.35	18.77	17.73	18.12	16.08	17.19	-0.6	-1.23	2.08	2.49
PPUTLS46_019306	YVTN beta-propeller repeat-containing protein	19.35	19.03	19.98	19.88			0.54		3.7	45.75
PPUTLS46_022876	peptidase U62, modulator of DNA gyrase	19.34	17.73	18.75	18.05	18.09	18.54	-0.33	0.09	0.55	0.93
PPUTLS46_019256	Pyrrolo-quinoline quinone	19.34	17.7	19.31	19.18			0.21		1.22	1.33
PPUTLS46_016199	Protein of unknown function (DUF2782)	19.33	19.23	19.87	19.84	19.53	20.25	0.27	0.29	1.82	1.43
PPUTLS46_023041	imelysin. Metallo peptidase. MEROPS family M75	19.33	18.09	19.66	18.58	18.97	18.36	0.45	0.42	0.47	0.4
PPUTLS46_005431	glycogen phosphorylase	19.33	19.56	22.62	22.09	18.82	18.56	2.47	-0.16	7.1	1.48
PPUTLS46_005381	preprotein translocase subunit SecB	19.33	18.99	19.55	19.31	17.95	18.61	0.19	-0.1	2.97	1.25
PPUTLS46_022806	lipopolysaccharide transport periplasmic protein	19.32	19.69	19.57	19.17	20.32	20.08	0.02	0.5	0.97	1.97
PPUTLS46_002267	Short-chain dehydrogenase/reductase SDR	19.32	19.32	17.82	16.17	18.72	17.9	-1.32	-0.33	2.26	1.7
PPUTLS46_005436	fructose-1,6-bisphosphatase	19.32	19.09	19.15	19.22	17.78	18.1	-0.18	-0.59	0.37	2.92
PPUTLS46_009724	putative AcnD-accessory protein PrpF	19.3	18.66	16.59	17.08	18.96	19.12	-1.49	0.15	3.27	1.45
PPUTLS46_022596	hypothetical protein	19.29	19.75	20.76	19.79	19.82	20.14	0.34	0.09	1.43	1.1
PPUTLS46_010429	short chain dehydrogenase	19.29	17.47	21.03	19.58	19.56	19.28	0.64	0.44	1.48	1.42
PPUTLS46_020126	sulfate ABC transporter, periplasmic	19.29	19.05	19.16	19.07	19.49	17.7	-0.08	-0.16	0.43	0.82
PPUTLS46_009869	peptidase M14, carboxypeptidase A	19.28	18.48	19.47	19.65	17.87	16.32	0.32	-0.5	1.6	1.53
PPUTLS46_007774	Protein of unknown function (DUF3313)	19.27	15.75	20.67	20.19	19.72	18.9	1.25	0.16	1.64	1.06
PPUTLS46_004474	soluble pyridine nucleotide transhydrogenase	19.27	19.99	19.61	19.18	17.75	18.83	0.01	-0.05	0.96	0.93
PPUTLS46_005256	sporulation domain-containing protein	19.26	19.32	18.45	18.68	19.35	19.22	-0.19	0.06	1.42	1.12
PPUTLS46_014859	Uncharacterized protein conserved in bacteria	19.26	18.94	18.49	17.93	18.45	18.07	-0.69	-0.38	4.85	3.44
PPUTLS46_005976	bifunctional heptose 7-phosphate kinase/heptose	19.25	17.11	18.39	18.8	18.6	18.13	0.06	0.43	1.03	0.66
PPUTLS46_004921	phosphoenolpyruvate-protein phosphotransferase	19.24	18.69	20.28	19.84	18.41	18.42	0.94	-0.12	7.68	1.99
PPUTLS46_016539	general substrate transporter	19.24	19.62	17.48	13.86			-1.48		1.57	3.67
PPUTLS46_021376	hypothetical protein	19.22	17.38	15.82	15.55	18.46	17.61	-1.77	-0.71	3.03	0.31
PPUTLS46_013508	UDP-N-acetylmuramoyl-tripeptide--D-alanyl-D-	19.21	18	19.75	18.72	18.74	18.6	0.2	0.11	0.78	0.87
PPUTLS46_015574	beta-ketoacyl synthase-like protein	19.21	19.49							6.21	4.05
PPUTLS46_015509	UDP-N-acetylglucosamine 2-epimerase	19.2	18.95	17.75	18.66	19.27	18.95	-0.02	0.19	1.05	2.11
PPUTLS46_007351	acetolactate synthase 3 regulatory subunit	19.2	18.62	18.3	17.85	19.22	17.27	-0.66	-0.32	5.42	0.71
PPUTLS46_005496	proline iminopeptidase	19.2	18.62	18.76	18.92	18.94	18.73	-0.03	0.06	0.84	1.21
PPUTLS46_009774	peptidase	19.18	19.85	18.2	16.75	19.29	19.64	-1.14	0.46	2.07	0.44
PPUTLS46_012833	3-hydroxyisobutyrate dehydrogenase	19.18	17.61	20.97	20.54	18.31	18.46	1.61	0.13	3.19	0.88
PPUTLS46_004294	aconitate hydratase	19.18	19.16	21.47	20.7	17.96	19.29	1.5	-0.41	4.59	0.56
PPUTLS46_020181	PhzF family phenazine biosynthesis protein	19.17	18.17	15.21	15.91	20.88	20.32	-2.24	1.56	3.53	4.08
PPUTLS46_014174	signal recognition particle protein	19.17	16.7	18.24	16.74	18.46	15.81	-0.91	-1.58	0.4	0.26
PPUTLS46_017029	Uncharacterized protein conserved in bacteria	19.17	16.64	20.14	18.84	17.27	15.85	0.11	-0.54	1.06	0.65
PPUTLS46_021161	AAA family ATPase	19.16	18.94	17.3	17.62	19.6	19.64	-1.04	0.64	3.06	4.88
PPUTLS46_025629	hypothetical protein	19.16	19.45	17.35	18.08	19.56	20.1	-0.62	0.14	1.7	1.17
PPUTLS46_005911	trifunctional transcriptional regulator/proline	19.16	19.76	20.43	19.79	18.09	18.32	0.27	-0.28	1.37	1.45
PPUTLS46_023698	bifunctional 3,4-dihydroxy-2-butanone	19.15	18.72	19.15	18.74	19.28	19.41	-0.01	0.48	0.45	3.39
PPUTLS46_020201	cytochrome c-type biogenesis protein	19.15	18.95	18.97	18.83	17.85	17.32	0.01	-0.85	0.9	4.06
PPUTLS46_009709	2-methylisocitrate lyase	19.15	15.71	18.43	18.45	17.6	18.06	0.04	0.27	1.01	0.88
PPUTLS46_007669	polysaccharide export protein	19.14	18.62	21.23	21.22	20.89	19.61	2.01	0.84	8.36	2.05
PPUTLS46_009824	response regulator receiver protein	19.14	16.61	16.87	16.85	17.41	17.49	-0.22	-0.3	1.19	0.77
PPUTLS46_015519	family 2 glycosyl transferase	19.13	18.42	19.07	18.83	19.04	19.52	0.04	0.4	1.07	1.79
PPUTLS46_005621	poly(R)-hydroxyalkanoic acid synthase, class II	19.12	18.9	21.4	21.05	20.92	20.67	2.06	1.8	21.47	15.18
PPUTLS46_015554	dTDP-D-glucose 4,6-dehydratase	19.1	16.33	20.08	19.03	19.33	18.28	0.4	0.28	1.23	0.85
PPUTLS46_003927	inositol-phosphate phosphatase	19.1	19	19.85	18.5	17.72	16.63	0.2	-0.94	0.8	2.48
PPUTLS46_016624	tRNA uridine 5-carboxymethylaminomethyl	19.1	18.33	15.82	17.37	17.72	17.39	-0.83	-0.52	1.58	2.89
PPUTLS46_019266	Sulfur oxidation protein SoxY/Sulphur oxidation	19.1	18.21	18.85	19.36			0.06		1.04	3.18
PPUTLS46_026246	beta-ketothiolase	19.08	17.58	19.19	18.21	20.98	20.52	0.49	1.78	0.46	3.27
PPUTLS46_003797	ornithine decarboxylase	19.08	18.12	18.97	18.57	17.91	17.94	0.11	-0.08	0.75	1.26
PPUTLS46_001167	uracil phosphoribosyltransferase	19.08	18.77	17.76	16.42	16.08	18.47	-1.04	0.01	2.34	1.01
PPUTLS46_021691	dienelactone hydrolase	19.06	19.15	21.03	20.88	18.87	18.94	1.49	0.22	5.5	0.42
PPUTLS46_003847	histidyl-tRNA ligase	19.05	18.67	18.78	18.06	18.92	18.12	-0.16	-0.08	1.59	0.76
PPUTLS46_020291	chemotaxis-specific protein-glutamate	19.05	18.15	19.24	18.06	18.42	18.93	0.6	0.09	0.23	1.12
PPUTLS46_018686	dihydrodipicolinate reductase	19.05	18.45	19.14	18.69	17.81	19.62	0.05	0.32	0.85	0.78
PPUTLS46_006286	NAD synthetase	19.03	19.74	20.66	20.32	21.25	20.01	0.55	0.73	1.79	1.69
PPUTLS46_021451	Uncharacterized protein conserved in bacteria	19.03	19.07	18.87	18.79	18.97	18.53	-0.03	0.01	0.81	1.05
PPUTLS46_022866	pmbA protein	19.03	19.05	18.68	19.07	17.24	19.21	-0.34	-0.48	0.39	0.65
PPUTLS46_012730	dienelactone hydrolase	19.02	19.29	19.13	18.89	18.55	19.1	-0.03	-0.52	0.83	0.11
PPUTLS46_005061	rhodanese domain-containing protein	19.02	19.22	19.26	19.57	18.14	19.22	0.39	-0.53	0.42	0.36
PPUTLS46_009194	nickel transport system substrate-binding	19.02	17.06	20.11	19.41			0.76		1.72	0.87
PPUTLS46_012100	6-phosphogluconate dehydrogenase-like protein	19.01	18.72	20.47	20.38	18.72	18.82	1.32	0.04	7.54	0.96
PPUTLS46_013533	UDP-N-acetylmuramate--L-alanine ligase	19.01	14.94	17.79	18.37	18.31	17.51	0.11	0.68	1.04	0.73
PPUTLS46_007236	glucose-6-phosphate isomerase	19.01	17.56	19.63	19.39	17.08	17.68	0.66	-0.12	1.95	1.18
PPUTLS46_013313	PhoH family protein	19	19.43	17.89	17.79	19.72	20.6	-0.83	0.3	2.65	1.31
PPUTLS46_021441	peptidase M48, Ste24p	19	18.85	18.25	18.5	18.68	17.94	-0.07	-0.08	1.22	1.25
PPUTLS46_020431	sigma54 specific transcriptional regulator, Fis	18.99	17.98	20.2	19.88	16.29	17.13	1.16	-0.77	3.96	2.04
PPUTLS46_025308	CBS domain-containing protein	18.98	17.19	20.44	19.3	19.45	18.41	0.68	0.08	1.6	0.95
PPUTLS46_008769	putative transcriptional regulator, MvaT P16	18.98	18.78	19.34	19.18	18.95	17.42	0.21	-0.05	2.01	0.91
PPUTLS46_007261	Putative two component, sigma54 specific,	18.98	18.46	18.83	18.08	17.09	14.91	0	-1.09	0.92	1.82
PPUTLS46_011460	ssDNA-binding protein	18.95	17.14	17.55	17.32	19.44	19.02	-0.07	0.51	1.12	1.5
PPUTLS46_003652	Rhs element Vgr protein	18.95	19.33	16.95	17.27	19.31	19.58	-1.28	0.05	2.91	0.97
PPUTLS46_021876	ATP-dependent helicase HepA	18.95	19.7	18.69	18.71	17.1	18.99	0.03	-0.48	1	0.67
PPUTLS46_013503	UDP-N-acetylmuramoylalanyl-D-glutamate--2,	18.94	18.52	20.36	20.31	20.74	19.43	1.33	0.83	6.81	2.02
PPUTLS46_010584	phenylalanyl-tRNA ligase subunit alpha	18.94	17.81	19.05	18.99	17.47	18.71	0.29	-0.05	1.52	0.93
PPUTLS46_020556	type IV pilus assembly PilZ	18.93	17.53	18.04	17.91	18.95	18.16	0.02	0.27	1.02	0.7
PPUTLS46_020296	CheA signal transduction histidine kinase	18.92	19.44	18.45	18.37	19.81	19.33	-0.24	0.24	1.46	1.34
PPUTLS46_026306	lactoylglutathione lyase	18.92	18.14	18.86	18.37	18.09	17.57	0.2	-0.01	0.4	1.11
PPUTLS46_025971	decarboxylase family protein	18.91	17.97	18.57	18.91	16.5	18.37	0.03	0	1.01	0.98

PPUTLS46_012878	gamma-glutamyltransferase	18.9	17.64	17.51	18.96			-0.19		0.85	1.4
PPUTLS46_024408	pyrroloquinoline quinone biosynthesis protein	18.9	19.13							5.88	3.59
PPUTLS46_020546	anti-sigma-28 factor, FlgM	18.88	18.37	17.59	17.53	20.74	18.16	-0.81	0.2	4.68	0.9
PPUTLS46_025614	surface adhesion protein	18.88	20.26	18.25	18.7	19.06	18.17	-0.01	-0.08	0.97	1.09
PPUTLS46_022141	dihydroorotase	18.88	19.27	19.06	19.05	18.9	17.15	-0.26	-0.14	0.45	1.15
PPUTLS46_022021	synthetase	18.88	18.17			18.33	19.24		0.1	8.33	1.09
PPUTLS46_015654	Glycosyltransferase	18.88	19.43			18.17	17.8		-0.38	4.1	1.65
PPUTLS46_011140	cytochrome c-type protein	18.88	16.95	17.27	17.87	18.08	18.79	-0.03	0.2	1.05	1.15
PPUTLS46_001442	GTPase CgtA	18.88	18.24	16.69	17.05	17.7	17.02	-1.14	-0.47	3.06	2.25
PPUTLS46_011920	lipoprotein chaperone	18.88	18.42	17.86	18.73	17.2	18.88	-0.21	-0.26	0.75	0.76
PPUTLS46_014529	methionine aminopeptidase	18.87	17.53	19.32	19.62	17.03	17.81	0.68	-0.07	1.84	1.11
PPUTLS46_005641	dihydroorotase	18.86	18.89	19.22	18.69	18.69	17.82	0.05	-0.07	1.09	1.18
PPUTLS46_025133	osmolarity response regulator	18.82	19.21	19.49	19.44	18.06	17.77	0.06	-0.38	0.93	1.8
PPUTLS46_009854	type 1 pili usher pathway chaperone CsuC	18.81	19.06	19.84	19.72	18.99	19.3	0.44	0.09	1.99	0.88
PPUTLS46_012130	Protein of unknown function (DUF2934)	18.81	18.96	19.54	20.54	17.91	17.41	0.08	-0.56	1.05	2.43
PPUTLS46_013778	RND family efflux transporter MFP subunit	18.8	20.06	18.85	19.41	19.7	19.41	-0.7	0.19	0.4	0.86
PPUTLS46_009899	Uncharacterized protein conserved in bacteria	18.79	17.58	17.47	16.94	16.61	16.82	-0.47	-0.61	1.94	2.07
PPUTLS46_017724	aerotaxis receptor Aer	18.78	17.5	18.95	18.41	18.66	17.86	0.05	0.41	1.03	0.49
PPUTLS46_005086	signal recognition particle-docking protein FtsY	18.77	17.79	15.83	18.22	17	17.84	-0.1	-0.11	0.94	1.19
PPUTLS46_007221	Uncharacterized protein involved in outer	18.76	17.87	19.4	18.97	18.48	16.77	0.54	-0.41	2.61	0.6
PPUTLS46_000225	signal peptide peptidase SppA, 36K type	18.76	16.62	18.07	18.15	18.39	18.48	0.05	0.18	0.98	1.13
PPUTLS46_006226	23S rRNA (guanosine-2'-O-) - methyltransferase	18.75	18.1	18.23	18.7	18.74	18.77	0.18	0.4	0.78	2.7
PPUTLS46_000055	Predicted ATPase (AAA+ superfamily)	18.75	18.63	18.14	17.41	16.4	17.37	-0.58	-0.7	2.75	1.96
PPUTLS46_014334	peptide chain release factor 2	18.74	18.08	18.35	16.64	19.35	16.99	-0.01	0.6	0.96	0.61
PPUTLS46_005506	glucan biosynthesis protein G	18.74	19.06	19.46	19.18	18.44	18.55	0.11	-0.19	1.19	0.55
PPUTLS46_005291	cobyrinic acid a,c-diamide synthase	18.74	17.04	19.53	18.83	17.92	18.07	0.47	0.11	1.51	0.91
PPUTLS46_007136	putative RNA-binding protein, YhbY family	18.73	17.24	19.12	18.59	18.59	16.41	0.26	-1.01	1.32	0.32
PPUTLS46_010559	hypothetical protein	18.72	17.08	17.57	18.94	20.46	18.57	0.12	0.35	0.94	1.22
PPUTLS46_022071	deoxycytidine triphosphate deaminase	18.72	18.95	17.07	17.89	19.83	19.62	-0.35	0.75	1.4	2.83
PPUTLS46_005036	phosphopantetheine adenylyltransferase	18.72	17.13	19.47	19.49	19	14.65	0.9	-1.27	2.1	0.56
PPUTLS46_022656	glycine cleavage system T protein	18.72	17.99	19.12	19.15	17.63	18.69	0.47	0.16	2.25	0.85
PPUTLS46_017174	bifunctional	18.72	18.21	15.02	15.98	16.3	16.86	-1.95	-1.06	2.94	3.29
PPUTLS46_023718	Uncharacterized protein conserved in bacteria	18.71	17.58	18.86	18.3	19.16	17.85	0.06	0.36	0.93	0.65
PPUTLS46_001142	malate:quinone oxidoreductase	18.71	16.28	17.82	18.3	17.63	19.06	0.08	0.31	1.04	1.16
PPUTLS46_024398	pyrroloquinoline quinone biosynthesis protein	18.71	17.92	18.05	18.88			0.23		0.79	2.76
PPUTLS46_019896	cytochrome c oxidase, cbb3-type subunit III	18.7	18.88	19.82	19.73	16.59	16.82	0.58	-1.24	2.42	3.92
PPUTLS46_019861	cbb3-type cytochrome c oxidase subunit I	18.68	18.99			19.2	18.62		0.11	4.74	1.16
PPUTLS46_020096	2-hydroxy-3-oxopropionate reductase	18.68	18.19			18.34	17.25		-0.06	24.31	0.85
PPUTLS46_025368	anti-RNA polymerase sigma 70 factor	18.67	19.38	19.53	18.76	19.04	19	0.05	0.22	1.02	0.74
PPUTLS46_009679	phosphoadenosine phosphosulfate reductase	18.67	16.76	18.31	18.76	17.36	16.25	0.25	-0.51	1.19	0.55
PPUTLS46_023438	Uncharacterized protein conserved in bacteria	18.65	19.42	20.05	19.94	17.95	18.18	0.33	0.01	1.43	0.98
PPUTLS46_021411	lipoprotein	18.64	20.1	19.57	19.08	19.97	19.77	-0.1	0.1	0.88	0.94
PPUTLS46_005671	glutathione synthetase	18.64	17.55	17.34	17.8	18.97	17.93	-0.11	0.22	1.19	0.75
PPUTLS46_001427	hypothetical protein	18.64	17.79			15.3	17.27		-0.47	4.87	1.34
PPUTLS46_023338	methyl-accepting chemotaxis sensory transducer	18.63	18.76	18.53	18.43	15.21	12.01	-0.05	-2.73	0.77	2.34
PPUTLS46_022916	asparaginyl/glutamyl-tRNA amidotransferase	18.62	19.5	17.82	17.18	19.47	18.77	-0.88	0.04	2.13	1.01
PPUTLS46_000877	surface adhesion protein	18.62	19.3	20.35	20.25	19.43	20.19	0.71	0.15	2.02	1.13
PPUTLS46_006091	HAMP domain-containing protein	18.61	17.22	17.64	16.64	18.8	18.11	0.01	0.04	0.98	1.02
PPUTLS46_020316	protein FleN	18.61	16.96	19.76	19.43	17.62	17.11	1.08	-0.42	2.35	0.49
PPUTLS46_009159	H-NS family protein MvaT	18.61	17.4	19.32	18.74	17.44	17.05	0.49	-0.02	1.84	0.91
PPUTLS46_004047	aminotransferase	18.61	17.28	17.01	16.75	16.99	17.36	-0.56	-0.07	2.03	1.14
PPUTLS46_005756	5,10-methylenetetrahydrofolate reductase	18.61	18.32	18.64	19.11	16.97	16.29	0.18	-1.12	0.78	4.17
PPUTLS46_024203	outer membrane autotransporter barrel domain	18.61	17.72	14.13	17.12			-0.46		1.19	1.91
PPUTLS46_016794	cyclopropane-fatty-acyl-phospholipid synthase	18.6	18.29	18.92	18.2	16.83	14.34	0.09	-1.1	0.81	1.72
PPUTLS46_000725	Protein of unknown function (DUF3144)	18.59	19.36	19.3	17.91	19.77	18.97	-0.02	0.2	1.05	1.2
PPUTLS46_014299	methyl-accepting chemotaxis sensory transducer	18.59	17.67	20.16	19.32	18.39	17.14	1.04	-0.5	2.96	0.37
PPUTLS46_005681	response regulator receiver protein	18.57	19.23	18.13	18.85	18.6	19.27	-0.58	0.59	0.41	0.35
PPUTLS46_023298	adenosine deaminase	18.56	15.12	19.17	18.24	19.15	17.72	0.23	0.3	1.1	0.88
PPUTLS46_000715	acetyl-CoA acetyltransferase	18.56	18.48	16.05	16.26			-1.84		5.03	4.9
PPUTLS46_017064	signal transduction protein	18.55	17.29	17.2	17.23	17.36	14.86	-0.3	-0.03	1.55	0.97
PPUTLS46_021981	OmpA/MotB domain-containing protein	18.54	18.46	20.48	19.92	18.88	20.02	1.43	0.37	6.36	1.39
PPUTLS46_025398	LytTR family two component transcriptional	18.54	17.84	20.16	20.3	18.11	17.49	1.59	-0.21	4.69	0.39
PPUTLS46_008559	RND family efflux transporter MFP subunit	18.54	19.37	16.88	16.62	15.28	15.66	-1.47	-2.14	3.03	3.53
PPUTLS46_021046	long-chain-fatty-acid--CoA ligase	18.53	18.49	17.71	16.82	19.16	18.32	-0.8	0.18	2.71	1.3
PPUTLS46_014849	(p)ppGpp synthetase I SpoT/RelA	18.53	18.09	20.33	20.09	17.95	15.99	1.77	-0.08	22.13	1.09
PPUTLS46_013608	MscS mechanosensitive ion channel	18.53	18.51	18.14	17.59	16.89	17.34	-0.42	-0.58	2.59	2.36
PPUTLS46_004012	selenophosphate synthetase	18.52	17.22	16.78	16.63	18.36	17.89	-0.67	0.13	2.22	0.85
PPUTLS46_025684	toxin secretion ATP-binding protein	18.51	18.79	18.88	19.27	18.33	18.15	0.34	-0.02	0.55	0.88
PPUTLS46_022081	ATPases involved in chromosome partitioning	18.51	17.58	17.73	14.91	17.35	18.27	-0.03	0.09	0.97	0.93
PPUTLS46_013418	aldo/keto reductase	18.5	17.29	19.61	19.39	18.21	17.87	1.1	0.13	3.1	0.83
PPUTLS46_020451	flagellar protein FlaG protein	18.49	18.65	17.05	16.15	17.56	16.74	-1.42	-0.64	3.6	2.19
PPUTLS46_017899	Exopolyphosphatase	18.49	17.61	18.99	19.03	17.38	15.12	0.59	-0.16	2.33	1.13
PPUTLS46_005326	betaine aldehyde dehydrogenase	18.49	17.9	17.67	16.7	17.19	17	-0.48	-0.61	1.98	5.41
PPUTLS46_019881	cbb3-type cytochrome c oxidase subunit I	18.48	18.33	19.33	19.03	18.63	18.07	0.62	0.08	5.21	1.16
PPUTLS46_001437	gamma-glutamyl kinase	18.47	17.95	15.86	17.82	19.3	18.43	0.02	0.42	1	1.79
PPUTLS46_004067	cytochrome o ubiquinol oxidase subunit I	18.46	18.29			19.44	19.61		1.1	12.29	5.35
PPUTLS46_023293	short chain dehydrogenase	18.46	18.85	19.96	19.46	19.03	18.86	0.66	0.16	2.3	1.26
PPUTLS46_017384	transcriptional regulator MvaT, P16 subunit, put	18.46	17.85	19.85	19.01	17.37	18.47	0.82	0.2	2.88	0.8
PPUTLS46_022526	glucokinase	18.46	17.28	18.1	16.82			-0.43		0.49	0.96
PPUTLS46_000585	Protein of unknown function (DUF2628)	18.45	20.75	17.94	17.63	19.74	19.27	-0.53	0.37	1.35	0.81
PPUTLS46_004329	lipoprotein	18.45	20.38	18.7	19.03	16.61	19.81	-0.48	-1.67	0.66	0.37
PPUTLS46_021886	3-hydroxyisobutyrate dehydrogenase	18.45	16.48	18.43	17.8	16.15	16.52	0.17	-0.13	0.86	1.14
PPUTLS46_009929	B12-dependent methionine synthase	18.43	17.5	17.62	17.24	18.01	18.02	-0.23	0.08	1.77	1.13
PPUTLS46_025826	acyl-CoA dehydrogenase-like protein	18.42	15.59	15.61	16.8	17	16.35	-0.16	-0.72	1.1	0.54
PPUTLS46_022486	Porin B	18.42	18.09	17.15	16.95			-1.06		10.92	24.96

PPUTLS46_013773	hydrophobe/amphiphile efflux-1 (HAE1) family	18.41	18.02	18.26	18.51	17.63	17.3	0.2	-0.32	0.65	3.67
PPUTLS46_008434	heat shock protein Hsp20	18.41	19.04			16.64	16.81		-0.95	3.11	2.44
PPUTLS46_000250	malonyl CoA-acyl carrier protein transacylase	18.41	14.43	18.89	18.33	16.31	15.74	0.47	-0.88	1.2	0.62
PPUTLS46_007534	peptidase M24	18.4	18.76	17.67	18.84	19.99	20.71	-0.74	1.17	0.39	2.53
PPUTLS46_021041	long-chain-fatty-acid--CoA ligase	18.36	18.67	18.28	18.34	21.77	21.47	-0.22	2.71	0.53	6.88
PPUTLS46_017449	aldehyde dehydrogenase	18.36	17.41	18.54	17.33	20.13	19.59	0.64	1.64	0.22	4.48
PPUTLS46_015744	GTP cyclohydrolase I	18.36	17.35	18.04	17.79	16.62	17.74	0.08	0.03	0.86	1
PPUTLS46_026281	multi-sensor hybrid histidine kinase	18.33	19.75	20.2	20.16	19.12	19.03	0.23	0.34	1.18	0.74
PPUTLS46_026211	glycolate oxidase iron-sulfur subunit	18.32	18.61	20.71	19.72			1.18		2.73	2.12
PPUTLS46_009674	transcriptional regulator CysB	18.31	18.71	20.01	19.75	18.96	17.34	0.92	-0.01	2.85	0.96
PPUTLS46_002522	AcdA	18.31	16.2	17.98	16.8	17.95	17.24	0.85	0.46	0.34	0.64
PPUTLS46_006756	acyl-CoA dehydrogenase	18.3	15.72	19.4	19.5	18.95	18.48	1.28	0.59	1.91	1.38
PPUTLS46_012160	Alpha amylase, catalytic region	18.3	17.81	20.71	19.47	18.31	18.63	1.3	0.39	2.75	2.11
PPUTLS46_021271	Protein of unknown function (DUF465)	18.29	18.48	15.79	18.73	18.8	19.04	-0.84	0.33	0.66	1.67
PPUTLS46_016664	F0F1 ATP synthase subunit delta	18.29	15.04	20.38	20.1	18.31	17.71	1.98	0.07	2.12	0.98
PPUTLS46_017109	ppGpp synthetase I SpoT/RelA	18.29	16.75	18.95	18.32	17.24	16.48	0.42	-0.41	1.52	0.5
PPUTLS46_021581	histidine triad	18.29	17.09	18.76	15.98	17.17	18.23	-1.1	0.03	0.43	1
PPUTLS46_018276	efflux transporter RND family, MFP subunit	18.29	17.05							1.85	0.81
PPUTLS46_003687	YD repeat-containing protein	18.28	18.14			19.28	19.25		1.08	9.5	7.36
PPUTLS46_020466	flagellar hook-associated protein FlgL	18.28	17.68	18.82	19.56	18.52	18.56	0.46	0.61	1.5	4.06
PPUTLS46_009844	spore coat U domain-containing protein	18.28	18.22	17.99	16.62	17.37	18.19	-0.28	-0.3	1.37	0.51
PPUTLS46_007679	lipopolysaccharide biosynthesis protein	18.26	18.3	21.13	20.96	20.2	19.34	2.41	1.18	9.36	3.1
PPUTLS46_013538	D-alanine--D-alanine ligase	18.26	17.82	18.06	17.05	18.29	18.19	-0.02	0.41	1.09	7.89
PPUTLS46_020016	GntR family transcriptional regulator	18.26	17.84	18.04	17.62	17.6	17.27	-0.14	-0.19	3.2	2.62
PPUTLS46_005221	penicillin-binding protein 1A	18.26	17.47	17.55	17.08	15.78	18.08	-0.28	-0.17	2.13	0.88
PPUTLS46_003887	chaperone protein HscA	18.26	17.75	16.71	16.11	15.26	16.89	-1.32	-0.56	6.88	1.5
PPUTLS46_015064	recombination associated protein	18.25	18.17	19.05	18.32	18.92	17.76	0.23	0.04	1.55	1.02
PPUTLS46_025689	HlyD family type I secretion membrane fusion	18.25	18.96	20.11	18.8	18.55	18.33	0.32	0.18	1.29	0.79
PPUTLS46_015424	oxoacyl-(acyl carrier protein) reductase	18.25	18.32	19	18.01	17.8	19.01	0.03	0.45	1	0.57
PPUTLS46_018281	transporter hydrophobe/amphiphile efflux-1	18.25	17.1							2.05	0.8
PPUTLS46_006081	DNA topoisomerase IV subunit A	18.24	17.25	18.68	18.73	17.54	17.92	0.56	0.06	2.12	1.06
PPUTLS46_005566	phosphoribosyl-AMP cyclohydrolase	18.24	17.12	17.27	17.27	17.18	13.68	-0.11	-0.02	1.28	0.98
PPUTLS46_026491	glutathione reductase	18.24	17.06	16.31	16.53	16.54	16.83	-0.71	-0.22	2.14	1.41
PPUTLS46_025303	dipeptidase, putative	18.23	17.97	18.64	18.58	20.06	18.83	0.27	0.88	2.05	2.17
PPUTLS46_013013	Uncharacterized protein conserved in bacteria	18.23	18.39	17.37	18.32	18.18	18.36	-0.43	0.21	0.57	0.56
PPUTLS46_015659	Polysaccharide export protein	18.22	17	17.31	17.66	17.1	17.03	0.03	-0.05	1	0.87
PPUTLS46_008579	Predicted metal-binding protein	18.22	16.71							1.29	0.77
PPUTLS46_015779	chorismate synthase	18.21	18.42	17.81	17.37	18.39	17.52	-0.41	0.01	2.18	1.04
PPUTLS46_013683	formyltetrahydrofolate deformylase	18.21	17.28	18.46	17.99	17.29	17.42	0.15	0.01	1.34	0.95
PPUTLS46_024593	bifunctional glutamine-synthetase	18.2	16.33	19.08	18.5	16.54	15.73	0.66	-0.16	1.68	0.83
PPUTLS46_023818	selenocysteine synthase	18.2	17.27	17.57	17.62	16.02	15.03	0.01	-1.13	1.03	2.72
PPUTLS46_009189	TonB-dependent siderophore receptor	18.19	17.52	19.32	20.32			0.98		1.89	1.78
PPUTLS46_008159	Dyp-type peroxidase family protein	18.18	17.57			17.87	17.65		0.06	7.7	0.84
PPUTLS46_013083	fumarylacetoacetase	18.18	15.78	19.26	19	14.67	16.96	1.08	-0.08	1.85	1.05
PPUTLS46_000145	acyl-CoA dehydrogenase	18.17	17.6	21.03	20.31	17.41	18.58	2.39	0.15	8.13	0.87
PPUTLS46_023388	two component LuxR family transcriptional	18.17	18.12	16.81	16.73	16.93	19.81	-1.05	0.73	4.82	0.65
PPUTLS46_014008	signal peptidase I	18.15	17.12	18.09	17.95	20.07	17.49	0.14	0.18	1.24	0.91
PPUTLS46_017074	LysR family transcriptional regulator	18.15	17.12	18.43	18.1	17.01	17.26	0.28	0.02	1.64	1.02
PPUTLS46_000265	3-oxoacyl-(acyl carrier protein) synthase II	18.13	18.51	16.68	17.84	18.27	18.45	-0.11	0.22	0.88	0.65
PPUTLS46_020376	Hpt protein	18.12	18.63	17.09	13.36	17.14	17.89	-0.93	-0.23	1.35	0.69
PPUTLS46_004289	methyl-accepting chemotaxis sensory transducer	18.12	18.08	18.03	19.3	16.96	16.65	0.53	-0.73	0.61	4.13
PPUTLS46_006761	AMP nucleosidase	18.11	16.88	16.85	17.23	18.42	17.79	-0.08	0.2	1.15	1.27
PPUTLS46_013488	16S rRNA m(4)C1402 methyltransferase	18.11	17.86	18.39	16.95	18.12	17.69	-0.16	0.08	0.79	1.23
PPUTLS46_000445	sporulation domain-containing protein	18.1	17.79	18.14	18.58	18.29	18.31	0.16	0.49	0.8	4.94
PPUTLS46_025751	gluconate 2-dehydrogenase acceptor subunit	18.1	18.09	19.43	19.05			0.91		4.88	2.3
PPUTLS46_016594	chromosomal replication initiation protein	18.09	17.85	17.54	16.24	18.45	17.06	-0.41	0.21	1.56	0.78
PPUTLS46_025741	2Fe-2S iron-sulfur cluster binding protein	18.09	17.16	20.03	18.59	17.68	17.4	0.73	0.17	1.74	0.63
PPUTLS46_016699	glucosamine--fructose-6-phosphate	18.08	19.58	18.69	18.84	20.22	20.82	-0.48	0.58	0.58	1.42
PPUTLS46_008989	hypothetical protein	18.08	16.82							1.5	0.79
PPUTLS46_023166	two component Fis family transcriptional	18.07	19.14	17.79	18.42	20.09	19.67	-0.56	0.7	0.49	1.7
PPUTLS46_012010	alpha/beta hydrolase fold family protein	18.06	18.15	20.99	20.32	18.5	17.97	2.12	0.18	6.31	1.36
PPUTLS46_020441	flagellar protein FliS	18.06	16.88			16.08	15.03		-0.71	1.64	1.92
PPUTLS46_024188	anthranilate phosphoribosyltransferase	18.05	18.23	18.86	18.18	16.91	18.11	0.17	-0.45	1.32	0.5
PPUTLS46_023688	transcription antitermination protein NusB	18.05	18.42	18.24	18.76	15.56	18.09	0.6	-0.44	0.29	0.75
PPUTLS46_006721	PhoH family protein	18.05	17.12	15.98	16.04	15.56	15.74	-1.14	-1.15	3.57	3.8
PPUTLS46_004791	sulfate ABC transporter, periplasmic	18.03	17.78	19.17	18.54	18.14	17.57	0.71	0.06	3.73	1.1
PPUTLS46_023423	arginine decarboxylase	18.03	18.64	19.66	18.58	17.83	16.98	0.32	-0.2	1.35	1.29
PPUTLS46_004821	NLPA lipoprotein	18.03	17.53	17.1	17.66	17.79	18.88	-0.04	0.19	0.9	1.19
PPUTLS46_023353	3-ketoacyl-(acyl-carrier-protein) reductase	18.03	17.26	20.06	19.57	17.73	15.62	1.84	-0.3	7.78	0.76
PPUTLS46_022826	PTS IIA-like nitrogen-regulatory protein PtsN	18.02	17.09	17.78	18.14	17.86	16.76	0.07	0.56	1.07	0.28
PPUTLS46_024218	ribulose-phosphate 3-epimerase	18.02	18.16	18.65	16.65	15.7	17.5	-0.17	0	0.86	0.98
PPUTLS46_018074	aminotransferase	18.02	17.89	18.48	16.42	14.24	18.12	-0.24	-0.44	0.8	0.83
PPUTLS46_025644	hypothetical protein	18.01	17.88	18.77	17.45	18.55	18.27	0.2	0.54	0.8	3.81
PPUTLS46_016194	DNA polymerase I	18.01	18.35	19.19	19.33	18.36	18.05	0.48	0.05	1.78	1.04
PPUTLS46_015139	Cro/CI family transcriptional regulator	18.01	17.62			17.59	16.42		0	150.67	1.04
PPUTLS46_013633	Putative heme iron utilization protein	18.01	17.47	15.68	15	17.53	18.49	-2.04	0.05	8.08	1.03
PPUTLS46_004754	protease	18	16.92			17.11	15.19		-0.13	1.78	0.88
PPUTLS46_016229	hypothetical protein	17.99	18.73	19.25	19.98	20.05	19.92	0.15	1.12	1.1	2.5
PPUTLS46_012888	Predicted metalloprotease	17.99	16.48	18.82	18.04	18.31	18.18	0.43	0.57	1.51	1.73
PPUTLS46_007719	hexapeptide repeat-containing transferase	17.97	16.97	19.35	17.97	18.65	19.3	0.28	1.13	1.27	2.65
PPUTLS46_013708	Predicted ATPase	17.96	18.4	19.42	19.18	17.23	18.04	0.66	-0.52	2.24	0.33
PPUTLS46_019906	Uncharacterized protein conserved in bacteria	17.93	17.62			18.48	18.98		0.76	24.14	2.71
PPUTLS46_019671	OmpA/MotB domain protein	17.93	17.26	17.95	17.97	17.53	16.19	0.15	-0.16	1.34	0.77
PPUTLS46_014294	Uncharacterized protein conserved in bacteria	17.93	19.42	15.98	16.79			-0.79		1.57	1.11
PPUTLS46_018306	ThiJ/PfpI domain protein	17.92	19.19	21.09	20.94	18.48	19.22	1.5	0.62	2.45	0.51

PPUTLS46_025058	adenosine nucleotide hydrolase NudE	17.92	17.38							8.49	1.39
PPUTLS46_012968	inner membrane protein yjiY	17.91	19.51	19.99	19.9	18.87	17.68	0.28	0.01	1.2	0.98
PPUTLS46_012710	3-hydroxydecanoyl-(acyl carrier protein)	17.91	16.78	19.6	19.04	16.71	18.41	1.44	0.08	3.89	0.96
PPUTLS46_013988	anti sigma-E protein, RseA	17.9	17.33	16.91	14.98	21.1	18.32	-0.48	0.71	1.4	1.39
PPUTLS46_020446	flagellar cap protein FliD	17.9	16.95	19.56	19.17	19.28	18.81	1.55	1.34	5.33	3.99
PPUTLS46_018316	4-hydroxyphenylpyruvate dioxygenase	17.9	17.5	18.49	19.03	17.72	16.59	0.42	-0.09	1.56	0.82
PPUTLS46_016009	NAD(P) transhydrogenase subunit alpha	17.89	18.62	18.85	18	21.35	21.36	0.06	2.45	1.04	4.32
PPUTLS46_001027	phosphate acetyltransferase	17.88	17.12	16.19	16.66	16.92	16.87	-0.52	-0.09	1.82	1.43
PPUTLS46_017144	TIGR00255 family protein	17.88	16.86	18.71	18.11			0.58		2.24	0.89
PPUTLS46_023478	ATP-NAD/AcoX kinase	17.88	17.29	15.9	14.34			-1.46		2.52	1.03
PPUTLS46_014329	response regulator receiver modulated	17.87	16.17	17.31	16.39	17.53	16.11	-0.5	1.2	0.43	0.04
PPUTLS46_001187	NAD/FAD-dependent oxidoreductase-like protein	17.86	17.09			18.43	17.37		0.04	2.96	1.02
PPUTLS46_000030	heat shock protein HtpX	17.86	17.48	17.25	18.11	16.68	16.33	-0.34	-0.68	0.6	6.04
PPUTLS46_009659	phospho-2-dehydro-3-deoxyheptonate aldolase	17.85	17.88	18.76	18.29	18.24	17.6	0.44	0.12	2.47	1.21
PPUTLS46_003757	peptide chain release factor 3	17.85	16.66	18.02	16.85	16.26	15.04	0.71	-0.34	0.18	1.41
PPUTLS46_006356	Ferritin, Dps family protein	17.85	16.55	16.6	16.85			-0.12		1.22	0.93
PPUTLS46_012948	large-conductance mechanosensitive channel	17.84	18.36	18.94	18.18	18.17	17.28	0.18	0.01	1.23	1.03
PPUTLS46_017554	aldehyde dehydrogenase family protein	17.82	17.62	17.76	17.82	17.33	18.01	0.27	0.25	0.27	0.62
PPUTLS46_009999	Uncharacterized conserved protein	17.82	16.33							0.97	0.97
PPUTLS46_001032	OmpA/MotB domain-containing protein	17.81	19.13	18.39	18.36	18.15	17.9	-0.32	-0.21	0.66	0.79
PPUTLS46_020361	flagellar motor switch protein FliM	17.8	17.43	18.19	18.13	17.98	18.04	0.32	0.5	2.36	4.56
PPUTLS46_016104	ABC-type uncharacterized transport system	17.8	17.51	17.83	17.04	17.44	16.53	0.03	-0.03	1	1.12
PPUTLS46_009529	bifunctional 5,10-methylene-tetrahydrofolate	17.79	17.71	18.24	18.28	17.51	18.7	0.16	0.2	1.36	0.81
PPUTLS46_021506	biopolymer transport protein TolQ	17.79	18.01	18.41	18.3	17.33	16.87	0.08	-0.21	1.13	1.53
PPUTLS46_010604	diguanylate cyclase	17.78	18.48	19.03	18.79	16.93	18.14	0.26	-0.78	1.35	0.29
PPUTLS46_012690	NAD(P)H-dependent glycerol-3-phosphate	17.77	17.85	16.75	17.84	18.35	17.41	-0.43	0.09	0.6	1.1
PPUTLS46_023873	excinuclease ABC subunit A	17.77	17.14	18.51	18.67	17.23	16.36	0.73	-0.04	2.69	0.87
PPUTLS46_009949	electron transport protein SCO1/SenC	17.76	17.25	16.81	17.27	18.3	16.99	0	0.21	1.05	0.78
PPUTLS46_005191	sporulation domain protein	17.76	17.76	17.48	15.74	17.87	17.26	-0.27	0.05	1.26	1.04
PPUTLS46_005796	Protein of unknown function (DUF1090)	17.75	18.86	18.91	18.62	18.97	19.72	0.09	0.13	0.93	1.09
PPUTLS46_023328	heavy metal translocating P-type ATPase	17.75	17.87	19.88	19.23	18.77	17.74	1.36	0.28	4.37	1.36
PPUTLS46_006151	tRNA threonylcarbamoyl adenosine modification	17.75	18.05	16.97	17.13	17.65	18.17	-0.26	0.41	1.52	0.4
PPUTLS46_020646	succinylglutamate desuccinylase	17.75	17.74	19.14	17.86	15.76	15.55	0.22	-1.46	1.24	7.18
PPUTLS46_012155	trehalose synthase	17.74	17.6	21.49	20.54	18.14	18.47	2.78	0.51	6.37	2.38
PPUTLS46_000977	nicotinate-nucleotide pyrophosphorylase	17.73	18.9	18.99	18.24	18.33	17.71	0.04	-0.07	1.01	0.91
PPUTLS46_007011	Uncharacterized conserved protein	17.73	16.85			17.29	16.68		-0.39	1.88	0.09
PPUTLS46_019301	ABC-type branched-chain amino acid transport	17.73	17.88	19.03	19.35			0.75		2.18	0.97
PPUTLS46_002082	phosphoglucomutase	17.72	18.16	20.44	19.67	18.34	19	1.55	0.19	3.51	1.21
PPUTLS46_006066	DNA topoisomerase IV subunit B	17.72	16.84	18.29	17.91	17.63	16.29	0.52	-0.5	2.59	0.4
PPUTLS46_012680	phosphohistidine phosphatase, SixA	17.72	17.85	16.83	17.55	17.21	17.36	-0.2	-0.09	0.75	0.68
PPUTLS46_011135	cytochrome c, class I	17.72	16.7	17.47	18.32	16.25	17.05	0.08	0.02	1.06	1.02
PPUTLS46_020666	arginine N-succinyltransferase	17.72	18.11	17.85	17.79			-0.21		0.54	0.96
PPUTLS46_007196	30S ribosomal protein S15	17.7	18.5	16.62	17.04	15.61	17.43	-0.32	-0.21	1.38	0.84
PPUTLS46_014804	alkyl hydroperoxide reductase/ Thiol specific	17.69	18.72	19.24	18.65	18.51	17.23	0.24	0.01	1.23	1.01
PPUTLS46_011975	tRNA-specific 2-thiouridylase MnmA	17.69	18.5	16.66	15.73	16.97	15.96	-1.17	-0.62	2.39	1.67
PPUTLS46_010849	Uncharacterized conserved protein	17.69	17.23	15.07	16.32	16.07	17.51	-0.66	-0.18	1.55	0.8
PPUTLS46_025483	hypothetical protein	17.69	16.43	18.08	17.52	12.87	17.58	0.22	-0.25	1.34	0.91
PPUTLS46_004459	transcription-repair coupling factor	17.68	17.29	18.28	18.12	16.7	16.84	0.57	-0.23	5.13	2.35
PPUTLS46_013993	sigma E regulatory protein MucB/RseB	17.67	18.04	18.29	17.57	18.27	16.64	0.05	-0.01	1.04	0.96
PPUTLS46_006776	asnC family transcriptional regulator	17.67	17.28	17.84	17.36	16.92	16.49	0.04	-0.28	1.06	2.58
PPUTLS46_025223	serine O-acetyltransferase	17.66	19.32	19.05	18.16	18.58	15.48	0.07	-0.21	0.97	1.1
PPUTLS46_006991	EcoEI R domain-containing protein	17.66	18.03	13.79	16.18	18.26	16.21	-0.75	-0.03	1.35	0.95
PPUTLS46_018059	N-acetylglutamate synthase	17.66	17.33	17.61	18.01	17.62	16.59	0.19	-0.09	0.72	0.8
PPUTLS46_023543	ethanolamine ammonia lyase large subunit	17.65	15.7	15.87	16.15			-0.16		1.18	1.09
PPUTLS46_022811	ABC transporter related protein	17.63	17.73	18.35	17.76	18.71	18.27	0.19	0.71	1.45	2.85
PPUTLS46_000695	3-hydroxybutyryl-CoA epimerase	17.63	18.21							1.98	0.88
PPUTLS46_023643	1-deoxy-D-xylulose-5-phosphate synthase	17.62	16.22	14.45	16.1			-0.49		1.31	1.16
PPUTLS46_006216	iron ABC transporter, periplasmic iron-binding	17.59	17.43	18.41	17.57	16.83	16.09	0.19	-0.4	1.37	2.02
PPUTLS46_015524	type 11 methyltransferase	17.57	16.37	18.4	18.29	17.48	17.66	0.9	0.41	2.62	1.64
PPUTLS46_003557	type VI secretion protein, VC_A0107 family	17.56	18.91	18.29	17.42	17.05	17.3	-0.01	-0.13	1.04	0.86
PPUTLS46_003957	queuine tRNA-ribosyltransferase	17.55	19.26	17.26	16.97	18.61	19.08	-0.31	0.5	1.27	0.66
PPUTLS46_021381	Surface antigen	17.55	17.34			17.43	16.5		0	5.97	0.95
PPUTLS46_016639	chromosome-partitioning protein parB	17.55	17.27	18.8	18.62	17.33	18.25	1.14	0.06	9.37	1.04
PPUTLS46_000460	O-succinylhomoserine sulfhydrylase	17.53	19.36	18.98	18.03	17.99	18.31	0.07	-0.61	0.97	0.56
PPUTLS46_005891	Predicted Zn-dependent proteases and their	17.52	18.17			17.31	17.12		-0.01	1.78	0.94
PPUTLS46_022831	glmZ(sRNA)-inactivating NTPase	17.5	17.04	16.38	17.19	17.08	16.83	-0.09	0	0.86	0.65
PPUTLS46_023273	beta alanine--pyruvate transaminase	17.5	15.9			16.32	14.49		-0.35	0.87	0.73
PPUTLS46_014739	Domain of unknown function (DUF4146)	17.5	18.01	19.03	17.48	16.02	17.95	0.07	-0.75	1.04	0.48
PPUTLS46_000235	Predicted metal-binding, possibly nucleic	17.49	14.86			18.09	18.39		1.04	0.94	1.67
PPUTLS46_010734	O-acetylhomoserine aminocarboxypropyltransferase	17.49	19.73	19.85	19.79	17.93	18.84	0.07	1.12	1.02	0.41
PPUTLS46_001082	glycerate dehydrogenase	17.49	17.87	17.55	14.83	16.63	16.98	-0.14	0.02	1.09	1.01
PPUTLS46_005636	ATP-dependent protease subunit HslV	17.49	16.93	15.36	15.15	15.52	16.08	-1.76	-0.62	13.39	2.37
PPUTLS46_000495	NADH:flavin oxidoreductase	17.48	15.85	16.04	16.68	22.76	22.47	-0.01	5	1.03	7.01
PPUTLS46_020621	magnesium transporter	17.47	15.97	17.42	17.04			0.04		0.99	1.28
PPUTLS46_021101	CsbD family protein	17.45	16.51							1.08	1.63
PPUTLS46_015999	glutaryl-CoA dehydrogenase	17.44	17.3	17.14	15.76	17.39	17.84	-0.24	0.1	1.31	1.17
PPUTLS46_018411	beta-lactamase/D-alanine carboxypeptidase	17.44	16.69	17.94	17.14	16.38	16.18	0.03	-0.23	1.01	2.01
PPUTLS46_012150	glycogen branching enzyme	17.43	18.29	20.23	19.36	16	16.91	1.23	0.02	2.38	1.01
PPUTLS46_005051	aldehyde dehydrogenase	17.42	17.51	16.7	17.16	17.58	15.87	-0.09	0.01	0.82	0.99
PPUTLS46_022181	ornithine carbamoyltransferase	17.42	17.58	17.47	18.06	17.29	18.26	0.54	0.29	0.36	0.69
PPUTLS46_014689	Peptidase M23B	17.4	16.25	16.38	16.88	16.08	14.6	0.02	-0.13	0.99	1.15
PPUTLS46_005096	cell division protein FtsX	17.4	15.63	17.96	15.55	15.51	15.83	1.47	-0.01	0.22	1.04
PPUTLS46_007166	preprotein translocase subunit SecG	17.38	18.92	18.43	18.25	18.43	16.9	0.31	0.01	0.76	1.02
PPUTLS46_024158	ubiquinone biosynthesis protein COQ7-like	17.38	16.51	18.17	17.23	18.06	18.15	0.18	1.02	1.25	3.73
PPUTLS46_013703	methyl-accepting chemotaxis sensory transducer	17.38	16.69	18.18	17.68	17.23	16.22	0.65	-0.31	3.64	0.45

PPUTLS46_001277	cytidine/deoxycytidylate deaminase family	17.38	16.85							3.56	3.79
PPUTLS46_025068	LysM domain/BON superfamily protein	17.36	19.57	22.34	21.59	16.99	19.35	2.14	-2.27	2.31	0.03
PPUTLS46_020221	cytochrome c-type biogenesis protein CcmE	17.35	17.88	17.43	17.5	17.03	17.2	-0.29	-0.21	0.49	0.61
PPUTLS46_024193	anthranilate synthase component II	17.34	16.82	17	15.76	15.68	17.41	-0.04	-0.26	1.11	0.77
PPUTLS46_025358	alginate regulatory protein AlgP	17.33	19.07	19.58	18.03	18.57	19.33	0.17	0.45	1.09	0.72
PPUTLS46_020661	succinylglutamic semialdehyde dehydrogenase	17.33	18.15	17.56	16.7			-0.18		1.24	0.71
PPUTLS46_005536	methyl-accepting chemotaxis sensory transducer	17.31	18.59	18.48	18.88	18.88	18.76	0.36	0.25	0.72	1.2
PPUTLS46_014284	spermidine/putrescine ABC transporter ATPase	17.31	17.19	16.87	17.86	17.32	17.45	0.58	0.15	0.48	1.5
PPUTLS46_012195	propionyl-CoA carboxylase	17.31	14.89	17.48	14.13	16.68	16.12	-2.08	0.49	0.21	0.67
PPUTLS46_001557	extracellular solute-binding protein	17.29	16.33			16.37	16.02		-0.01	0.94	0.9
PPUTLS46_003002	S-transferase	17.29	17.08	17.58	17.29	15.67	16.62	0.15	-0.07	2.08	1.13
PPUTLS46_024808	hypothetical protein	17.28	16.87	17.88	18.04	17.18	17.8	0.51	0.24	2.23	1.42
PPUTLS46_004776	RND family efflux transporter MFP subunit	17.27	16.95	18.79	18.11	17.24	17.8	1.05	0.25	4.65	1.47
PPUTLS46_007974	TenA family transcription regulator	17.27	14.96							1	1.29
PPUTLS46_016489	methionyl-tRNA formyltransferase	17.26	17.98	17.9	17.19	17.54	17.57	0.03	0.28	1	0.66
PPUTLS46_019996	xanthine dehydrogenase	17.25	17.26	18.2	17.79			0.52		3	1.17
PPUTLS46_001832	FKBP-type peptidylprolyl isomerase	17.24	16.28			17.36	16.23		0.46	0.94	0.45
PPUTLS46_016374	luciferase family protein	17.24	16.35	16.02	14.97	15.87	14.78	-0.62	-0.41	2	1.61
PPUTLS46_000310	Uncharacterized protein conserved in bacteria	17.22	16.64	18.57	18.25	18.19	17.73	1.32	0.97	11.34	4.69
PPUTLS46_000305	hypothetical protein	17.2	17.9	19.39	19.17	17.7	17.01	1.1	0.04	2.62	0.99
PPUTLS46_007171	ribosome maturation protein RimP	17.2	17.45	17.07	17.41	16.17	17.28	-0.51	-0.49	0.17	0.45
PPUTLS46_015759	isochorismatase superfamily hydrolase	17.19	15.78	18.44	17.09	17.63	18.03	0.23	0.93	1.19	2.17
PPUTLS46_011900	Siroheme synthase	17.18	17.52	17.75	16.46	18.32	18.5	0.02	0.75	0.99	2.42
PPUTLS46_015159	quinone oxidoreductase	17.18	16.56	17.63	17.03	17.65	17.41	0.19	0.71	1.62	4.89
PPUTLS46_023548	ethanolamine ammonia-lyase small subunit	17.18	17.48	17.96	17.15			0.08		1.09	0.76
PPUTLS46_000485	Clostridium P-47 protein	17.17	17.94	21.81	20.92	19.12	19.75	3	1.12	4.6	2.2
PPUTLS46_024898	ABC transporter related protein	17.17	17.85	18.15	16.67	16.05	16.77	0.01	-0.07	0.98	0.89
PPUTLS46_002777	exonuclease III	17.15	16.28	17.49	16.06	16.22	15.81	0.65	-0.02	0.34	1.12
PPUTLS46_014028	pyridoxine 5'-phosphate synthase	17.14	18.72	18.39	18.09	17.08	18.18	0.22	-1.14	0.84	0.23
PPUTLS46_025694	diguanylate cyclase/phosphodiesterase	17.12	16.73	18.16	17.54	17.86	17.58	0.69	0.91	3.85	10.24
PPUTLS46_003667	YD repeat-containing protein	17.1	17.42	19.82	18.92	18.48	18.35	1.54	0.92	3.42	2.97
PPUTLS46_001052	response regulator/TPR domain-containing protein	17.1	16.52	15.69	14.99	16.7	15.02	-1.1	-0.08	4.56	0.89
PPUTLS46_014139	hexapeptide repeat-containing transferase	17.1	16.18	17.34	17.46	16.28	17.32	0.39	0.05	1.74	1.02
PPUTLS46_007016	hypothetical protein	17.1	16.93			15.95	15.87		-0.63	1.76	5.94
PPUTLS46_022776	ABC-type transport system involved in resistance	17.09	17.56	15.59	13.49	18.15	17.43	-1.48	0.3	2.02	1.4
PPUTLS46_025408	glutathione S-transferase domain-containing	17.09	17.34	19.12	18.92	17.84	17.47	1.39	0.36	4.44	1.76
PPUTLS46_017999	2-octaprenyl-3-methyl-6-methoxy-1,4-benzoquinol	17.08	15.24	18.03	18.43	16.01	16.82	1.2	0.09	2.11	1.05
PPUTLS46_009599	TatD-related deoxyribonuclease	17.04	17.51			17.82	17.19		0.16	1.13	1.2
PPUTLS46_007674	hypothetical protein	17.03	18.25	18.62	19.46	17.08	16.87	0.05	-0.12	1.01	0.86
PPUTLS46_018024	glycine dehydrogenase	17.03	15.46	20.47	19.43	17.06	16.12	2.69	0.4	3.9	0.62
PPUTLS46_020761	RNA-binding S4 domain-containing protein	17.03	18.21	18.18	16.96	15.1	14.47	0.01	-1.51	1.02	2.45
PPUTLS46_000035	aminotransferase AlaT	17.02	17.39	17.94	16.3	17.44	17.35	-0.08	0.07	0.9	1.08
PPUTLS46_012883	methyl-accepting chemotaxis sensory transducer	17.02	16.39	17.74	17.53	14.81	15.22	0.74	-0.94	5.04	3.43
PPUTLS46_004224	D-alanyl-D-alanine	17.02	18.31	17.66	17.81			0.53		0.54	0.69
PPUTLS46_005846	response regulator receiver modulated PAS/PAC	17.01	16.55	17.09	17.33	17.06	16.45	0.06	0.07	1.06	0.89
PPUTLS46_000375	diguanylate cyclase/phosphodiesterase with	17.01	16.58	18.34	17.82	15.63	16.85	1.09	-0.19	7.89	0.76
PPUTLS46_022521	two component transcriptional regulator	17	16.41	15.97	15.61			-0.76		7.25	5.59
PPUTLS46_018049	adenylate cyclase	16.99	16.56	17.13	16.85	17.03	17.01	0.16	0.41	3.5	5.17
PPUTLS46_006971	hypothetical protein	16.99	16.75			16.87	17.02		0.13	0.82	1.46
PPUTLS46_006916	acyl-CoA thioesterase II	16.99	14.33	16.6	16.37	14.88	14.13	0.05	-0.4	0.99	0.72
PPUTLS46_008554	outer membrane efflux protein	16.97	17.93	15.76	16.06			-0.57		1.64	0.67
PPUTLS46_019971	NAD-dependent DNA ligase LigA	16.96	15.46	16.57	16.69	16.85	15.59	0.09	0.84	1.08	0.23
PPUTLS46_024468	carboxylesterase	16.95	15.57	18.41	17.37	14.35	16.61	0.76	-0.07	1.87	0.94
PPUTLS46_005611	poly(3-hydroxyalkanoic acid) synthase 2	16.93	17.01	19.85	19.52	19.83	19.34	2.38	2.34	9.29	7.09
PPUTLS46_021276	cystathionine gamma-lyase	16.92	14.08	17.15	16.03	17.48	18.15	0.36	1.18	0.8	1.67
PPUTLS46_002992	amidase	16.91	17.14	15.32	16.19	17.28	16.73	-0.25	0.08	1.27	1.11
PPUTLS46_013338	carboxylesterase	16.89	16.69	16.12	16.08	16.59	18.46	-0.42	0.12	2.75	0.94
PPUTLS46_012425	NolW domain protein	16.86	15.64	18.57	17.5	17.58	15.61	0.96	0.64	2.2	0.54
PPUTLS46_006106	rhodanese domain-containing protein	16.86	17.7	18.72	18.02	16.04	17.49	0.54	-1.01	1.64	0.22
PPUTLS46_019271	extracellular solute-binding protein	16.86	18.31	19.75	19.54			1.08		1.94	0.68
PPUTLS46_014003	GTP-binding protein LepA	16.85	17.49	18.23	18.11	18.24	17.41	0.43	0.39	1.63	1.44
PPUTLS46_005321	choline dehydrogenase	16.85	18.08	17.91	18.53	15.24	16.47	0.48	-0.09	0.64	0.91
PPUTLS46_020481	flagellar basal body P-ring protein	16.84	16.28	18.6	19.07	17.38	12.99	1.61	-0.61	3.48	0.77
PPUTLS46_010269	Uncharacterized protein conserved in bacteria	16.83	18.49	18.57	18.44	19.14	17.91	0.06	0.31	0.98	1.18
PPUTLS46_015434	bifunctional 3-demethylubiquinone-9	16.83	18.31	18.26	16.99	16.2	17.84	0.06	-1.39	1.02	0.16
PPUTLS46_013518	UDP-N-acetylmuramoyl-L-alanyl-D-glutamate	16.82	17.81	18.22	17.95	17.88	18.23	0.18	0.09	1.17	1.06
PPUTLS46_013818	AraC family transcriptional regulator	16.82	16.94							0.68	1.97
PPUTLS46_005971	Lipid A export ATP-binding/permease protein msbA	16.79	17.16	17.63	18.04	16.58	17.16	0.1	0.56	1.1	0.21
PPUTLS46_021446	quinolinate synthetase	16.79	16.62	15.1	14	16	16.44	-1.52	-0.12	3.49	0.64
PPUTLS46_015539	dTDP-4-dehydrorhamnose 3,5-epimerase	16.78	16.97			16.57	17.37		0.39	0.68	0.51
PPUTLS46_016859	DNA-dependent helicase II	16.73	18.17			15.43	16.87		-0.57	0.94	0.62
PPUTLS46_004072	cytochrome o ubiquinol oxidase subunit II	16.72	18.83			18.65	20.02		0.2	0.98	0.91
PPUTLS46_009574	hypothetical protein	16.72	17.46	19.47	18.94	18.27	17.95	1.47	0.62	3.06	1.81
PPUTLS46_002142	Pyrrolo-quinoline quinone	16.7	17.18	19.22	18.83	17.59	17.68	1.56	0.38	3.91	1.6
PPUTLS46_017424	extracellular solute-binding protein	16.69	15.97	16.72	18.26	18.56	17.88	0.05	1.6	1.01	4.71
PPUTLS46_024413	pyrroloquinoline quinone biosynthesis protein	16.69	16.01	17.95	18.1			1.24		3.87	5.56
PPUTLS46_020471	flagellar hook-associated protein FlgK	16.68	18.84	18.69	19.9	19.15	18.68	0.44	0.22	0.79	1.11
PPUTLS46_025148	glutamate--cysteine ligase	16.68	16.78	15.44	15.85	16.9	17.12	-0.41	0.14	1.67	1.27
PPUTLS46_015164	HAD family hydrolase	16.68	17.1	17.61	17.74			0.19		1.26	1.31
PPUTLS46_012565	hydroxyacylglutathione hydrolase	16.66	17.79	18.11	16.79	18.49	16.99	0.07	0.21	1.03	1.13
PPUTLS46_005076	peptidase M16 domain-containing protein	16.66	16.24	17.28	17.37	17.77	16.29	0.54	0.12	2.51	1.1
PPUTLS46_012828	methylmalonate-semialdehyde dehydrogenase	16.64	18.37	22.38	22.06	18.58	17.68	3.45	0.13	3.67	1.07
PPUTLS46_015549	dTDP-4-dehydrorhamnose reductase	16.64	16.11	14.91	15.87	14.88	16.2	-0.17	-0.05	1.19	0.92
PPUTLS46_020586	branched-chain alpha-keto acid dehydrogenase	16.64	15.57	17.53	15.68			0.68		0.49	3.02
PPUTLS46_020726	pterin-4-alpha-carbinolamine dehydratase	16.64	16.68	17.28	16.97			0.25		1.84	3.06

PPUTLS46_004334	molybdenum cofactor biosynthesis protein B	16.62	15.54	17.11	16.64	15.74	17.26	0.36	0.09	1.76	1.05
PPUTLS46_013833	Conserved protein/domain typically associated	16.61	15.62	14.86	14.76			-0.93		3.32	3.38
PPUTLS46_016064	citrate transporter	16.61	16.35							4.29	13.23
PPUTLS46_010919	glyoxalase/bleomycin resistance protein/dioxygen	16.6	14.67	15.63	16.05	13.51	14.58	0.03	-0.44	1	1.36
PPUTLS46_005016	SAM-dependent methyltransferase	16.57	16.81	17.99	18.15	16.62	13.65	0.8	-0.08	2.44	1.06
PPUTLS46_000045	glutathione peroxidase	16.57	17.23	18.35	17.72	14.14	14.21	0.62	-1.62	1.89	3.41
PPUTLS46_012918	ubiquinol oxidase subunit I, cyanide insensitive	16.55	17.09	19.63	19.15	21.57	20.89	2	3.72	4.45	6.34
PPUTLS46_002227	acyl-CoA synthetase	16.55	16.9			17.5	16.7		0.26	0.71	1.36
PPUTLS46_024833	glycine betaine/L-proline ABC transporter,	16.55	17.79	17.33	18.6			0.89		0.46	0.74
PPUTLS46_020486	flagellar basal body L-ring protein	16.54	15.6	17.46	17.23			0.93		3.55	3.78
PPUTLS46_024708	hypothetical protein	16.54	17.73							0.82	0.76
PPUTLS46_020746	FAD/FMN-containing dehydrogenases	16.53	16.34	14.96	17.12	16.3	15.39	-0.76	-0.01	0.6	1.08
PPUTLS46_003527	type VI secretion lipoprotein, VC_A0113 family	16.52	16.58			18.15	19.71		1.4	1.1	2.17
PPUTLS46_007081	SmpA/OmlA domain-containing protein	16.52	16.75	16.64	15.8	16.17	16.47	-0.12	-0.35	1.3	0.23
PPUTLS46_002012	Extracellular solute-binding protein	16.52	16.75							0.81	2.21
PPUTLS46_009829	multi-sensor signal transduction histidine	16.5	17.35	15.63	16.18	15.14	17.2	0.02	-1.01	1.01	0.38
PPUTLS46_002302	hydroxymethylglutaryl-CoA lyase	16.49	17.06	15.12	16.87	17.66	18.07	-0.75	0.58	0.55	1.78
PPUTLS46_025113	RNA-binding S4 domain-containing protein	16.49	16.66							0.93	2.61
PPUTLS46_022276	glutamate synthase	16.48	16.96	18.63	18.09			1.15		3.02	1.49
PPUTLS46_016454	coproporphyrinogen III oxidase	16.44	14.04			16.31	16.01		0.1	1.48	1.05
PPUTLS46_021371	hypothetical protein	16.43	16.83			17.24	15.99		0.05	0.79	1.02
PPUTLS46_013748	3-oxoadipate enol-lactonase	16.41	16.73			16.23	15.98		0.01	0.86	0.96
PPUTLS46_003777	glycine betaine/L-proline ABC transporter	16.4	16.22							3.86	9.42
PPUTLS46_013333	ATP-dependent RNA helicase RhlB	16.38	16.38	15.95	16.31	17.01	16.67	-0.25	0.48	0.52	2.59
PPUTLS46_020026	hydroxyisourate hydrolase	16.38	16.61	16.22	15.79	16.83	16.37	-0.21	0.14	1.63	1.23
PPUTLS46_021356	malate:quinone oxidoreductase	16.38	16.33	16.69	18.34			0.27		0.85	5.29
PPUTLS46_006251	replicative DNA helicase	16.36	16.15	14.25	13.53			-1.99		7.19	11.05
PPUTLS46_009579	CHAD domain-containing protein	16.31	16.82	19.37	19.65	17.04	16.86	2.16	0.2	3.94	1.29
PPUTLS46_004384	ABC transporter ATP-binding protein	16.31	15.63			16.52	17.35		0.63	4.48	1.89
PPUTLS46_021916	branched chain amino acid ABC transporter	16.31	18.22	19.11	19.02	16.47	16.89	0.64	-0.62	1.43	0.57
PPUTLS46_025679	hypothetical protein	16.31	13.77			12.4	13.03		-0.79	1.54	1.55
PPUTLS46_012660	hypothetical protein	16.28	16.06	17.15	16.64	16.25	17.2	0.56	0.17	4.03	1.19
PPUTLS46_006831	DEAD/DEAH box helicase domain-containing protein	16.26	17.42	15.81	16.22	16.08	15.58	-0.16	-0.09	0.83	1.11
PPUTLS46_019311	ABC transporter related protein	16.23	16.93	14.7	15.12			-0.74		1.88	1.41
PPUTLS46_014839	cysteine synthase B	16.22	16.54			16.9	17.08		0.35	1.19	1.64
PPUTLS46_019946	DNA-binding protein, YbaB/EbfC family	16.21	16.96			18.88	17.74		1.12	0.8	2.09
PPUTLS46_016814	cobalamin synthesis protein, P47K	16.2	15.93							10.89	20.28
PPUTLS46_026301	hypothetical protein	16.19	19.19	21.24	21.17	18.29	17.54	1.71	0.35	1.81	0.86
PPUTLS46_006956	hypothetical protein	16.19	15.42							4.2	6.2
PPUTLS46_025896	GntR family transcriptional regulator	16.18	16.98	18.64	17.38			0.74		1.69	1.33
PPUTLS46_019976	cell division protein ZipA	16.17	19.18	19.08	19.25	17.31	17.35	0.09	-0.66	0.97	0.69
PPUTLS46_001722	amidase	16.15	14.51							2.04	2.6
PPUTLS46_014244	L-carnitine dehydratase/bile acid-inducible	16.13	16.75	16.21	16.39	17.25	16.52	-0.4	0.26	0.41	1.3
PPUTLS46_002197	glycine betaine ABC transporter	16.11	17.54	19.97	18.08	17.03	18.31	1.08	0.5	1.64	0.69
PPUTLS46_025816	nitrate/sulfonate/bicarbonate ABC transporter	16.1	16.96	16.8	17.02	18.17	18.35	0.42	1.09	0.56	2.28
PPUTLS46_007231	acetyl-CoA synthetase	16.08	16.92	18.26	17.46	17.26	16.15	0.76	0.12	1.87	1.09
PPUTLS46_022206	DeoR family transcriptional regulator	16.06	15.77	16.27	16.41	14.55	15.14	0.07	-0.29	1.1	1.65
PPUTLS46_021946	pyridoxamine 5'-phosphate oxidase	16.05	17.91	16.12	17.1	18.29	19.99	-1.07	0.38	0.35	1.18
PPUTLS46_000937	PTS system fructose-specific transporter	16.01	15.1	14.96	14.69			-0.43		2.43	5.35
PPUTLS46_001657	FAD-dependent pyridine nucleotide-disulfide	16	16.27	18.81	16.87	16.95	16.64	0.69	0.52	1.49	2.15
PPUTLS46_025619	surface adhesion protein	15.94	16.93	15.04	15.05	17.55	18.23	-0.55	0.59	1.67	1.54
PPUTLS46_023708	riboflavin biosynthesis protein RibD	15.92	17.2	16.63	16.48	16.02	16.74	-0.25	0.86	0.72	0.32
PPUTLS46_020301	chemotaxis phosphatase, CheZ	15.88	17.31	17.92	17.54	17.04	17.15	0.35	0.21	1.3	0.85
PPUTLS46_009584	Acyl-CoA thioesterase-like protein	15.86	15.78			19.23	19.1		3.19	5.24	17.75
PPUTLS46_020861	ATP-dependent DNA helicase RecQ	15.85	15.48							168.42	30
PPUTLS46_004594	AraC family transcriptional regulator	15.85	16.4							1.38	2.26
PPUTLS46_014074	ATP-dependent protease La	15.82	17.48	16.98	16.94			0.38		0.72	0.91
PPUTLS46_024888	hypothetical protein	15.8	15.94			15.71	15.56		0.11	2.95	0.77
PPUTLS46_003937	Predicted outer membrane lipoprotein	15.78	15.91	16.78	16.37	15.26	15.72	0.46	-0.39	2.36	0.2
PPUTLS46_020156	hydantoin racemase	15.76	16.13	15.77	16.47			0.77		0.2	3.1
PPUTLS46_018531	Putative MetA-pathway of phenol degradation	15.71	12.81	16.99	18.54	14.77	16.66	1.85	0.54	1.85	1.24
PPUTLS46_024478	biotin synthase	15.67	16.87							0.94	1.32
PPUTLS46_017989	2-octaprenyl-6-methoxyphenyl hydroxylase	15.64	14.27	16.75	16.56	15.17	14.64	1.11	0.41	2.77	0.47
PPUTLS46_021616	virulence factor family protein	15.6	17.73	17.75	17.74	17.21	16.61	0.07	0.23	0.97	0.88
PPUTLS46_002467	cobalamin biosynthesis protein CobW	15.58	17.74	15.75	17.16	16.74	17.37	-1.58	0.74	0.22	0.59
PPUTLS46_004454	Protein of unknown function (DUF2803)	15.56	17.19	15.23	15.96	13.88	15.16	-0.54	-0.06	0.62	0.94
PPUTLS46_013398	alpha/beta fold family hydrolase-like protein	15.54	13.5	15.57	15.9			0.48		1.39	2.69
PPUTLS46_016189	GTP-binding protein YsxC	15.51	17.88	18.86	16.43	17.55	17.24	0.23	0.2	1.08	0.9
PPUTLS46_003512	ImcF domain-containing protein	15.5	16.84	18.92	18.09	18.04	18.24	1.44	1.05	2.26	1.89
PPUTLS46_022891	cytoplasmic axial filament protein	15.5	17.83	17.47	17.66	15.51	17.17	0.34	-1.66	0.82	0.21
PPUTLS46_004781	acriflavin resistance protein	15.49	16.1	16.51	17.46	16.62	16.27	0.07	0.39	0.96	1.54
PPUTLS46_019991	xanthine dehydrogenase, small subunit	15.47	15.52	18.33	16.71			1.11		2.03	6.89
PPUTLS46_014704	DNA mismatch repair protein MutS	15.47	17.37	15.17	17			-1.8		0.16	0.97
PPUTLS46_014379	hypothetical protein	15.46	15.01	17.38	16.83	15.82	15.57	1.65	0.6	9.88	7.27
PPUTLS46_009444	methyl-accepting chemotaxis transducer	15.45	15.36	17.16	17.91	17.43	16.86	1.23	1.57	2.38	5.51
PPUTLS46_006566	Zinc finger-domain-containing protein	15.44	17.47	17.07	14.54			-0.03		1.03	0.93
PPUTLS46_024688	serine hydroxymethyltransferase	15.43	15.2			14.31	15.43		-0.29	15.72	0.62
PPUTLS46_013978	L-aspartate oxidase	15.39	14.85	15.66	16.75	16.08	17.17	0.17	0.97	1.12	2.13
PPUTLS46_024238	spermidine/putrescine ABC transporter ATPase	15.39	17.38	20.2	19.62	15.07	16.38	2.24	-1.21	2.52	0.32
PPUTLS46_023633	exodeoxyribonuclease VII small subunit	15.34	16.37	14.33	13.64	16.1	15.28	-1.09	0.06	2.24	0.97
PPUTLS46_022711	nucleoid-associated protein NdpA	15.23	13.96	16.57	15.2	16.31	14.53	0.28	0.21	1.25	0.86
PPUTLS46_017934	flagellar basal body-associated protein	15.12	14.65	13.32	15.47			-0.59		0.68	36.64
PPUTLS46_010529	ribokinase	15.06	15.35							3.88	5.05
PPUTLS46_008284	bifunctional aldehyde dehydrogenase/enoyl-CoA	15.02	17.22			14.33	16.05		-1.34	0.88	0.34
PPUTLS46_002047	RND family efflux transporter MFP subunit	14.98	13.83	14.52	14.6	15.36	13.25	0.04	0.85	1.01	0.42

PPUTLS46_007789	glutamate--putrescine ligase	14.96	14.75	14.53	14.63	14.81	16.57	-0.01	0.06	0.89	1.02
PPUTLS46_014184	16S rRNA-processing protein RimM	14.95	15.94			15.35	16.73		0.58	1.76	0.59
PPUTLS46_020281	flagellar motor protein MotD	14.93	15.2			15.98	15.52		0.54	4.35	2.1
PPUTLS46_023101	ColR	14.87	16.28	15.49	16.09	13.56	15.7	0.88	-1.21	0.36	0.35
PPUTLS46_025846	FAD-dependent pyridine nucleotide-disulfide	14.83	14.07							9.98	11.29
PPUTLS46_023181	Extracellular solute-binding protein	14.8	15.81	19.56	17.94	19.32	16.25	2.3	1.1	2.72	1.5
PPUTLS46_018099	PotI	14.75	17.03	14.79	14.78	17.36	16.75	-0.02	0.23	0.97	1.11
PPUTLS46_017969	cell division protein ZapA	14.75	14.86	15.9	15.79	15.87	17.44	0.68	0.89	2.88	1.73
PPUTLS46_021166	VWA containing CoxE family protein	14.73	16.02			17.41	17.94		1.28	1.57	2.06
PPUTLS46_001432	CreA family protein	14.73	14.34							335.64	207.53
PPUTLS46_013793	carboxyphosphonoenolpyruvate phosphonomutase	14.66	17.93	16.79	15.6			-0.07		0.95	0.84
PPUTLS46_016019	NAD(P)(+) transhydrogenase	14.57	14.7	15.77	14.98	18.06	19.1	0.42	3.1	1.8	4.59
PPUTLS46_014134	NUDIX hydrolase	14.4	15.92	11.89	13.31	13.54	11.48	-0.69	-1.08	1.4	1.63
PPUTLS46_025348	ABC transporter-like protein	14.23	15.63			15.89	15.81		0.22	1.86	1.17
PPUTLS46_012993	acetyl-CoA acetyltransferase	14.18	15.54	15.19	15.66	23.47	22.62	0.55	6.72	0.58	6.46
PPUTLS46_020601	hypothetical protein	14.14	14.07							13.8	16.35
PPUTLS46_010259	alkyl hydroperoxide reductase subunit F	13.94	15.49			16.86	14.89		0.47	1.9	1.25
PPUTLS46_019951	DNA polymerase III subunits gamma and tau	13.85	16.39							1.22	1.35
PPUTLS46_017054	histone family protein DNA-binding protein	13.75	13.12	17.38	13.94	13.09	16.1	0.15	0.07	1.05	0.98
PPUTLS46_021926	FAD dependent oxidoreductase	13.57	15.7	16.27	15.64			0.38		1.22	1.77
PPUTLS46_014714	16.7 kDa protein in recA 5'region	13.54	14.76	14.74	14.68	12.6	14.46	0.18	-1.29	0.85	0.22
PPUTLS46_005476	histidine ammonia-lyase	13.11	15.92							1.4	1.51
PPUTLS46_014609	DNA polymerase III subunit alpha	13.02	17.05							1.02	1.06
PPUTLS46_019231	PAS/PAC sensor hybrid histidine kinase	11.9	11.37							54.68	50.98
PPUTLS46_005596	poly-(hydroxyalkanoate) granule-associated			23.14	22.56	23.71	23.31			31.06	35.2
PPUTLS46_007649	3-oxoacid CoA-transferase, B subunit					22.27	22.53				19.09
PPUTLS46_000635	Dihydrodipicolinate synthetase					19.99	21.18				3.96
PPUTLS46_006576	dehydratase			20.24	19.13	19.45	18.31			4.96	2.83
PPUTLS46_018416	hypothetical protein					18.95	19.7				3.94
PPUTLS46_011580	phosphoglycerate mutase					18.82	19.07				7.35
PPUTLS46_025911	amino acid transporter LysE					18.59	17.76				2.65
PPUTLS46_009959	hypothetical protein					18.51	16.7				0.86
PPUTLS46_003412	acetoacetyl-CoA synthetase			17.87	16.83	18.08	17.2			1.47	1.51
PPUTLS46_009944	protein YhgI					17.97	17.47				3.2
PPUTLS46_015219	cell division topological specificity factor			18.34	17.79	17.89	17.07			8.25	1.32
PPUTLS46_003502	OmpA domain-containing protein					17.81	15.41				0.67
PPUTLS46_024458	dithiobiotin synthetase			18.67	16.59	17.76	17.1			0.96	1.52
PPUTLS46_007714	GMC oxidoreductase					17.65	16.03				0.63
PPUTLS46_007724	glycosyl transferase			19.58	18.68	17.51	17.95			5.55	2.24
PPUTLS46_018171	alpha/beta hydrolase fold family protein					17.35	17.51				2.73
PPUTLS46_003357	hypothetical protein					17.27	16.93				1.46
PPUTLS46_017694	glucose dehydrogenase					17.25	16.18				0.65
PPUTLS46_021841	secretion protein HlyD family protein			15.73	14.51	17.15	14.41			2.08	0.89
PPUTLS46_000640	class II aldolase/adducin domain protein					16.95	17.96				1.06
PPUTLS46_017954	Uncharacterized conserved protein			17.63	18.55	16.93	16.29			1.61	0.91
PPUTLS46_025198	taurine ABC transporter periplasmic binding			18.86	18.47	16.86	15.5			44.42	1.02
PPUTLS46_014664	S-formylglutathione hydrolase			17.78	18.89	16.69	17.48			1.64	0.88
PPUTLS46_000470	Uncharacterized protein conserved in bacteria					16.6	14.81				1.2
PPUTLS46_025213	taurine dioxygenase					16.47	15.23				1.45
PPUTLS46_004154	cmaX protein			16.36	15.94	16.34	15.55			5.61	2.12
PPUTLS46_016864	glucose-6-phosphate 1-dehydrogenase					16.33	14.64				1.43
PPUTLS46_021236	hypothetical protein			17.25	15.45	16.27	15.13			0.61	1.77
PPUTLS46_012818	OmpA/MotB domain-containing protein			15.09	15.7	16.27	16.96			1.68	0.95
PPUTLS46_010304	gamma-glutamylputrescine oxidoreductase			16.22	14.89	16.24	15.41			1.37	2.24
PPUTLS46_015504	Glycosyltransferase					16.17	15.6				3.28
PPUTLS46_000560	3-carboxymuconate cyclase			18.57	18.22	16.02	15.17			135.81	2.62
PPUTLS46_007659	XRE family transcriptional regulator			16.05	15.07	15.38	13.61			1.98	2.16
PPUTLS46_020186	D-isomer specific 2-hydroxyacid dehydrogenase,					15.38	14.99				9.41
PPUTLS46_015229	septum formation inhibitor			15.05	14.05	15.25	18.04			3.72	0.74
PPUTLS46_000100	hypothetical protein					15.17	14.8				11.44
PPUTLS46_012725	pirin domain-containing protein					15.16	16.99				0.94
PPUTLS46_024178	Lipoate-protein ligase A					15.03	15.85				2.24
PPUTLS46_000105	hypothetical protein			17.52	17.02	15	15.02			5.24	16.23
PPUTLS46_005946	Glycosyltransferase			16.83	15.52	14.81	15.93			0.76	1.84
PPUTLS46_024123	anhydro-N-acetylmuramic acid kinase					14.73	16.03				1.64
PPUTLS46_003642	hypothetical protein					14.73	16.45				1.22
PPUTLS46_006591	peptidase S16, lon domain-containing protein					14.37	14.51				12.15
PPUTLS46_006431	branched-chain amino acid ABC transporter			22.35	21.89					51.5	
PPUTLS46_019196	Uncharacterized conserved protein			21.09	20.81					108.31	
PPUTLS46_017814	ammonium transporter			20.68	20.6					20.1	
PPUTLS46_001002	methyl-accepting chemotaxis sensory transducer			19.94	19.86					16.47	
PPUTLS46_024503	amino acid-binding ACT domain-containing protein			19.67	17.93					2.05	
PPUTLS46_001317	polyphosphate kinase 2, PA0141 family			19.66	18.84					6.76	
PPUTLS46_016979	phosphate binding protein			19.64	18.45					3.59	
PPUTLS46_012120	4-alpha-glucanotransferase			19.53	18.66					5.82	
PPUTLS46_013438	cytochrome b/b6 domain-containing protein			19.5	18.26					3.19	
PPUTLS46_004199	nitrate-binding protein NasS			19.4	18.97					32.37	
PPUTLS46_009729	2-methylcitrate dehydratase			19.33	18.35					4.36	
PPUTLS46_013358	amino acid ABC transporter ATP-binding protein			19.17	18.79					59.68	
PPUTLS46_019201	Uncharacterized protein conserved in bacteria			19.07	19.61					3.96	
PPUTLS46_006426	Inner-membrane translocator			18.94	18.43					14.29	
PPUTLS46_001577	malate dehydrogenase			18.91	18.27					8.12	
PPUTLS46_001322	isochorismatase hydrolase			18.8	16.76					1.07	
PPUTLS46_023598	hemerythrin HHE cation binding domain-containing			18.77	18.86					6.57	
PPUTLS46_009524	extracellular solute-binding protein, family 1			18.77	17.23					1.58	

PPUTLS46_023378	Domain of unknown function (DUF4136)	18.59	19.63	2.36
PPUTLS46_013803	NAD-dependent epimerase/dehydratase	18.34	17.93	23.24
PPUTLS46_005651	bifunctional pyrimidine regulatory protein PyrR	18.34	17.87	13.41
PPUTLS46_026466	extracellular solute-binding protein	18.14	17.34	3.11
PPUTLS46_002712	quinohemoprotein amine dehydrogenase, beta	18.05	15.45	0.61
PPUTLS46_009589	Phage terminase, small subunit	18.05	16.74	1.2
PPUTLS46_020386	anti-sigma-factor antagonist	17.96	17.59	35.92
PPUTLS46_006411	ABC transporter related protein	17.87	15.89	0.64
PPUTLS46_010924	major facilitator superfamily transporter	17.87	16.79	1.39
PPUTLS46_007186	ribosome-binding factor A	17.71	17.39	145.98
PPUTLS46_005931	glycosyl transferase, group 1	17.7	16.24	0.74
PPUTLS46_016914	ABC transporter, periplasmic polyamine-binding	17.67	18.54	1.69
PPUTLS46_010934	glyoxalase/bleomycin resistance protein/dioxygen	17.66	16.3	0.77
PPUTLS46_013348	amino acid ABC transporter permease	17.52	17.53	3.54
PPUTLS46_023201	alkaline phosphatase	17.52	15.88	0.6
PPUTLS46_014099	TonB-dependent receptor	17.49	17.08	10.67
PPUTLS46_002172	SCP-like extracellular protein	17.48	17.55	3
PPUTLS46_005951	hypothetical protein	17.48	15.15	0.61
PPUTLS46_005401	signal transduction histidine kinase, nitrogen	17.39	15.43	0.6
PPUTLS46_022286	membrane carboxypeptidase	17.34	15.96	0.59
PPUTLS46_022576	arginine/ornithine antiporter	17.33	17.59	1.98
PPUTLS46_006416	ABC transporter-like protein	17.31	17.52	2.04
PPUTLS46_018361	glyoxalase/bleomycin resistance protein/dioxygen	17.23	18.01	1.32
PPUTLS46_007321	Regulator of competence-specific genes	17.16	17.82	1.32
PPUTLS46_020801	P pilus assembly/Cpx signaling pathway,	17.1	18.14	1.14
PPUTLS46_023538	ethanolamine transporter	17.07	16.42	0.86
PPUTLS46_012903	tRNA (uracil-5-)-methyltransferase	17.07	15.66	0.62
PPUTLS46_018571	urease subunit alpha	17.04	15.74	0.61
PPUTLS46_012065	phenylhydantoinase	17.02	16.41	0.81
PPUTLS46_020061	Nucleoside-binding outer membrane protein	17.02	15.31	0.68
PPUTLS46_004539	type IV pilus assembly PilZ	17.01	16.96	1.99
PPUTLS46_025123	ribosomal protein S6 modification protein	16.98	15.95	0.58
PPUTLS46_012045	Lipocalin family protein	16.98	15.82	0.62
PPUTLS46_003317	Protein of unknown function (DUF3455)	16.96	15.78	0.64
PPUTLS46_013888	alpha/beta hydrolase fold protein	16.95	17.15	1.31
PPUTLS46_005146	Domain of unknown function (DUF4426)	16.93	15.9	0.62
PPUTLS46_021066	activator of Hsp90 ATPase 1 family protein	16.86	16.38	0.62
PPUTLS46_020401	flagellar assembly protein H	16.77	17.17	0.94
PPUTLS46_004194	response regulator receiver and ANTAR domain	16.77	16.66	1.12
PPUTLS46_023743	ABC transporter related protein	16.76	16.58	1.23
PPUTLS46_020366	flagellar basal body-associated protein FliL	16.68	16.47	0.98
PPUTLS46_012095	glucose-6-phosphate 1-dehydrogenase	16.67	16.47	0.91
PPUTLS46_000275	aminodeoxychorismate lyase	16.64	15.5	0.93
PPUTLS46_016564	Beta-lactamase domain protein	16.55	16.78	0.72
PPUTLS46_007301	periplasmic binding protein	16.52	15.55	1.16
PPUTLS46_016919	histone deacetylase superfamily protein	16.48	15.18	1.09
PPUTLS46_007699	Lipid A core - O-antigen ligase and related	16.47	15.29	1.14
PPUTLS46_023076	multidrug efflux protein	16.43	15.48	1.31
PPUTLS46_017824	HAD family hydrolase	16.43	12.59	0.93
PPUTLS46_007829	UspA domain-containing protein	16.32	15.7	2.39
PPUTLS46_002152	LysR family transcriptional regulator	16.3	17.07	0.66
PPUTLS46_015724	methyl-accepting chemotaxis sensory transducer	16.3	16.74	0.67
PPUTLS46_014249	YaeQ family protein	16.22	15.4	1.95
PPUTLS46_026186	glutathione S-transferase	16.21	14.26	1.16
PPUTLS46_023503	MORN repeat-containing protein	16.2	15.96	6.61
PPUTLS46_020286	flagellar motor protein	16.16	14.11	1.18
PPUTLS46_002272	PAS/PAC sensor hybrid histidine kinase	16.09	15.52	3.93
PPUTLS46_019261	beta-lactamase domain-containing protein	16.08	16.68	0.7
PPUTLS46_021996	Uncharacterized protein conserved in bacteria	16.07	15.74	30
PPUTLS46_008949	Predicted ATP-dependent endonuclease of the OLD	15.81	15.9	2.19
PPUTLS46_004499	LolC/E family lipoprotein releasing system,	15.77	16.24	1.1
PPUTLS46_017754	putative restriction endonuclease R.XmaI	15.74	15.44	36.25
PPUTLS46_019681	amino acid ABC transporter periplasmic protein	15.68	14.51	2.23
PPUTLS46_012848	Uncharacterized protein conserved in bacteria	15.67	15.98	1.59
PPUTLS46_023358	dehydratase	15.66	15.43	12.58
PPUTLS46_013593	multi-sensor signal transduction histidine	15.66	14.94	3.99
PPUTLS46_002372	helix-turn-helix domain-containing protein	15.6	13.62	1.56
PPUTLS46_017334	CDF family cobalt/cadmium/zinc transporter	15.57	16.65	0.76
PPUTLS46_005836	lipoprotein	15.31	15.87	1.52
PPUTLS46_025906	GAF sensor hybrid histidine kinase	15.24	16.08	1.16
PPUTLS46_021196	Protein of unknown function (DUF2937)	15.18	14.97	15.83
PPUTLS46_022401	membrane-bound lytic transglycosylase F	15.1	14.3	4.87
PPUTLS46_005486	imidazolonepropionase	15.09	14.24	4.48
PPUTLS46_009239	diguanylate cyclase	14.89	14.97	5.03
PPUTLS46_011840	polysaccharide deacetylase	14.81	15.21	2.72
PPUTLS46_026451	alpha/beta hydrolase	14.69	15.35	2.05
PPUTLS46_004996	haloacid dehalogenase-like hydrolase	14.69	14.48	21.03
PPUTLS46_000050	histidine kinase	14.61	14.73	5.25
PPUTLS46_004494	lipoprotein transporter ATP-binding subunit	14.56	15.34	1.91
PPUTLS46_004244	deoxyguanosinetriphosphate	14.36	13.68	8.99
PPUTLS46_024563	lipopolysaccharide kinase	14.3	14.18	13.82
PPUTLS46_024248	PAS/PAC sensor signal transduction histidine	14.12	12.91	4.29
PPUTLS46_021181	LamB/YcsF family protein	14	14.87	2.28
PPUTLS46_016649	F0F1 ATP synthase subunit A	13.93	12.66	4.31
PPUTLS46_006786	cation diffusion facilitator family transporter	13.66	12	3.5

PPUTLS46_020581	2-oxoisovalerate dehydrogenase subunit beta	13.64	16.22	0.94
PPUTLS46_016724	Protein of unknown function (DUF1329)	13.64	14.85	1.97
PPUTLS46_025333	SPFH domain, Band 7 family protein	13.45	13.1	244.15
PPUTLS46_004986	dihydrofolate reductase	13.44	16.08	0.98
PPUTLS46_015039	GntR family transcriptional regulator	13.35	14.3	2.72
PPUTLS46_023458	3-dehydroquinate dehydratase	13.11	16.75	0.81
PPUTLS46_018256	sensor histidine kinase	13.1	15.61	1.16
PPUTLS46_022111	endonuclease III	13.1	12.51	17.96
PPUTLS46_007459	hypothetical protein	12.46	13.25	4.14
PPUTLS46_012545	lysine decarboxylase	12	12.01	16.5
