# Supplementary material for: Quantitative ‘Omics Analyses of Medium Chain Length Polyhydroxyalkanaote Metabolism in Pseudomonas putida LS46 Cultured with Waste Glycerol and Waste Fatty Acids
Source: PLoS One. 2015 Nov 6;10(11):e0142322. doi: 10.1371/journal.pone.0142322 (PMC4636370; doi:10.1371/journal.pone.0142322)
Supplement: S2 Table — (PDF) [file pone.0142322.s004.pdf]

S2 Table. Numbers of significantly up- and down-regulated genes and gene products in specific COG groups under the specific growth conditions [a].

		Waste glycerol Sta vs Exp				WFA vs WG Exp			
		RNA		Protein		RNA		Protein	
**COG Group**	**Description**	Up	Down	Up	Down	Up	Down	Up	Down
K	Transcription	9	6	1	5	5	9	0	2
C	Energy production and conversion	12	36	6	10	14	19	8	8
O	Posttranslational modification, protein turnover, chaperones	2	6	0	3	4	2	0	0
H	Coenzyme transport and metabolism	5	4	0	2	3	5	0	0
J	Translation, ribosomal structure and biogenesis	3	0	0	2	6	0	1	4
R	General function prediction only	14	9	4	5	18	20	5	4
G	Carbohydrate transport and metabolism	3	10	5	3	4	17	1	7
M	Cell wall/membrane/envelope biogenesis	4	14	3	1	8	6	2	3
T	Signal transduction mechanisms	11	9	6	2	7	9	1	3
P	Inorganic ion transport and metabolism	14	33	6	2	10	13	0	1
S	Function unknown	17	9	8	2	12	11	4	3
E	Amino acid transport and metabolism	29	23	10	2	16	17	4	6
I	Lipid transport and metabolism	2	3	0	0	19	6	12	1
Q	Secondary metabolites biosynthesis, transport & catabolism	4	3	2	2	7	11	2	1
N	Cell motility	11	6	2	2	3	7	1	1
Total		144	175	50	45	141	159	31	30

[a]: WG, Waste glycerol cultures; WFA, Waste fatty acid cultures; Exp, Exponential phase; Sta, Stationary phase
