# Supplementary material for: Quantitative ‘Omics Analyses of Medium Chain Length Polyhydroxyalkanaote Metabolism in Pseudomonas putida LS46 Cultured with Waste Glycerol and Waste Fatty Acids
Source: PLoS One. 2015 Nov 6;10(11):e0142322. doi: 10.1371/journal.pone.0142322 (PMC4636370; doi:10.1371/journal.pone.0142322)
Supplement: S3 Table — (PDF) [file pone.0142322.s005.pdf]

S3 Table. Monomer composition of mcl-PHA synthesized by *P. putida* LS46 grown on biodiesel- derived waste glycerol (WG) and waste fatty acid (WFA) cultures.

	**Monomer composition (mol%)[a]**							
**Conditions**	C6	C8	C9	C10	C12	C12:1	C14	C14:1
waste glycerol	2.69 ± 0.07	24.4 ± 0.53	nd	65.5 ± 0.41	2.42 ± 0.37	4.55 ± 0.32	0.39 ± 0.02	nd
waste fatty acids	4.38 ± 0.11	55.2 ± 0.61	1.02 ± 0.12	32.5 ± 0.30	5.1 ± 0.25	0.99 ± 0.01	0.71 ± 0.14	1.91 ± 0.11

[a]: Unsaturated monomers were determined via GC/MS with 2 mass unit smaller than their saturated 3-hydroxyalkanoic acid standards
