# Supplementary material for: Quantitative ‘Omics Analyses of Medium Chain Length Polyhydroxyalkanaote Metabolism in Pseudomonas putida LS46 Cultured with Waste Glycerol and Waste Fatty Acids
Source: PLoS One. 2015 Nov 6;10(11):e0142322. doi: 10.1371/journal.pone.0142322 (PMC4636370; doi:10.1371/journal.pone.0142322)
Supplement: S4 Table — (PDF) [file pone.0142322.s006.pdf]

S4 Table. Summary of RNA (Rnet) and protein (Pnet) scores from the RNAseq and 1D proteomic analyses under three experimental conditions [a].

Protein scores							RNA scores
Growth Condition[a]	MS/MS collected	Total peptides	Non-redundant peptides	Proteins (loge)<-1	Proteins (loge)<-3	Proteins (loge)<-10	Number of genes identified
WG_Exp_Rep1	33864	19928	8315	1947	1830	1377	5193
WG_Exp_Rep2	34996	17941	7431	1836	1714	1285	5189
WG_Sta_Rep1	36092	21644	9787	1997	1906	1500	5204
WG_Sta_Rep2	35168	20533	9503	1975	1873	1456	5198
WFA_Exp_Rep1	36081	20922	8817	1829	1718	1333	5199
WFA_Exp_Rep1	36587	20982	8844	1825	1713	1330	5195
			**Aerage (Proteins)**				**Aerage (RNA)**
			WG_Exp	1891	1772	1331	5191
			WG_Sta	1916	1810	1478	5201
			WFA_Exp	1827	1715	1331	5197

[a:] WG: Waste glycerol; WFA: waste fatty acids; Exp: exponential phase; Sta: stationary phase; Rep1/2: biological replicate 1 and 2.
