# Supplementary material for: Quantitative ‘Omics Analyses of Medium Chain Length Polyhydroxyalkanaote Metabolism in Pseudomonas putida LS46 Cultured with Waste Glycerol and Waste Fatty Acids
Source: PLoS One. 2015 Nov 6;10(11):e0142322. doi: 10.1371/journal.pone.0142322 (PMC4636370; doi:10.1371/journal.pone.0142322)
Supplement: S5 Table — (PDF) [file pone.0142322.s007.pdf]

S5 Table. Overall biological signal to systematic noise ratio of RNAseq and Proteomic analyses under three experimental conditions [a].

	Comparisions[a]	Standard Deviation	
		RNA	Protein
Biological signal	WG_Sta_Rep1 vs WG_Exp_Rep1	1.22	1.53
	WG_Sta_Rep2 vs WG_Exp_Rep2	1.1	1.52
	WFA_Exp_Rep1 vs WG_Exp_Rep1	1.01	1.59
	WFA_Exp_Rep2 vs WG_Exp_Rep2	1.01	1.53
System noise	WG_Exp_Rep1 vs WG_Exp_Rep2	0.27	1.1
	WG_Sta_Rep1 vs WG_Sta_Rep2	0.51	0.81
	WFA_Exp_Rep1 vs WFA_Exp_Rep2	0.28	1.03
Average ratio		3.48	1.55

a: WG: Waste glycerol; WFA: waste fatty acids; Exp: exponential phase; Sta: stationary phase; Rep1/2: biological replicate 1 and 2.
