# Supplementary material for: Quantitative ‘Omics Analyses of Medium Chain Length Polyhydroxyalkanaote Metabolism in Pseudomonas putida LS46 Cultured with Waste Glycerol and Waste Fatty Acids
Source: PLoS One. 2015 Nov 6;10(11):e0142322. doi: 10.1371/journal.pone.0142322 (PMC4636370; doi:10.1371/journal.pone.0142322)
Supplement: S6 Table — (PDF) [file pone.0142322.s008.pdf]

S6 Table. Variation in expression values of putative fatty acid de novo synthesis genes and gene products in of *P. putida* LS46 under two growth conditions.

						Waste glycerol Sta vs Exp		Waste fatty acid vs Waste glycerol (Exp)	
**Locus Tag**	**Gene Symbol**	**Gene Annotation**	**DNA Seq Length (nt)**	**R[a]**	**P[a]**	**Rnet**	**Pnet**	**Rnet**	**Pnet**
PPUTLS46_000255	fabG 1	3-ketoacyl-(acyl-carrier-protein) reductase	741	12.6	20.1	0.23	0.01	-0.5	-0.06
PPUTLS46_002112	fadG	short-chain dehydrogenase/reductase SDR	741	6.25	nr	0.28	nc	0.06	nc
PPUTLS46_011825	fadG	short-chain dehydrogenase/reductase SDR	765	8.28	nr	0.25	nc	-0.19	nc
PPUTLS46_011830	fadG	3-oxoacyl-ACP reductase	762	7.16	nr	0.68	nc	0.02	nc
PPUTLS46_018726	fadG	short-chain dehydrogenase/reductase SDR	765	6.3	nr	-0.07	nc	-0.66	nc
PPUTLS46_018731	fadG	3-oxoacyl-ACP reductase	738	6.46	nr	-0.51	nc	-1.6	nc
PPUTLS46_023353	fabG 2	3-ketoacyl-(acyl-carrier-protein) reductase	1353	11.3	17.7	-1.39	1.82	-2.24	-0.3
PPUTLS46_015924	fabG 3	3-ketoacyl-(acyl-carrier-protein) reductase	750	10.1	19.5	-0.59	-0.43	-0.47	0.45
PPUTLS46_000265	fabF	3-oxoacyl-(acyl carrier protein) synthase II	1245	12.1	18.3	1.05	-0.14	0.83	0.23
PPUTLS46_012710	fabA	3-hydroxydecanoyl-(acyl carrier protein) dehydratase	516	11.7	17.4	1.13	1.42	1.44	0.07
PPUTLS46_012715	fabB	3-oxoacyl-(acyl carrier protein) synthase I	1221	13.5	20.3	0.46	-0.49	0.79	0.03
PPUTLS46_012998	fabV	trans-2-enoyl-CoA reductase	1212	12.1	21.2	0.44	-0.19	1.8	0.04
PPUTLS46_014589	fabZ	(3R)-hydroxymyristoyl-ACP dehydratase	441	11.1	19.6	-0.39	-0.8	nc	nc
PPUTLS46_020461	fabH	3-oxoacyl-ACP synthase	930	nr	nr	nc	nc	nc	nc
PPUTLS46_000250	fabD	malonyl CoA-acyl carrier protein transacylase	885	12	18.6	-0.02	1.37	-0.05	-0.87
PPUTLS46_023463		acetyl-CoA carboxylase biotin carboxyl carrier protein subunit	462	11.5	21.7	-0.3	-0.92	0.85	-1.22
PPUTLS46_023468		acetyl-CoA carboxylase biotin carboxylase subunit	1356	13.9	22.3	-0.64	0.09	0.54	-0.02
PPUTLS46_000435		acetyl-CoA carboxylase subunit beta	894	nr	nr	nc	nc	nc	nc
PPUTLS46_014614		acetyl-CoA carboxylase carboxyltransferase subunit alpha	948	12.3	20.7	0.75	0	1.12	-0.24

[a]: The log2 value of RNA and Protein expression abundance of *P.putida* LS46 grown in waste glycerol culture at 8hrs post-inocula. RNA or Protein that were either not detected or only observed in one biological replicate were not reported for comparative analysis, and therefore no Rnet and Pnet value calculated. nr: not reported. nc: not calculated.

Exp: Exponential culutre; Sta: Stationary culture; Green shading: significantly up-regulated; Red shading: significantly down-regulated.
