# Supplementary material for: Quantitative ‘Omics Analyses of Medium Chain Length Polyhydroxyalkanaote Metabolism in Pseudomonas putida LS46 Cultured with Waste Glycerol and Waste Fatty Acids
Source: PLoS One. 2015 Nov 6;10(11):e0142322. doi: 10.1371/journal.pone.0142322 (PMC4636370; doi:10.1371/journal.pone.0142322)
Supplement: S7 Table — (PDF) [file pone.0142322.s009.pdf]

S7 Table. Expression values of putative FadE homologs in *P. putida* LS46 grown in waste fatty acids (WFA) cultures during exponential phase, and variations in their expression levels under two conditions.

					Waste fatty acid vs Waste glycerol (Exp)	
**Locus Tag**	**Gene Anotation**	**DNA Seq Length (nt)**	**R[a]**	**P[a]**	**Rnet**	**Pnet**
PPUTLS46_000145	acyl-CoA dehydrogenase	2448	11.1	18	-0.7	0.15
PPUTLS46_000650	acyl-CoA dehydrogenase domain-containing protein	1167	7	nr	0.02	nc
PPUTLS46_000700	acyl-CoA dehydrogenase domain-containing protein	1776	8.5	nr	-0.16	0.08
PPUTLS46_002222	acyl-CoA dehydrogenase domain-containing protein	1770	7.5	nr	1.26	nc
PPUTLS46_002522	AcdA	1152	9.6	17.6	0.46	0.52
PPUTLS46_005901	acyl-CoA dehydrogenase	1803	10.2	nr	1.89	nc
PPUTLS46_006756	acyl-CoA dehydrogenase	1653	10.8	18.7	-0.33	0.59
PPUTLS46_007429	acyl-CoA dehydrogenase domain-containing protein	1128	nr	nr	nc	nc
PPUTLS46_008099	acyl-CoA dehydrogenase	1134	3.8	nr	0.06	nc
PPUTLS46_010244	acyl-CoA dehydrogenase	1230	nr	nr	nc	nc
PPUTLS46_012190	isovaleryl-CoA dehydrogenase	1164	9.2	17.2	0.15	-0.55
PPUTLS46_015999	glutaryl-CoA dehydrogenase	1182	11	17.6	0.24	0.09
PPUTLS46_018781	acyl-CoA dehydrogenase type 2	1188	6.3	nr	-0.84	nc
PPUTLS46_018861	type 2 acyl-CoA dehydrogenase	1233	5.1	nr	0.59	nc
PPUTLS46_024438	acyl-CoA dehydrogenase	1779	nr	nr	nc	nc
PPUTLS46_024448	acyl-CoA dehydrogenase	1806	16.6	24.3	3.25	3.54
PPUTLS46_025248	acyl-CoA dehydrogenase type 2	1182	4	nr	-0.66	nc
PPUTLS46_026101	acyl-CoA dehydrogenase domain-containing protein	1161	6.4	nr	1.87	nc

[a]: The log2 value of RNA and Protein expression abundance of *P.putida* LS46 grown in waste fatty acid derived culture at 8hrs post-inocula. RNA or Protein that were either not detected or only observed in one biological replicate were not reported for comparative analysis, and therefore no Rnet and Pnet value calculated. nr: not reported. nc: not calculated.

Exp: Exponential culture; Sta: Stationary culture. Green shading: significantly up-regulated.
