# Supplementary material for: Quantitative ‘Omics Analyses of Medium Chain Length Polyhydroxyalkanaote Metabolism in Pseudomonas putida LS46 Cultured with Waste Glycerol and Waste Fatty Acids
Source: PLoS One. 2015 Nov 6;10(11):e0142322. doi: 10.1371/journal.pone.0142322 (PMC4636370; doi:10.1371/journal.pone.0142322)
Supplement: S8 Table — (PDF) [file pone.0142322.s010.pdf]

S8 Table. Expression values of putative fatty acid-oxidation genes and gene products in *P. putida* LS46 grown in waste fatty acids (WFA) cultures during exponential phase, and variations in their expression levels under two conditions.

Clusters	Locus Tag	Gene symbol/annotation	DNA seq length (nt)	R[a]	P[a]	Waste glycerol Sta vs Exp		Waste fatty acid vs Waste glycerol (Exp)	
						Rnet	Pnet	Rnet	Pnet
1	PPUTLS46_004399	fadB	2148	16.1	25.5	1.54	0.52	4.4	4.65
	PPUTLS46_004404	fadA	1176	15.6	24.7	0.46	-0.69	3.79	4.8
2	PPUTLS46_007434	fadB1X	774	9.7	20.2	0.06	0.02	-0.33	0.03
	PPUTLS46_007429	fadEx	1128	nr	nr	nc	nc	nc	nc
	PPUTLS46_007424	fadAx	1194	nr	nr	nc	nc	nc	nc
	PPUTLS46_007419	fadB2x	768	nr	nr	nc	nc	nc	nc
	PPUTLS46_007414	fadDx	1674	7.7	nr	1.06	nc	-0.57	nc
3	PPUTLS46_000695	3-hydroxybutyryl-CoA epimerase	1239	10.4	nr	1.33	nc	0.23	nc
	PPUTLS46_000700	Acyl-CoA dehydrogenase putative	1776	8.5	nr	0.08	-1.68	-1.33	nc
	PPUTLS46_000705	Alcohol dehydrogenase iron-containing	1164	7.1	nr	nc	nc	-1.58	nc
	PPUTLS46_000710	Thioesterase superfamily protein	453	6	nr	-0.01	nc	-0.95	nc
	PPUTLS46_000715	Acetyl-CoA acetyltransferase	1185	8	nr	0.03	-1.86	-1.02	nc
4	PPUTLS46_026256	TetR family transcriptional regulator	582	9.6	nr	-0.04	nc	1.56	nc
	PPUTLS46_026251	3-hydroxybutyryl-CoA dehydrogenase	849	11.1	16.7	nc	nc	1.73	2.19
	PPUTLS46_026246	beta-ketothiolase	1185	11.7	20.8	0.06	0.47	1.66	1.76
Others	PPUTLS46_012993	acetyl-CoA acetyltransferase	1179	11.8	23.1	-0.6	0.53	7.51	6.7
	PPUTLS46_003422	3-hydroxybutyrate dehydrogenase	771	10.81	19.24	nc	nc	3.84	nc
	PPUTLS46_006916	acyl-CoA thioesterase II	870	10.05	14.51	-0.2	0.03	-0.2	-0.39
	PPUTLS46_009584	Acyl-CoA thioesterase-like protein	798	10.2	19.1	-0.64	0.79	0.16	3.71

[a]: The log2 value of RNA and Protein expression abundance of *P.putida* LS46 grown in waste fatty acid derived culture at 8hrs post-inocula. RNA or Protein that were either not detected or only observed in one biological replicate were not reported for comparative analysis, and therefore no Rnet and Pnet value calculated. nr: not reported. nc: not caluculated.

Exp: Exponential culture; Sta: Stationary culture. Green shading: significantly up-regulated; Red shading: significantly down-regulated.
